# Continuous-Flow Synthesis of Primary Vinylarenes via Inline Grignard Reagent Formation and Peterson Olefination

**DOI:** 10.1021/acsomega.5c00823

**Published:** 2025-04-25

**Authors:** Matthew J. Burrell, John R. Varcoe, Daniel K. Whelligan

**Affiliations:** School of Chemistry and Chemical Engineering, University of Surrey, Guildford GU2 7XH, U. K

## Abstract

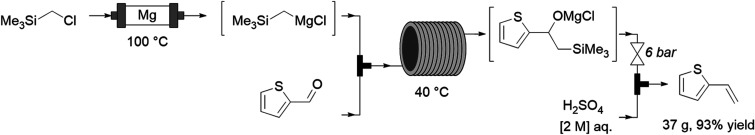

Primary vinylarenes are important monomers for the production of materials, which in our case are ion exchange membranes for electrolyzers. Given the high cost of certain vinylarenes but the relative affordability of their aldehyde precursors, we explored their synthesis using flow chemistry to enable facile and safe scale-up. While a soluble, methanolic Wittig reaction found limited success, an alternative approach involving Peterson olefination was high-yielding. This required (trimethylsilyl)methyl Grignard reagent, which was generated in flow using a magnesium-filled column. Thus, 2-vinylthiophene was obtained in 93% yield at 37 g scale, and the route was applicable to other nonpolar arenes. For polar arenes, precipitation at the oxymagnesium chloride stage and inefficient elimination were observed, but these challenges could be mitigated by employing (phenyldimethylsilyl)methyl Grignard reagent instead and stronger acid at a higher temperature for the elimination.

## Introduction

Primary vinylarenes find extensive use as monomers in radical, cationic, and anionic polymerizations, with the most prevalent example being styrene and its radical or anionic conversion into polystyrene.^1^ Another example is poly(vinylphenol), which is often used with comonomers in microelectronics, photoresistors, and biocompatible polymers, and is accessed via radical or cationic polymerization of vinylphenol or in two steps from styrene-*t*-butyl ether.^2,3^ In terms of vinylheteroaromatics, poly(vinylpyridine) (and its copolymers), by virtue of its coordinative nitrogen atom, can complex metal ions and is therefore used in catalytic and cargo-releasing materials.^4^ Its mild basicity permits use in pH-sensitive systems, and it is often quaternized, through alkylation, to give a cationic polymer, which is used as an anion exchange resin^5^ and even has antiviral and antibacterial properties.^6^ Poly(1-vinylimidazole) has been shown to transport therapeutic RNA,^7^ be transformable into fluorescent carbon nanocrystals,^8^ and be convertible into *N*-heterocyclic carbenes for reaction catalysis,^9^ among many other applications.^10^ Much less reported is the polymerization of 2-vinylthiophene, but one recent example studied the product’s dehydrogenation into a conducting polymer on the surface of silica, and another found a syndioselective method of polymerization since such polymers may find use as conductive or fluorescent materials.^11,12^

In addition to use as monomers, vinylarenes can act as substrates for further synthetic manipulations such as asymmetric hydroamidation/-amination (in Markovnikov and anti-Markovnikov fashion),^13,14^ hydroboration,^15^ hydrosilylation,^16^ and even photocatalytic hydrotrifluoromethylation,^17^ among others.^18^

As part of ongoing research into anionic exchange membranes for use in fuel cells and electrolyzers,^19−21^ we required access to decagram quantities of 2-vinylthiophene (**3**) for production of copolymer membranes with potentially improved properties,^22^ based on a report by Wang *et al*.^23^ 2-Vinylthiophene was prohibitively expensive from commercial sources, but thiophene-2-carboxaldehyde (**1**) was relatively inexpensive. We therefore embarked on a research project to convert the latter into the former using flow chemistry because this permits safe and facile scale-up as well as rapid reaction optimization through easy adjustment of variables.^24^ Both the Wittig reaction and the Peterson olefination were investigated, so each is reviewed in terms of flow chemistry in the following paragraphs.

Several flow versions of the Wittig reaction have been reported, but all employ stabilized ylides with mild bases such as hydroxide or carbonate.^25,26^ The process usually involves premixing the phosphonium salt and aldehyde and adding a separate solution of base into the flow reactor where the reaction takes place. For the synthesis of primary vinylarenes, we required non-stabilized ylide formed from methyltriphenylphosphonium halide **2** (p*K*_a_ in DMSO 22.5).^27^ To form this in batch, a stronger base such as BuLi or KO^t^Bu tends to be used, usually in the solvent THF, and at temperatures of −78 and 0 °C, respectively.^28,29^ The weaker, neutral base DBU has also been employed at reflux in DCM (40 °C).^30^ However, these reactions, with the required methylphosphonium salt in THF, are described as forming a slurry rather than a solution, making them largely incompatible with flow chemistry. The use of ethanol as a solvent has been described in the literature for aryl-stabilized ylides using the bases LiOEt and NaOEt, and so it is assumed that the reaction would also be compatible with methanol (and base MeONa).^31,32^

For the Peterson olefination, β-silyl alcohols are required for acid- or base-catalyzed elimination to the alkenes. The β-silyl alcohols can be accessed through silylmethyl Grignard reagent addition to aldehydes. Leadbeater published a continuous-flow Peterson olefination to produce trifluoromethylvinylarenes, and this is of most relevance to the work we report here.^33^ They reacted a solution of pre-made Grignard reagent with aldehyde to deliver the β-silyl alcohol after an inline aqueous quench. The report describes optimization of the use of a membrane-based liquid–liquid separator to facilitate an inline extraction/solvent swap to hexane/DCM (9:1)—the solvent system required for use of Lewis acid TMSOTf to induce elimination. In contrast, we wished to generate the Grignard reagent from magnesium metal in flow and planned to use inexpensive sulfuric acid for a final combined aqueous quench and elimination to give (non-trifluoromethylated) vinylarenes.

Regarding the generation of Grignard reagents in flow, this has been reported in the literature in two main ways:

1. By halogen-metal exchange reaction. For example, Deng *et al*. used EtMgBr to exchange with trifluorobromobenzene before reaction with CO_2_, and von Keutz *et al*. used “turbo Grignard” ^i^PrMgCl.LiBr to convert chloroiodomethane into chloromethylmagnesium chloride—a highly unstable magnesium carbenoid—in a micromixer for 1 s at −20 °C and reacted it with an aldehyde in the following 1.6 s.^34,35^
2. By insertion of Mg metal into an organohalide bond. The main practical flow methods for this can be categorized into use of packed bed column reactors and continuous stirred tank reactors (CSTRs), but several methods exist between the two. A brief review of these follows.

A column-like reactor involving a complex setup of glassware packed with stationary Mg turnings was first described in 1960 to prepare vinylmagnesium chloride.^36^ Similar has been built on an industrial scale for the preparation of EtMgCl, which was used in the production of ethylsilanes.^37^ Use of a modern, laboratory-scale commercial flow reactor was reported in 2017 by Hoz and Alcazar who optimized Grignard reagent formation through a glass column packed with Mg particles (20–230 mesh = 0.84–0.063 mm).^38^ A focus was placed on the Mg-activation method before a range of haloarenes and haloalkanes were converted into their corresponding Grignard reagents, in a 0.5 M LiCl solution of THF, and reacted with several electrophiles in flow. This method has subsequently been used to conduct a telescoped Grignard reagent formation and Fe-catalyzed C(sp^3^)-C(sp^2^)/C(sp) coupling.^39^ In 2020, the company Fraunhofer IMM reported the use of a jacketed (for temperature control) metal column reactor, manufactured by 3D laser-melting, containing Mg turnings pre-activated by mechanical agitation or ultrasound to abrade the surfaces.^40^ An attachment for manual replenishment of Mg during the flow reaction was also added, which was relatively facile because the flow reaction was not under pressure. Grachev *et al*. described a temperature-controlled jacketed column reactor (which they first built and used in 1984) that included a multiblade stirrer and separator.^41^ In 2011, they reported its use in optimizing Grignard reagent formation with 1–3 mm Mg granules in toluene/(Et_2_O or THF) mixtures, finding that 1 equiv. of Et_2_O or THF to organohalide was required for high yield. A fluidized-bed reactor, which can perhaps be considered a hybrid between the packed-bed column and stirred column reactors, was described by Duchateau in 2016.^42^ This involved selection of Mg particle size and flow rate such that the suspension behaved like a fluid and was used to produce PhMgBr quantitatively as determined by inline monitoring by NMR.

A CSTR involves drawing reaction/product mixture from a batch-like vessel while replacing starting materials at the same rate and so must operate near completion of the reaction. This apparatus allowed Eli Lilly to carefully control the temperature that was necessary to produce tetrahydropyranmagnesium chloride and react it with the requisite amide although, rather than continuous flow, the Grignard reagent was moved to the next reactor in boluses once per min using an automated pressure swing cylinder.^43^ Regardless, owing to the low solubility of the Grignard reagent, this was changed to a Barbier reaction (electrophile premixed with organohalide before Mg-insertion) to consume it as it was produced, before precipitation. Later still, this was made completely continuous by installing a dip tube for the removal of product via a settling pipe to prevent fine particles from exiting.^44^ They had previously described this setup for the production of an aryl Grignard reagent.^45^

## Results and Discussion

### Wittig Reaction

**Scheme 1:**
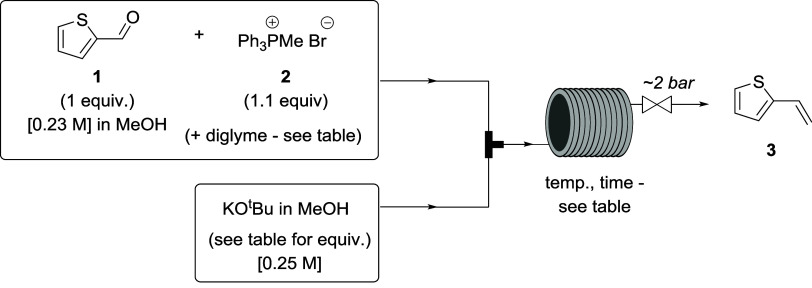
Attempted Flow Wittig Reaction for Synthesis of 2-Vinylthiophene (**3**)

As described in the Introduction, sodium methoxide was chosen as a base and methanol as a solvent to facilitate complete dissolution of the reactants, especially methyltriphenylphosphonium bromide (**2**) (Scheme 1), for application to flow chemistry.^31,32^ The KBr by-product solubility in methanol at 25 °C is 2.06 g/100 g (0.14 M), so the reaction concentration was kept below this molarity to avoid precipitate-mediated blockages.^46^ In this way, several conditions were varied, and since GCMS of the crude product mixture showed only starting materials and desired product, peak area ratios were used to judge the extent of each reaction (Table 1).

**Table 1:** Variations in Flow Wittig Reaction to Form 2-Vinylthiophene (**3**)^a^

entry	diglyme additive (equiv)	KO^t^Bu equiv	temp. (°C)	residence time (min)	GCMS EIC peak area ratio (product: aldehyde)^b^
1	0	1.1	RT	30	0:100
2	0	1.1	50	30	0:100
3	0.1	1.1	50	10	19:81
4	1.2	1.1	50	10	57:43
5	2.2	1.1	50	10	48:52
6	1.2	1.1	40	10	50:50
7	1.2	1.1	60	10	54:46
8	1.2	1.1	60	20	46:54
9	1.2	1.1	90	10	38:62
10	1.2	1.1	110	10	29:71
11	1.2	2.1	50	10	52:48
12^c^	1.2	1.1	50	10	33:67^c^

a Reactant concentrations and equiv are given in Scheme 1.

b Peak area from extracted ion count (EIC) chromatogram of molecular ions—see Supporting Information.

c Entry 12: reactant solutions were not prepared fresh but stored in solution for >24 h prior to reaction.

The results show that no vinylthiophene **3** product was formed until diglyme was added at 0.1 equiv (entry 3), and increasing its amount to 1.2 equiv led to 57% GCMS peak area ratio at the same temperature (entry 4).^47^ This is likely due to the enhancement of the basicity of KO^t^Bu, through chelation of its counterion, rather than enhancement of the nucleophilicity of the ylide because it has been shown that potassium diphosphanylmethanides lose their coordination to diglyme in THF.^48^ Further increasing the amount of diglyme reduced the conversion (entry 5) as did reaction temperatures lower or higher than 50 °C (entries 6, 7, 9, and 10). An extended reaction time at 60 °C (entry 8) worsened product formation at this same temperature. An increase in the amount of base (entry 11) showed no significant change in conversion, and entry 12 shows that pre-mixed aldehyde **1** and phosphonium bromide **2** could not be stored in solution for more than 24 h as this led to a drop in product formation. Overall, since for starting aldehyde **1** and vinylthiophene **3**, the GCMS peak area ratio approximately equals molar ratios,^a^ and the Wittig reaction could not be made to progress beyond the halfway point, this reaction was abandoned in favor of *in situ* Grignard reagent formation and Peterson olefination.

### Grignard Reagent Formation and Peterson Olefination

**Scheme 2:**
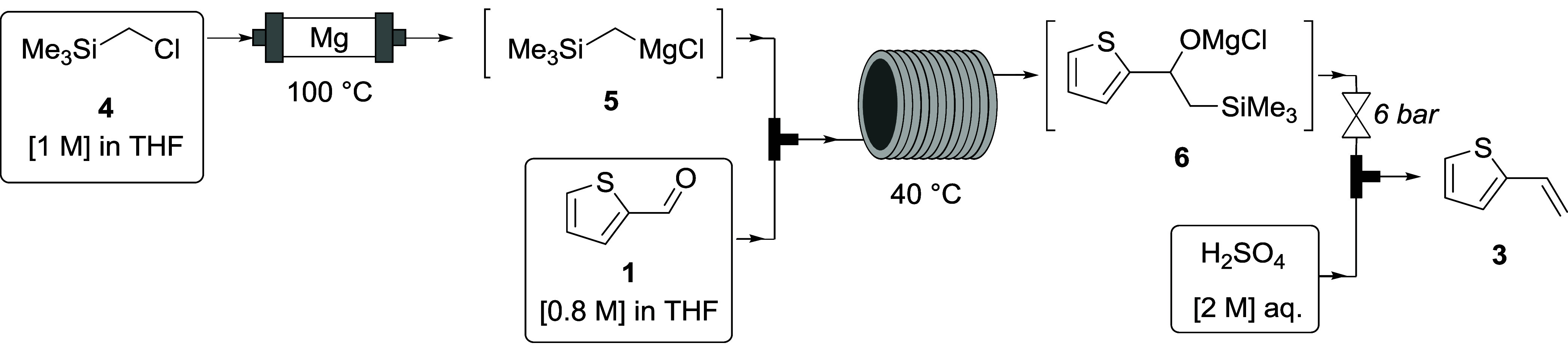
Continuous Flow Grignard Reagent Formation and Peterson Olefination

Our proposed flow route (Scheme 2) to the Peterson olefination involved conversion of trimethylsilylmethyl chloride (**4**) into its Grignard reagent **5** before addition to the aldehyde **1** to give an oxymagnesium intermediate **6**. Treatment of this with acid would lead to elimination to give the desired alkene **3**.

In our case, a packed bed column reactor of Mg turnings was chosen due to the commercial availability of the equipment (an Omnifit glass column with a Vapourtec heating jacket and thermostat, Figure S2) and ease of setup and handling on the lab scale. For test reactions, the output stream was collected in vials using a fraction collector. The inline addition of H_2_SO_4_ performs the triple purpose of quenching the oxymagnesium species, promoting an acid-catalyzed elimination, and dissolving magnesium salts. Flow rates of the organohalide and aldehyde were kept equal, but their concentrations were such that there were 1.25 equiv of the silane compared to the aldehyde. The Mg-filled column was heated at 100 °C to ensure rapid formation of the Grignard reagent, but this was not optimized. Any excess would be converted to highly volatile tetramethylsilane on acid quench.

The first hurdle encountered was repeatability of the apparent “activation of the Mg turnings” where the method of Hoz and Alcazar was tested (DIBALH in toluene followed by a mixture of Me_3_SiCl and 1-bromo-2-chlorobutane in THF/toluene (1:1)).^38^ It should be noted that this procedure may not be “activation of the Mg” *per se* but entrainment of the Grignard reagent formation through production of catalytic MgX_2_.^49,50^ In our case, when using a manual syringe to add this solution and letting it sit for at least 10 min at different temperatures (20–40 °C), an unidentified precipitate was observed, presumably the same described by Hoz and Alcazar; so to reduce this, a continuous flow of the activation mixture was used but still via the syringe. However, this led to less repeatable activations and low yields, which we ascribed to the (manual via syringe) flow rate being too high. Finally, the flow reactor was used to pump the activation solution at 1 mL/min by virtue of its valve-switchable inline sample loops. This had the added advantage of preventing any ingress of air when switching from the activation solution to reactant bottles, as valve-switching was instantaneous. During this investigation, it was also found that 1-bromo-2-chlorobutane could not be replaced with 1,2-dibromoethane and achieve the same repeatability, but the use of DIBALH could be omitted. Thus, our standard activation procedure became pumping approximately 1 column volume of a mixture of 1-bromo-2-chlorobutane (0.24 M) and Me_3_SiCl (2 M) in THF/toluene (1:1) through the column of Mg at a flow rate of 1.0 mL/min using the machine’s sample loops.

A second change to the hardware was made to prevent repeated blockage of the 30 μm PTFE frit at the fluid exit of the Mg column, presumably by either Mg finings or continued fine precipitate. Herein, the frit was switched for a stainless-steel mesh, which allowed any fine solids through, and they caused no problems in the remaining flow reactor tubing. If they were Mg finings, they would be consumed in the sulfuric acid quench and, if inorganic salts, removed in the aqueous layers.

**Scheme 3:**
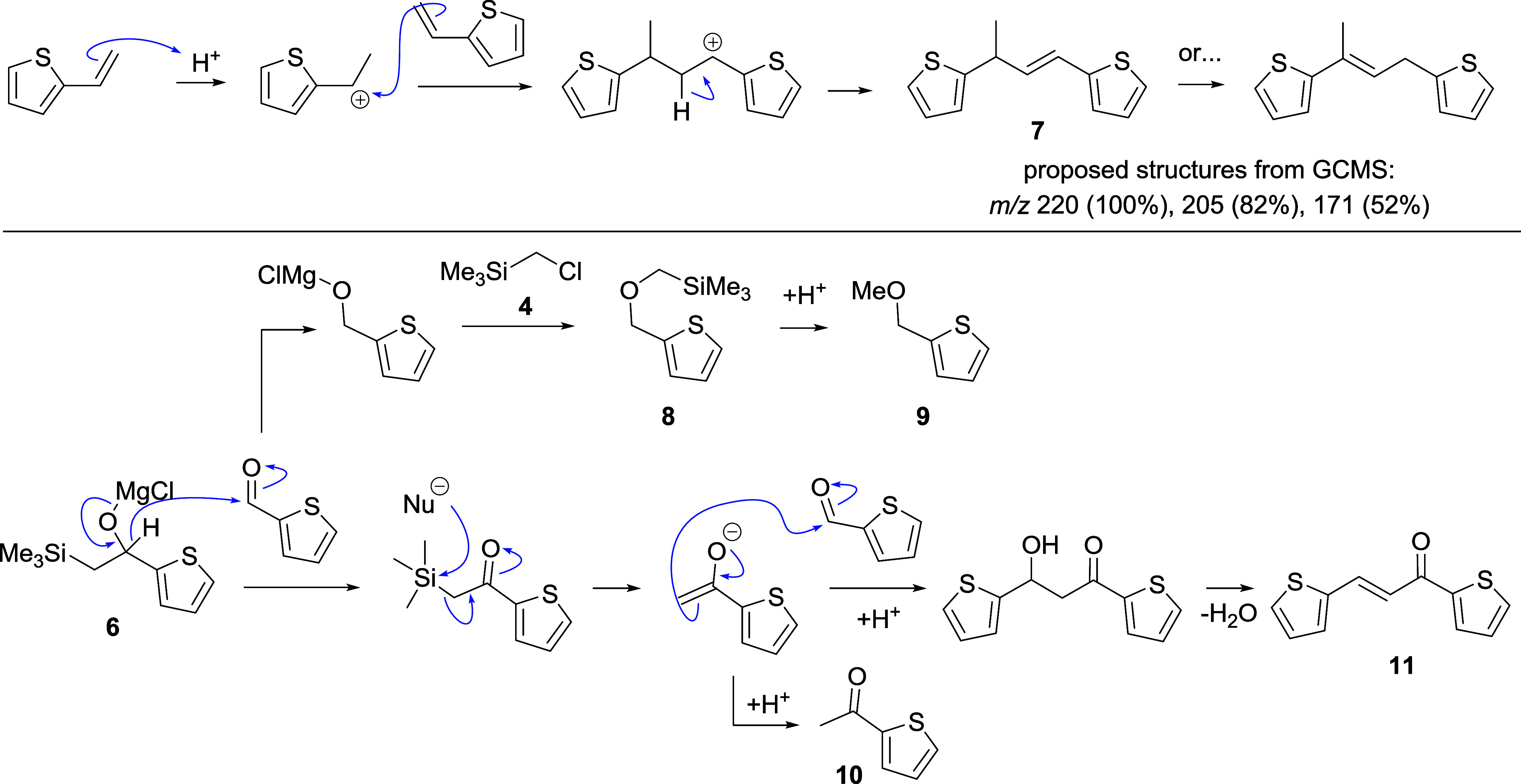
Proposed Mechanisms for the Formation of Side Products during Peterson Olefination

An initial successful test reaction to product vinyl thiophene **3** was confirmed by GCMS, but small amounts (each 1–5% by GCMS peak area) of side products 2-methoxymethylthiophene (**9**) and 2-acetylthiophene (**10**) (Scheme 3) were also observed (Figure S4, ESI). In addition, a compound with *m*/*z* 220 and fragment *m*/*z* 205, indicating loss of a methyl group, is very tentatively identified as the product **7** (or its isomer) of acid-catalyzed dimerization of vinylthiophene. The mechanism of formation of compounds **9** and **10** is discussed below in the context of the next experiment.

**Figure 1:**
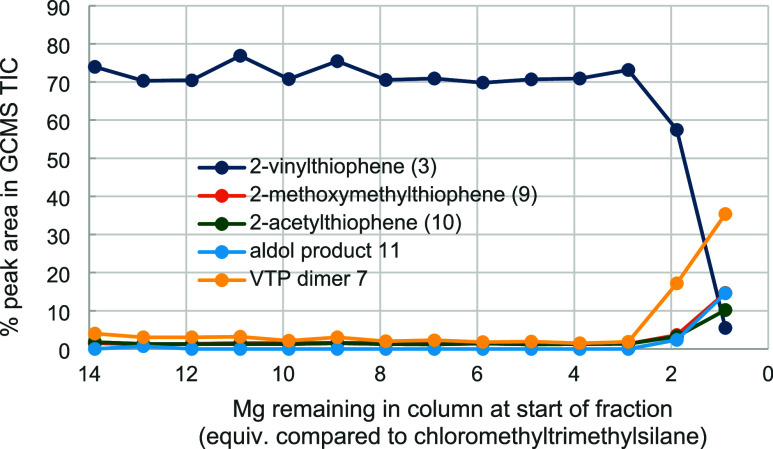
Effect of Mg consumption on product and side-product formation over time is shown as GCMS total ion count (TIC) chromatogram peak area percentages.

We were concerned that as Mg was consumed during the reaction, the effective residence time of the halide over Mg metal would reduce and, at later time points, the Grignard reagent would not be formed to completion. To assess this, 14 fractions were taken, beginning after steady state was achieved, over the course of a 3 g (24.5 mmol) scale flow synthesis where insufficient Mg (22.0 mmol, 0.9 equiv) was purposefully provided in the column. The results of GCMS analysis of these fractions are shown in Figure 1 and reveal that the decreasing contact time does not affect the yield of vinylthiophene **3** until there remains less than three equivalents of Mg, indicating rapid Grignard reagent formation at 100 °C. Interestingly, at this point, there was an increase in methyl ether **9**, ketone **10**, and enone **11** (Figure S4). These are proposed to form via a Canizzaro-like disproportionation (Scheme 3) followed by, in the case of **11**, aldol reaction, and in the case of ether **9**, alkylation by remaining trimethylsilylmethyl chloride to give **8**, which undergoes protodesilylation on meeting sulfuric acid. Thus, as the concentration of the Grignard reagent decreases, aldehyde is left unconsumed and available for these steps. However, possible dimer **7** is also seen with an increased peak area, which casts doubt on our tentatively assigned structure.

The synthesis of vinylthiophene **3** was next repeated on 34 mmol (3.8 g) scale with near complete reaction by GCMS. However, workup/purification required investigation because azeotrope formation between vinylthiophene **3** and THF led to the loss of large amounts of product by rotary evaporation or distillation. Thus, after reaction completion, the mixture was transferred to a separating funnel, including rinses with dichloromethane, and the organic layer was separated from the sulfuric acid aqueous layer, washed with brine and then weighed, and analyzed by ^1^H NMR to determine the molar content of product and THF. This was reassessed after various purification attempts, so the remaining percentage of product and percentage removal of THF could be determined (Table 2). Entries 1 and 2 confirm the loss of product by azeotrope formation, and entry 3 shows that the addition of hexane to aid the evaporation of THF through azeotrope formation was partially successful but still reduced product yield significantly. Focus therefore turned to methods of washing to remove THF instead. The addition of Et_2_O to help retain the vinylthiophene and washing it with water 15 times left 63% of the THF (entry 4). Use of an aqueous LiCl solution, thought to possibly coordinate to THF and retain it in the aqueous phase as it does with DMF,^51^ gave inferior results unless diethyl ether addition was omitted (entries 5 and 6). The alternative salt MgSO_4_ led to similar vinylthiophene retention and greatly improved THF removal (entry 7). Use of hexane as an additive to the organic layer, however, made both criteria worse (entries 8 and 9), and in the end, simply washing the neat organic layer with pure water was most effective (entry 10). Using this method on a 37 g scale (68 h flow reaction), followed by vacuum distillation afforded vinylthiophene **3** in 93% yield. This yield compares favorably with equivalent batch Peterson olefinations to vinylthiophene **3** reported in the literature: 71% (2.4 g, 2 steps) using 1 M aq. HCl for elimination at RT,^52^ 87% (1.1 g, 2 steps) using six drops of conc. sulfuric acid for elimination at reflux in THF,^53^ and 60% (NMR tube scale elimination, 2 steps) using 0.1 mol % HNTf_2_ at RT.^54^ The scale of the flow reaction is only limited by the size of the magnesium column although even this could be overcome using in-line column changes by valve switching, as has been reported in the literature.^55^

**Table 2:** Results of Various Methods of Purification of Vinylthiophene **3** Performed Post Sulfuric Acid (2 M) Quench and Aqueous Sulfuric Acid Layer Removal^a^

entry	solvent added to organic layer^b^	purification method	washes (each ∼20% of initial volume of organic layer)	remaining vinylthiophene **3** (mol %)	THF removed (mol %)
1		vacuum distillation	N/A	12	100
2		rotary evaporation	N/A	23	77
3	hexane ×4	rotary evaporation ×4	N/A	66	98
4	Et_2_O	washes	Water × 15	93	63
5	Et_2_O	washes	LiCl (sat. aq.) ×15	88	52
6		washes	LiCl (sat. aq.) ×15	96	73
7		washes	MgSO_4_ (sat. aq.) ×15	90	90
8	hexane	washes	MgSO_4_ (sat. aq.) ×15	69	69
9	hexane	washes	water ×15	72	60
10		washes	water ×15	94	96

a Performance is measured in terms of remaining vinylthiophene **3** (mol%) and amount of THF removed (mol%) compared to the original organic layer. Data were calculated using the masses of the crude organic layer and purified product along with integrals of ^1^H NMR peaks corresponding to product and THF.

b After quench/reaction with sulfuric acid (2 M) and transfer to a separating funnel using dichloromethane rinses, the organic layer was separated, washed with brine and a solvent was added at ∼ 20% volume of that organic layer. The purification method described in the table was then applied to this layer.

To show the scope of this method, five other vinyl arenes **12–16** were targeted, varying in polarity and/or expense compared to their starting aldehydes (Table 3).

**Table 3:** Comparison of the Price of the Vinylarene Product Compared to the Price of the Respective Aldehyde Starting Material from Merck in March 2025^a^

product	product price (/g)	starting material price (/g)
2-vinylfuran (**12**)	£960.95	£0.13
3-methylstyrene (**13**)	£12.20	£0.71
methyl 4-vinylbenzoate (**14**)	£3.83	£0.07
1-methyl-2-vinylimidazole (**15)**	£6360.00	£3.29
3-vinylpyridine (**16**)	£23.92	£0.60

a The lowest priced grade of material in the highest available quantity was used.

**Scheme 4:**
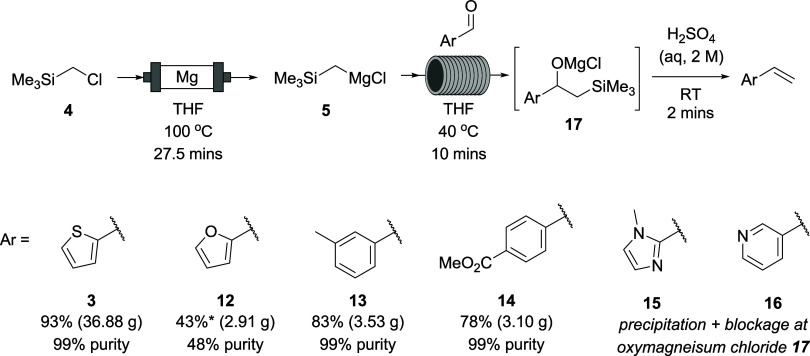
Flow Synthesis of 2-Vinylfuran (**12**), 3-Methylstyrene (**13**), and Methyl 4-Vinylbenzoate (**14**) *Yield takes account of impurity, which is predominantly THF.

For relatively nonpolar aryl groups furyl, tolyl, and 4-methoxycarbonylphenyl, the method was successful without modification (Scheme 4). After water washes and distillation of the product, the vinylarenes **12–14** were obtained; however, complete removal of THF and by-product Me_3_SiOH from vinylfuran **12** proved challenging, resulting in a product of only 48% purity by NMR. Of particular interest is the survival of the ester group of arene **14** in the presence of excess Grignard reagent (1.25 equiv) to give a product in similar yield (78%) to **13** at this scale (83%) indicating the ester may be unreactive to Grignard reagent in the 10 min reaction time at 40 °C.

**Scheme 5:**
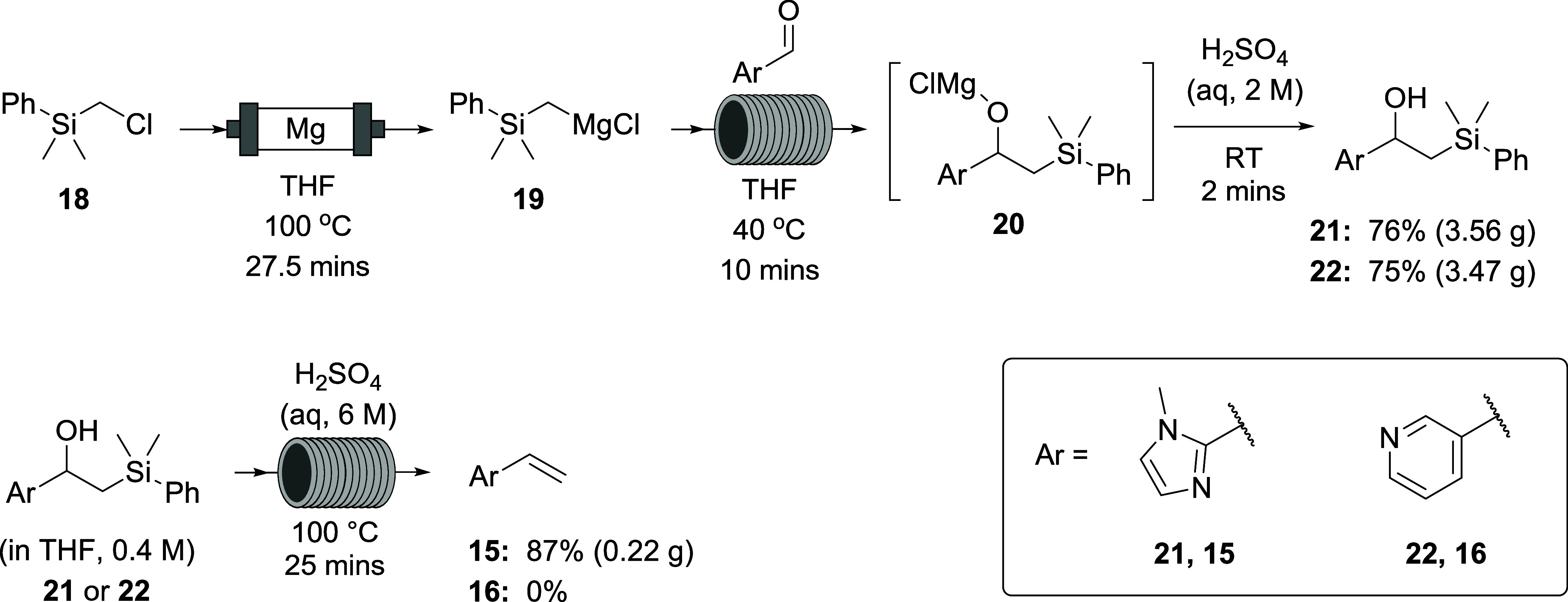
Use of Phenylsilane **18** for Grignard Formation and Aldehyde Addition to Increase Hydrophobicity and Avoid Precipitation of Oxymagnesium Chloride Intermediates **20** When More Polar Arenes Are Used

In the attempted flow syntheses of 1-methyl-2-vinylimidazole (**15**) and 3-vinylpyridine (**16**), however, a blockage formed in the tube reactor after the addition of aldehyde. This was thought to be due to precipitation of the respective oxymagnesium chloride intermediates **17** which we hypothesized was due to the imidazole and pyridine groups making the overall polarity of the molecule too high for dissolution in THF. A similar problem has been reported for the addition of vinyl Grignard reagent to amides and was solved by the use of a plate reactor bearing a hydrophobic coating to prevent the precipitate from clogging.^56^ In the absence of such equipment, we made alternative investigations. For an unknown reason, the addition of dimethoxyethane (DME, 2 equiv) to the solution of aldehyde appeared to slightly delay the point at which precipitation appeared in the reactor tubing but did not solve the problem. Attention therefore turned to the silyl component of the intermediate with a view to increasing its hydrophobicity to counter the polarity of these aryl groups. Thus, (chloromethyl)dimethylphenylsilane (**18**, Scheme 5) was used to form the Grignard reagent **19** for addition to the aldehyde. No precipitation occurred at 0.4 M concentration; however, the elimination did not take place upon the standard addition of 2 M H_2_SO_4_ (aq) at RT, and the silyl alcohols **21** and **22** were isolated instead.

**Scheme 6:**
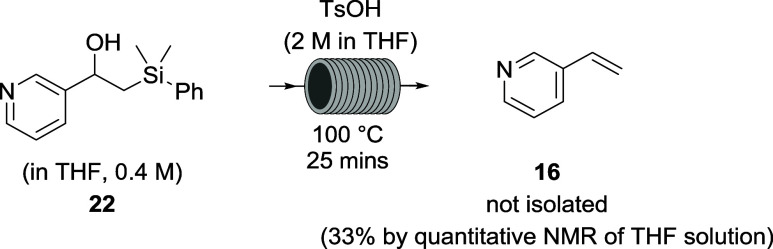
Use of Non-Aqueous Acid TsOH (2 M in THF) for Flow Elimination of Pyridylsilyl Alcohol **20**

In the absence of a third pump and second tubing reactor (in previous reactions, a separate syringe pump, after the back pressure regulator, was being used to add H_2_SO_4_),separate flow reactions (Scheme 5) were conducted on isolated silyl alcohols **21** and **22** to determine conditions for elimination to the alkene. The use of more concentrated (6 M) H_2_SO_4_ (aq) and a temperature of 100 °C led to complete conversion, providing vinylimidazole **15** in 87% yield after purification by column chromatography (66% over the two steps from the aldehyde). For vinylpyridine **16**, however, no product was obtained, despite multiple extractions of the basified aqueous layer (pH ∼ 12) with DCM. Hypothesizing that the product may have been lost to the aqueous layer, a non-aqueous elimination was attempted using organic-soluble toluene sulfonic acid (TsOH) in THF (2 M) (Scheme 6). Using an internal standard for quantitative NMR (qNMR), the yield was determined to be 33%, but this product could not be isolated by column chromatography or distillation presumably due to polymerization or other decomposition (despite the addition of 1000 ppm of *tert*-butylcatechol inhibitor).

**Scheme 7:**
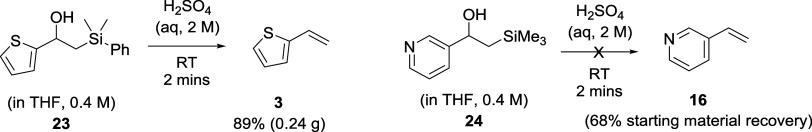
Experiments to Determine Whether Phenylsilane or Electron-Deficient Aryl Groups Inhibit Elimination

The requirement for harsher conditions to mediate the elimination of silyl alcohols **21** and **22** could be due to the nature of the aryl groups and/or the use of phenyldimethylsilane instead of trimethylsilane. This was investigated as follows (Scheme 7): thiophenyl(phenyldimethylsilyl) alcohol **23**, whose analogous trimethylsilane **6** was previously eliminated easily, and pyridyl trimethylsilyl alcohol **24**, whose analogous phenylsilane did not previously eliminate easily, were prepared. The former was prepared in flow using the method for silyl alcohols **21** and **22** except it was quenched with water instead of acid to avoid elimination. Regardless, NMR of the crude material showed it had undergone approximately 20% spontaneous elimination (already indicating that the phenylsilane is likely not responsible for inhibiting the acid-catalyzed elimination). Pyridyl alcohol **24** was prepared similarly but using a mixture of batch and flow conditions—the Grignard reagent was generated in flow but collected and added to a solution of 3-pyridinecarboxaldehyde in THF in a batch reaction where precipitation of the polar oxymagnesium chloride would cause no problems. Each isolated silyl alcohol was subjected to the original 2 M H_2_SO_4_(aq) flow treatment with a residence time of 2 min at 25 °C. As previously shown, isolation of vinylpyridine **16** after use of an aqueous solution is difficult. Therefore, to ascertain how easily hydroxysilane **24** eliminated, the recovered mass of hydroxysilane **24** starting material was used instead, and this was 68%, presumably indicating only ∼32% elimination despite the trimethylsilane being used.

Together, these results show that, rather than the presence of a phenyl ring on the silane, it is the nature of the aryl group that inhibits acid-catalyzed elimination. This is likely due to destabilization of cationic intermediates (or partially cationic transition states) in the acid-catalyzed elimination mechanism by electron poor arenes such as pyridine and imidazole.^57^

## Conclusions

In contrast to an attempted flow Wittig reaction, a flow Grignard reagent formation, addition to aldehyde and Peterson olefination, has been shown to be an efficient means to access up to tens-of-gram quantities of nonpolar, primary vinylarenes, important monomers for various polymerizations. The scope included furan and thiophene as the arene as well as methyl- and ester-substituted benzene. However, with more polar heteroaromatics, in this case pyridine and methylimidazole, intermediate oxymagnesium chlorides suffer from reduced solubility in THF. This problem is circumvented through the use of a phenyldimethylsilyl group in place of the usual trimethylsilyl group. These polar aryl-phenylsilane derivatives suffer from reduced elimination to the alkene; this is shown not to be the fault of the phenylsilyl group, but it is rather the electron-withdrawing nature of the polar aryl groups tested that necessitates the use of more forcing acidic elimination conditions.

## Experimental Section

### General Experimental

#### Solvents and Reagents

All reactants were purchased from commercial sources and used without purification, unless otherwise stated. Anhydrous THF was inhibitor-free and purchased from Sigma-Aldrich in bottles with septa attached or prepared by passage through an activated alumina column, using a Pure Solv Micro Solvent Purification System and storage over activated molecular sieves (3 Å, 8 to 12 mesh).^58^ Standard Schlenk line techniques were used for the preparation of solutions ready for plumbing to the flow reactor.

#### Purification and Chromatography

Flash chromatography was performed on a Biotage Selekt Flash Purification system with purchased Biotage or Modus pre-packed cartridges containing 40–60 μ 60 Å silica gel.

#### Characterization

FTIR spectra were recorded on a PerkinElmer Spectrum Two FTIR Spectrometer with a LiTaO_3_ detector. Selected transmittance minima have been recorded in wavenumbers (cm^–1^). ^1^H NMR and ^13^C NMR spectra were recorded in CDCl_3_ on either a Bruker Ascend 400 or a Bruker Ultrashield 500 MHz spectrometer. Chemical shifts (δ) are reported in parts per million (ppm) referenced to a residual protic chloroform signal at 7.26 ppm or tetramethylsilane (0.00 ppm). The data are given as follows: chemical shift (δ) in ppm, multiplicity, coupling constants J (Hz), integration, assignment (where given assignment was made using COSY, HSQC, and HMBC, see ESI). Coupling constants are accurate to 0.5 Hz. GCMS chromatograms and spectra were recorded on an Agilent Technologies 8890 GC system connected to an Agilent Technologies 5977B MSD operating in EI mode, and the conditions were as follows: inj. vol. of 1 μL, inj. temp. of 280 °C, column Agilent HP-5MS (30 m × 0.25 mm), carrier gas (H_2_) flow of 2 mL min^–1^, and oven temperature gradient of 0–3 min, 50 °C; 3–11 min, 50–130 °C (10 °C/min); 11–17 min, 130–250 °C (20 °C/min). Agilent MassHunter Quantitative Analysis (version 10.1) was used to generate extracted ion count (EIC) chromatograms and integrate their peaks to generate the GCMS peak area ratios given in Table 1. High-resolution LCMS chromatograms and spectra were recorded using an Agilent 1260 Infinity II HPLC coupled to an Agilent 6550 iFunnel QTOF mass spectrometer using electrospray ionization (Jet Stream Technology). LC conditions used an Agilent EC-C18 column (100 × 2.1 mm, 2.7 μm particle size), column temp. of 30 °C, flow rate of 0.30 mL min^–1^, mobile phase Solvent A: water (0.1% formic acid for ESI+ only), Solvent B: MeCN (0.1% formic acid for ESI+ only), gradient of 0–5.5 min, 5–100% B; 5.5–6.5 min, 100% B; 6.5–7.5 min; 100–5% B; 7.5–8.0 min, 5% B.

#### Flow Configuration

Reactions were carried out using a Vapourtec R4 reactor module coupled with the Vapourtec R2S peristaltic pump module. The peristaltic pumps were calibrated at the start of each month. For the formation of the Grignard reagent, an Omnifit glass column reactor (either 6.6 mm internal diameter (i.d.) ×150 mm or 15 mm i.d. ×150 mm) was packed with magnesium turnings, and the upper frit was replaced with a stainless-steel mesh (0.4 mm aperture). This column was held inside an air-heated glass jacket. Reactor coils (tube reactors) were made of perfluoroalkoxy alkane (PFA) and had internal volumes of either 2 or 10 mL and were held in an air-heated glass jacket. Mixing of the reactant streams was performed with T-connectors. Prior to running an experiment, the system was primed and flushed with the respective solvent (anhydrous THF or water). Pressure of the flow system up until the end of the tubing reactor was maintained at 6 bar with a fixed back pressure regulator. The temperature and pressure of the flow reactors were monitored in real time. The addition of acid solutions was by a syringe pump after the back pressure regulator. Software (Vapourtec Flow Commander v1.12.1.11) was used to take account of the dispersion of reactant solutions through the tubing, after switching pumps from solvent to reactants, and predict when full contribution of each reactant was achieved; this is termed “steady state.” At this point, the collection began—see example in Figure S3.

#### Wittig Reaction Procedure

See ESI.

#### Yield Calculation

The maximum theoretical product moles were determined using the concentrations of the reactant solutions, pump flow rates, and total product solution collection volume. The percentage yield was determined using the mass isolated and molecular weight, and the molar ratios of major impurities were determined by ^1^H NMR spectroscopy.

#### Magnesium Activation

Prior to generating Grignard reagents, activation of the magnesium turnings within the Omnifit glass column (Figure S2) was carried out at 30 °C using a mixture of 1-bromo-2-chloroethane (0.24 M, 1.2 mmol) and trimethylsilyl chloride (2.0 M, 10 mmol) in THF/Toluene 1:1 (5 mL, ∼2 packed column reactor volumes, 2.8 min residence time). The mixture was added from the flow reactor’s sample loops and pumped through the column of magnesium by valve-switching from the solvent reservoir to the sample loop before switching back prior to starting the reaction. This method eliminates the ingress of air or moisture.

### Grignard Reagent Formation from ClCH_2_SiMe_3_ and Peterson Olefination (General Procedure 1)

An Omnifit 6.6 × 150 mm glass column reactor was charged with magnesium turnings (1.26 g, 1.44 equiv), and the magnesium was activated as described above. A solution of chloromethyltrimethylsilane in THF (1.0 M, 45 mL, 45 mmol, 1.3 equiv)^59^ was passed through the packed column (approximately 2.8 mL free volume) at 100 °C at a flow rate of 0.1 mL min^–1^ (residence time ≈28 min). The outlet solution was mixed via T-piece with a flow of aldehyde in THF (0.8 M, 45 mL, 36 mmol, 1.0 equiv)^59^ pumping at 0.1 mL min^–1^ before passing through a 2 mL tubing reactor at 40 °C (residence time = 10 min). The output reaction mixture was quenched in flow by the addition of sulfuric acid (2.00 M, 110 mL, 221 mmol, 6.13 equiv)^59^ pumped at 0.25 mL min^–1^. The crude product mixture was collected at steady state for 450 min (theoretical yield of 36 mmol). The mixture was transferred to a separating funnel using DCM (3 × 5 mL) and washed with water (15 × 20 mL). The organic layer was then washed with brine (20 mL) before purification, as noted for each product below.

### Grignard Reagent Formation from ClCH_2_SiMe_3_ and Peterson Olefination (Scaled-up General Procedure 1)

An Omnifit 15 × 150 mm glass column reactor was charged with magnesium turnings (12.6 g, 1.44 equiv), and the magnesium was activated as described above. A solution of chloromethyltrimethylsilane in THF (1.00 M, 450 mL, 450 mmol, 1.25 equiv)^59^ was passed through the packed column (approximately 28 mL free volume) at 100 °C at a flow rate of 0.1 mL min^–1^ (residence time ≈ 280 min). The outlet solution was mixed via T-piece with a flow of aldehyde in THF (0.8 M, 450 mL, 360 mmol, 1 equiv)^59^ pumping at 0.1 mL min^–1^ before passing through a 2 mL tubing reactor at 40 °C (residence time = 10 min). The output reaction mixture was quenched in flow by the addition of sulfuric acid (2.00 M, 1100 mL, 2210 mmol, 6.13 equiv)^59^ pumped at 0.245 mL min^–1^. The crude product mixture was collected at a steady state for 4500 min. The mixture was transferred using DCM (3 × 20 mL) and washed with water (15 × 100 mL). The organic layer was then washed with brine (100 mL) before purification, as noted for each product below.

#### 2-Vinylthiophene (**3**)

Using scaled-up general procedure 1 and purification by washing with water (15 × 100 mL) and distillation (90 °C at 25 mbar), the title product was afforded as a pale yellow oil (36.88 g, 93%); ^1^H NMR (400 MHz, CDCl_3_) δ ppm 7.16–7.06 (m, 1H), 7.01–6.89 (m, 2H), 6.79 (dd, *J=* 17.3, 10.8 Hz, 1H), 5.55 (d, *J* = 17.3 Hz, 1H), 5.12 (d, *J* = 10.8 Hz, 1H); ^13^C NMR (101 MHz, CDCl_3_) δ ppm 143.1, 129.9, 127.4, 125.8, 124.4, 113.3; GCMS *t*_R_ = 5.8 min, *m*/*z* 110 (M^+^, 100%), 109 ([M-H]^+^, 45%), 84 (23%), 75 (1%). Spectral data are in accordance with previously published data.^60,61^

#### 2-Vinylfuran (**12**)

Using general procedure 1 and purification by washing with water (10 × 40 mL) followed by distillation (90 °C at 25 mbar), the title compound was afforded as a pale yellow mixture with THF and byproduct TMS (by NMR) (2.91 g, 43%—calculated from NMR to take account of the nonremovable THF and TMS in molar ratio 48:6:46 (product:THF:Me_3_SiOH)); ^1^H NMR (400 MHz, CDCl_3_) δ ppm 7.36 (d, *J* = 1.7 Hz, 1H), 6.51 (dd, *J* = 17.5, 11.3 Hz, 1H), 6.37 (dd, *J* = 3.3, 1.7 Hz, 1H), 6.26 (d, *J* = 3.3 Hz, 1H), 5.66 (dd, *J* = 17.5, 1.3 Hz, 1H), 5.16 (dd, 1H, *J* = 11.3, 1.3 Hz); ^13^C NMR (101 MHz, CDCl_3_) δ ppm 153.0, 142.0, 125.0, 112.2, 111.2, 107.9. Spectral data are in accordance with previously published data.^62^

#### 3-Methylstyrene (**13**)

Using general procedure 1 followed by washing with water (10 × 40 mL) and distillation (90 °C @ 10 mmHg), the title compound was afforded as pale yellow liquid (3.53 g, 83%); ^1^H NMR (400 MHz, CDCl_3_) δ ppm 7.17–7.28 (m, 3 H), 7.02–7.10 (m, 1H), 6.68 (dd, *J* = 17.7, 10.8 Hz, 1H), 5.72 (dd, *J* = 17.7, 1.0 Hz, 1H), 5.21 (dd, *J* = 10.8, 1.0 Hz, 1H), 2.34 (s, 3H); ^13^C NMR (101 MHz, CDCl_3_) δ ppm 138.1, 137.6, 137.0, 128.6, 128.5, 127.0, 123.4, 113.6, 21.4; GCMS *t*_R_ = 7.8 min, *m*/*z* 118 (M^+^, 100%), 117 ([M-H]^+^, 99%), 115 (42%), 91 (35%). Spectral data are in accordance with previously published data.^63,64^

#### Methyl 4-Vinylbenzoate (**14**)

Adapted from general procedure 1 by using different volumes: chloromethyltrimethylsilane in THF (25 mL, 25 mmol), 4-formylmethylbenzoate in THF (25 mL, 25 mmol), and aqueous H_2_SO_4_ (2 M, 61.25 mL, 125 mmol). Extraction with DCM (3 × 5 mL) and drying *in vacuo* were followed with purification by silica flash column chromatography (20% ethyl acetate in hexanes), which afforded the title compound as a white solid (3.16 g, 78%); mp 61–62 °C; ^1^H NMR (400 MHz, CDCl_3_) δ ppm ^1^H NMR (CHCl_3_, 400 MHz) δ 7.99 (d, *J* = 8.4 Hz, “H), 7.45 (d, *J* = 8.4 Hz, 2H), 6.74 (dd, *J* = 10.9, 17.6 Hz, 1H), 5.85 (d, *J* = 17.6 Hz, 1H), 5.37 (d, *J* = 10.9 Hz, 1H), 3.91 (s, 3H); ^13^C NMR (101 MHz, CDCl_3_) δ ppm 166.9, 141.9, 136.0, 129.9, 129.3, 126.1, 116.5, 52.1; GCMS *t*_R_ = 12.8 min, *m/z* 162 (M^+^, 52%), 131 (100%), 103 (32%), 77 (25%). Spectral data are in accordance with previously published data.^65,66^

### Grignard Reagent Formation from ClCH_2_SiPhMe_2_ and Peterson Olefination (General Procedure 2)

An Omnifit 15 × 150 mm glass column reactor was charged with magnesium turnings (1.26 g, 52.5 mmol 2.33 equiv), and the magnesium was activated as described above. A solution of (chloromethyl)dimethylphenylsilane in THF (1.00 M, 22.5 mL, 22.5 mmol, 1.25 equiv)^59^ was passed through the packed column (approximately 2.8 mL free volume) at 100 °C at a flow rate of 0.1 mL min^–1^ (residence time ≈28 min). The outlet solution was mixed with a flow of aldehyde in THF (0.80 M, 23 mL, 18 mmol, 1 equiv)^59^ pumping at 0.1 mL min^–1^ before passing through a 2 mL tubing reactor at 80 °C (residence time = 10 min). This reaction mixture was quenched in flow by the addition of aqueous sulfuric acid (2.00 M, 55 mL, 110.5 mmol, 6.13 equiv).^59^ The crude product mixture was collected at a steady state for 225 min. The mixture was then extracted with DCM (5 × 10 mL), dried using MgSO_4_ (5 g), and subsequently dried *in vacuo*.

#### 2-(Dimethylphenylsilyl)-1-(1′-methyl-1H-imidazol-2′-yl)ethan-1-ol (**21**)

Using general procedure 2, the title compound was isolated as an orange solid (3.56 g, 76%); mp 67 °C; IR: *v*_max_/cm^–1^ 3115 (OH br), 3065 (C = C–H), 2950 (CH), 1247, 824 (Si–C); ^1^H NMR (500 MHz, CDCl_3_) δ ppm 7.41–7.47 (m, 2H, *meta-*PhH), 7.28–7.34 (m, 3H, *ortho-, para-*PhH), 6.78 (br s, 1H, H-4′), 6.62 (br s, 1H, H-5′), 4.80 (t, *J* = 7.8 Hz, 1H, H-1), 3.46 (s, 3H, N–CH_3_), 1.56 (dd, *J* = 7.8, 1.6 Hz, 2H, H-2), 0.23 (s, 3H, Si–CH_3_), 0.22 (s, 3H, Si–CH_3_); ^13^C NMR (126 MHz, CDCl_3_) δ ppm, ^13^C NMR (CHCl_3_, 126 MHz) δ 150.4 (C-2′), 138.6 (*ispo-*PhC), 133.5 (*meta*-PhC), 128.8 (*para*-PhC), 127.6 (*ortho*-PhC), 126.2 (C-4′), 121.2 (C-5′), 64.3 (C-1), 32.6 (N-CH_3_), 24.2 (C-2), −2.7 (Si(CH_3_)_2_); HRMS (ESI) found: [M+H^+^], 199.1262, C_9_H_18_N_2_OSi requires [M + H]^+^, 199.1267.

#### 2-(Dimethylphenylsilyl)-1-(pyridine-3′-yl)ethan-1-ol (**22**)

Using general procedure 2, the title compound was isolated as a colorless oil (3.47 g, 74%); IR: *v*_max_/cm^–1^ 3369 (br, O–H), 3068 (C = C–H), 2954 (C–H), 1429 (C–N), 1246 and 821 (Si–C); ^1^H NMR (400 MHz, CDCl_3_) δ ppm 8.47 (d, *J* = 4.8 Hz, 1H, H-2′), 7.59 (td, *J* = 8.0, 1.7 Hz, 1H, H-5′), 7.49–7.57 (m, 2H, *meta-*PhH), 7.30–7.39 (m, 3H, *ortho, para-*PhH), 7.20 (d, *J* = 8.0 Hz, 1H, H-4′), 7.08–7.16 (m, 1H, H-6′), 4.90 (dd, *J* = 9.0, 5.3 Hz, 1H, H-1), 1.48 (dd, *J* = 14.9, 5.3 Hz, 1H, H-2), 1.38 (m, 1H, H-2), 0.37 (s, 3H, Si–CH_3_), 0.31 (s, 3H, Si–CH_3_); ^13^C NMR (101 MHz, CDCl_3_) δ ppm 164.1 (C-3′), 148.1 (C-2′), 139.2 (*ipso*-PhC), 136.5 (C-5′), 133.5 (*meta-*PhC), 128.7 (*para*-PhC), 127.6 (*ortho*-PhC), 122.0 (C-6′), 120.0 (C-4′), 71.2 (C-1), 26.8 (C-2), −2.1 (Si-CH_3_), −2.4 (Si-CH_3_); HRMS (ESI) found: [M + H]^+^, 258.1309. C_15_H_19_ONSi requires [M + H]^+^, 258.1311.

#### 1-Methyl-2-vinylimidazole (**15**)

Silyl alcohol **21** in THF (0.40 M, 5.9 mL, 2.4 mmol, 1.0 equiv)^59^ was combined with a solution of H_2_SO_4_ in water (6.0 M, 5.9 mL, 35 mmol, 15 equiv),^59^ each at a flow rate of 0.1 mL min^–1^ before passing through a 10 mL tubing reactor at 100 °C. The reaction mixture was collected at steady state for 29.5 min before being extracted with DCM (5 × 10 mL), dried using MgSO_4_ (5 g), and subsequently concentrated *in vacuo* to yield the title compound as an orange solid (0.22 g, 87%); mp 44–46 °C; ^1^H NMR (400 MHz, CDCl_3_) 7.02 (br s, 1H), 6.84 (br s, 1H), 6.60 (ddt, *J* = 17.3, 11.3, 0.6 Hz, 1H), 6.14 (ddd, *J* = 17.3, 1.2, 1.0 Hz, 1H), 5.42 (dm, *J* = 11.3 Hz, 1H), 3.66 (d, *J* = 0.9 Hz, 3H); ^13^C NMR (101 MHz, CDCl_3_) δ ppm 145.4, 128.4, 122.5, 121.4, 118.0, 32.7. Spectral data are in accordance with previously published data.^67^

#### 3-Vinylpyridine (**16**)

Silyl alcohol **22** in THF (0.4 M, 5.9 mL, 2.4 mmol, 1.0 equiv)^59^ was combined with a solution of *p*-toluene sulfonic acid in THF (2.0 M, 5.9 mL, 12 mmol, 5.0 equiv),^59^ each at a flow rate of 0.2 mL min^–1^ before passing through a 10 mL tubing reactor at 100 °C. The reaction mixture was collected at steady state for 15 min, and the solution was then analyzed by quantitative NMR using a benzyl benzoate internal standard and the peak for the product at 6.00 ppm (d, 1H) [N.B. peaks for the vinyl group were shifted compared to those reported in the literature, and this is thought to be the result of the NMR sample being a solution in CDCl_3_ and THF^68^] (0.40 mmol, 33% by quantitative NMR).

#### 2-(Dimethylphenylsilyl)-1-(thiophen-2′-yl)ethan-1-ol (**23**)

Using general procedure 2, but with the following volumes: chloromethyldimethylphenylsilane in THF (12.9 mL, 12.9 mmol) and 2-thiophenecarboxaldehyde in THF (12.9 mL, 10.3 mmol) and quenching with water (31.5 mL) instead of H_2_SO_4_, followed by extraction with DCM (5 × 5 mL) and purification by silica flash column chromatography (20% ethyl acetate in hexanes) afforded the title compound as a yellow oil (0.64 g, 24%); IR: 3345 (br, O–H), 3069 (C = C–H), 2955 (C–H), 1248 and 826 (Si–C); ^1^H NMR (400 MHz, CDCl_3_) δ ppm 7.47–7.54 (m, 2H, *meta-*PhH), 7.32–7.38 (m, 3H, *ortho-, para-*PhH), 7.20 (dd, *J* = 5.2, 1.2 Hz, 1H, H-5′), 6.90 (dd, *J* = 5.2, 3.7 Hz, 1H, H-4′), 6.81–6.89 (m, 1H, H-3′), 5.05 (t, *J* = 7.4 Hz, 1H, H-1), 1.89 (br s, 1H, OH), 1.58 (dd, *J* = 14.2, 8.0 Hz, 1H, H-2a), 1.50 (dd, *J* = 14.2, 6.6 Hz, 1H, H-2b), 0.251 (s, 3H, Si–CH_3_), 0.247 (s, 3H, Si–CH_3_); ^13^C NMR (101 MHz, CDCl_3_) δ ppm 150.9 (C-2′), 138.6 (*ipso-*PhC), 133.6 (*meta-*PhC), 129.0 (*para-*PhC), 127.8 (*ortho-*PhC), 126.4 (C-4′), 124.5 (C-5′), 123.4 (C-3′), 68.1 (C-1), 28.1 (C-2), −2.5 (Si-CH_3_), −2.8 (Si-CH_3_); HRMS (ESI) found: [M-H]^+^, 263.0944. C_14_H_18_OSSi requires [M + H]^+^ = 263.0926.

#### 1-(Pyridine-3′-yl)-2-(trimethylsilyl)ethan-1-ol (**24**)

An Omnifit 15 × 150 mm glass column reactor was charged with magnesium turnings (1.26 g, 52.5 mmol, 3.50 equiv, approximately 2.8 mL free volume), and the magnesium was activated as described above. A solution of chloromethyltrimethylsilane in THF (1.0 M, 15 mL, 15 mmol, 1.0 equiv)^59^ was passed through at 100 °C at a flow rate of 0.1 mL min^–1^ (residence time ≈28 min). The outlet solution of the Grignard reagent was collected at steady state for 150 min into a flask under nitrogen. A solution of 3-pyridinecarboxaldehyde in THF (0.80 M, 15 mL, 12 mmol, 0.80 equiv) was added to the flask at 40 °C, and the mixture was stirred for 10 min. The reaction was then quenched using excess water (approximately 10 mL) before being basified using saturated aqueous sodium carbonate (approximately 3 mL) and then extracted with DCM (5 × 5 mL) and dried *in vacuo* to yield hydroxysilane **6c** as a pale yellow oil (1.25 g, 53%); IR: *v*_max_/cm^–1^ 3226 (br, O–H), 2952 (C–H), 1423 (C–N), 1246 and 836; ^1^H NMR (400 MHz, CDCl_3_) δ ppm 8.46 (br s, 1H, H-2′), 8.41 (br d, *J* = 4.2 Hz, 1H, H-6′), 7.70 (br d, *J* = 7.9 Hz, 1H, H-4′), 7.23 (dd, *J* = 7.9, 4.2 Hz, 1H, H-5′), 4.86 (br t, *J* = 7.9 Hz, 1H, H-1), 3.21 (br s, 1H, OH), 1.26 (dd, *J* = 14.5, 7.8 Hz, 1H, H-2a), 1.14 (dd, *J* = 14.5, 8.0 Hz, 1H, H-2b), −0.07 (s, 9H, Si(CH_3_)_3_); ^13^C NMR (101 MHz, CDCl_3_) δ ppm 148.6 (C-6′), 147.5 (C-2′), 142.1 (C-3′), 133.5 (C-4′), 123.5 (C-5′), 70.2 (C-1), 28.4 (C-2), −1.1 (Si(CH_3_)_3_); HRMS (ESI) found: [M-H]^−^, 194.0999. C_10_H_17_NOSi requires [M-H]^−^, 194.1007.

## Data Availability

The data underlying this study are available in the published article and its Supporting Information.

## Supporting Information Available

Additional experimental details for the Wittig reaction. Flow reactor setup screenshot and photograph of magnesium-filled column reactor, example GCMS chromatograms and spectra from reaction optimization work. ^1^H, ^13^C, and some two-dimensional NMR of all compounds (PDF)

## Footnotes

a A 1:1 molar mixture of thiophene-2-carboxaldehyde and 2-vinylthiophene was made and analyzed by GCMS, which showed a peak area ratio of 100:97, respectively.

## References

[ref1] Scheirs J.; Priddy D. B. Modern Styrenic Polymers: Polystyrenes and Styrenic Copolymers; Wiley, 2003.

[ref2] Yoshida E.; Kunugi S. Micelle formation of poly(vinyl phenol)-block-polystyrene by α,ω-diamines. J. Polym. Sci., Part A:Polym. Chem. 2002, 40, 3063–3067. 10.1002/pola.10399.

[ref3] Maharramov A. M.; Bayramov M. R.; Agayeva M. A.; Mehdiyeva G. M.; Mamedov I. G. Alkenylphenols: preparation, transformations and applications. Russ. Chem. Rev. 2015, 84, 1258 10.1070/RCR4437.

[ref4] Kennemur J. G. Poly(vinylpyridine) Segments in Block Copolymers: Synthesis, Self-Assembly, and Versatility. Macromolecules 2019, 52, 1354–1370. 10.1021/acs.macromol.8b01661.

[ref5] Mavronasou K.; Zamboulis A.; Klonos P.; Kyritsis A.; Bikiaris D. N.; Papadakis R.; Deligkiozi I. Poly(vinyl pyridine) and Its Quaternized Derivatives: Understanding Their Solvation and Solid State Properties. Polymers 2022, 14, 804 10.3390/polym14040804. 35215717 PMC8962976

[ref6] Xue Y.; Xiao H. Antibacterial/Antiviral Property and Mechanism of Dual-Functional Quaternized Pyridinium-type Copolymer. Polymers 2015, 7, 2290–2303. 10.3390/polym7111514.

[ref7] Kandasamy G.; Danilovtseva E. N.; Annenkov V. V.; Krishnan U. M. Poly(1-vinylimidazole) polyplexes as novel therapeutic gene carriers for lung cancer therapy. Beilstein J. Nanotechnol. 2020, 11, 354–369. 10.3762/bjnano.11.26. 32190532 PMC7061483

[ref8] Wang B.; Liu H.-J.; Chen Y. A biocompatible poly(N-vinylimidazole)-dot with both strong luminescence and good catalytic activity. RSC Adv. 2016, 6, 2141–2148. 10.1039/C5RA20640E.

[ref9] Pinaud J.; Vignolle J.; Gnanou Y.; Taton D. Poly(N-heterocyclic-carbene)s and their CO2 Adducts as Recyclable Polymer-Supported Organocatalysts for Benzoin Condensation and Transesterification Reactions. Macromolecules 2011, 44, 1900–1908. 10.1021/ma1024285.

[ref10] Fan B.; Wan J.; McKay A.; Qu Z.; Thang S. H. Facile synthesis of well-controlled poly(1-vinyl imidazole) by the RAFT process. Polym. Chem. 2020, 11, 5649–5658. 10.1039/D0PY00985G.

[ref11] Höhne S.; Seifert A.; Friedrich M.; Holze R.; Spange S. Cationic Polymerization of 2-Vinylthiophene by Chloroarylmethanes as Surface Initiators on Silica and Consecutive Hydride Abstraction by Acceptors. Macromol. Chem. Phys. 2004, 205, 1667–1676. 10.1002/macp.200400098.

[ref12] Wang T.; Wu C.; Cui D. Highly syndioselective coordination (co)polymerization of vinyl heteroaromatic monomers using rare-earth-metal complexes. Polym. Chem. 2020, 11, 7650–7655. 10.1039/D0PY01447H.

[ref13] Zhou Y.; Engl O. D.; Bandar J. S.; Chant E. D.; Buchwald S. L. CuH-Catalyzed Asymmetric Hydroamidation of Vinylarenes. Angew. Chem., Int. Ed. 2018, 57, 6672–6675. 10.1002/anie.201802797. PMC5992098 29660768

[ref14] Ma S.; Hartwig J. F. Progression of Hydroamination Catalyzed by Late Transition-Metal Complexes from Activated to Unactivated Alkenes. Acc. Chem. Res. 2023, 56, 1565–1577. 10.1021/acs.accounts.3c00141. 37272995 PMC11620761

[ref15] Tran H. N.; Stanley L. M. Nickel-Catalyzed Enantioselective Hydroboration of Vinylarenes. Org. Lett. 2022, 24, 395–399. 10.1021/acs.orglett.1c04073. 34905379

[ref16] Wang C.; Teo W. J.; Ge S. Cobalt-Catalyzed Regiodivergent Hydrosilylation of Vinylarenes and Aliphatic Alkenes: Ligand- and Silane-Dependent Regioselectivities. ACS Catal. 2017, 7, 855–863. 10.1021/acscatal.6b02518.

[ref17] Straathof N. J. W.; Cramer S. E.; Hessel V.; Noël T. Practical Photocatalytic Trifluoromethylation and Hydrotrifluoromethylation of Styrenes in Batch and Flow. Angew. Chem., Int. Ed. 2016, 55, 15549–15553. 10.1002/anie.201608297. 27862770

[ref18] Bao X.; Li J.; Jiang W.; Huo C. Radical-Mediated Difunctionalization of Styrenes. Synthesis 2019, 51, 4507–4530. 10.1055/s-0039-1690987.

[ref19] Giron Rodriguez C. A.; Joensen B. Ó.; Moss A. B.; Larrazábal G. O.; Whelligan D. K.; Seger B.; Varcoe J. R.; Willson T. R. Influence of Headgroups in Ethylene-Tetrafluoroethylene-Based Radiation-Grafted Anion Exchange Membranes for CO2 Electrolysis. ACS Sustainable Chem. Eng. 2023, 11, 1508–1517. 10.1021/acssuschemeng.2c06205. 36743393 PMC9890565

[ref20] Ponce-González J.; Varcoe J. R.; Whelligan D. K. Commercial Monomer Availability Leading to Missed Opportunities? Anion-Exchange Membranes Made from meta-Vinylbenzyl Chloride Exhibit an Alkali Stability Enhancement. ACS Appl. Energy Mater. 2018, 1, 1883–1887. 10.1021/acsaem.8b00438.

[ref21] Ponce-Gonzalez J.; Ouachan I.; Varcoe J. R.; Whelligan D. K. Radiation-induced grafting of a butyl-spacer styrenic monomer onto ETFE: the synthesis of the most alkali stable radiation-grafted anion-exchange membrane to date. J. Mater. Chem. A 2018, 6, 823–827. 10.1039/C7TA10222D.

[ref22] This research will be published in due course.

[ref23] Wang M.; Preston N.; Xu N.; Wei Y.; Liu Y.; Qiao J. Promoter Effects of Functional Groups of Hydroxide-Conductive Membranes on Advanced CO2 Electroreduction to Formate. ACS Appl. Mater. Interfaces 2019, 11, 6881–6889. 10.1021/acsami.8b11845. 30676728

[ref24] Plutschack M. B.; Pieber B.; Gilmore K.; Seeberger P. H. The Hitchhiker’s Guide to Flow Chemistry. Chem. Rev. 2017, 117, 11796–11893. 10.1021/acs.chemrev.7b00183. 28570059

[ref25] Morodo R.; Bianchi P.; Monbaliu J.-C. M. Continuous Flow Organophosphorus Chemistry. Eur. J. Org. Chem. 2020, 2020, 5236–5277. 10.1002/ejoc.202000430.

[ref26] Okamoto H.; Takahashi H.; Takane T.; Nishiyama Y.; Kakiuchi K.; Gohda S.; Yamaji M. Convenient Phenacene Synthesis by Sequentially Performed Wittig Reaction and Mallory Photocyclization Using Continuous-Flow Techniques. Synthesis 2017, 49, 2949–2957. 10.1055/s-0036-1588775.

[ref27] Zhang X. M.; Bordwell F. G. Equilibrium acidities and homolytic bond dissociation energies of the acidic carbon-hydrogen bonds in P-substituted triphenylphosphonium cations. J. Am. Chem. Soc. 1994, 116, 968–972. 10.1021/ja00082a018.

[ref28] Pathare R. S.; Sharma S.; Gopal K.; Sawant D. M.; Pardasani R. T. Palladium-catalyzed convenient one-pot synthesis of multi-substituted 2-pyrones via transesterification and alkenylation of enynoates. Tetrahedron Lett. 2017, 58, 1387–1389. 10.1016/j.tetlet.2017.02.060.

[ref29] Hu N.; Jung H.; Zheng Y.; Lee J.; Zhang L.; Ullah Z.; Xie X.; Harms K.; Baik M.-H.; Meggers E. Catalytic Asymmetric Dearomatization by Visible-Light-Activated [2 + 2] Photocycloaddition. Angew. Chem., Int. Ed. 2018, 57, 6242–6246. 10.1002/anie.201802891. 29624849

[ref30] Wadekar M. N.; Jager W. F.; Sudhölter E. J. R.; Picken S. J. Synthesis of a Polymerizable Fluorosurfactant for the Construction of Stable Nanostructured Proton-Conducting Membranes. J. Org. Chem. 2010, 75, 6814–6819. 10.1021/jo101196w. 20845981

[ref31] Seferos D. S.; Blum A. S.; Kushmerick J. G.; Bazan G. C. Single-Molecule Charge-Transport Measurements that Reveal Technique-Dependent Perturbations. J. Am. Chem. Soc. 2006, 128, 11260–11267. 10.1021/ja062898j. 16925445

[ref32] Pepitone M. F.; Jernigan G. G.; Melinger J. S.; Kim O. K. Synthesis and Characterization of Donor–Acceptor Chromophores for Unidirectional Electron Transfer. Org. Lett. 2007, 9, 801–804. 10.1021/ol063000y. 17286407

[ref33] Hamlin T. A.; Lazarus G. M. L.; Kelly C. B.; Leadbeater N. E. A Continuous-Flow Approach to 3,3,3-Trifluoromethylpropenes: Bringing Together Grignard Addition, Peterson Elimination, Inline Extraction, and Solvent Switching. Org. Process Res. Dev. 2014, 18, 1253–1258. 10.1021/op500190j.

[ref34] Deng Q.; Shen R.; Zhao Z.; Yan M.; Zhang L. The continuous flow synthesis of 2,4,5-trifluorobenzoic acid via sequential Grignard exchange and carboxylation reactions using microreactors. Chem. Eng. J. 2015, 262, 1168–1174. 10.1016/j.cej.2014.10.066.

[ref35] von Keutz T.; Cantillo D.; Kappe C. O. Organomagnesium Based Flash Chemistry: Continuous Flow Generation and Utilization of Halomethylmagnesium Intermediates. Org. Lett. 2020, 22, 7537–7541. 10.1021/acs.orglett.0c02725. 32914630 PMC7586391

[ref36] Reimschuessel H. Notes: On the Continuous Preparation of Vinyl Grignard Reagents. J. Org. Chem. 1960, 25, 2256–2257. 10.1021/jo01082a628.

[ref37] Klokov B. A. Continuous and Batch Organomagnesium Synthesis of Ethyl-Substituted Silanes from Ethylchloride, Tetraethoxysilane, and Organotrichlorosilane for Production of Polyethylsiloxane Liquids. 2. Continuous One-Step Synthesis of Ethylethoxy- and Ethylchlorosilanes. Org. Process Res. Dev. 2001, 5, 234–240. 10.1021/op000100p.

[ref38] Huck L.; de la Hoz A.; Díaz-Ortiz A.; Alcázar J. Grignard Reagents on a Tab: Direct Magnesium Insertion under Flow Conditions. Org. Lett. 2017, 19, 3747–3750. 10.1021/acs.orglett.7b01590. 28657761

[ref39] Deng Y. C.; Wei X. J.; Wang X.; Sun Y. H.; Noel T. Iron-Catalyzed Cross-Coupling of Alkynyl and Styrenyl Chlorides with Alkyl Grignard Reagents in Batch and Flow. Chem. - Eur. J. 2019, 25, 14532–14535. 10.1002/chem.201904480. 31573119 PMC6900226

[ref40] Menges-Flanagan G.; Deitmann E.; Gossl L.; Hofmann C.; Lob P. Scalable Continuous Synthesis of Grignard Reagents from in Situ Activated Magnesium Metal. Org. Process Res. Dev. 2020, 24, 315–321. 10.1021/acs.oprd.9b00493.

[ref41] Grachev A. A.; Klochkov A. O.; Shiryaev V. I. Continuous synthesis of organomagnesium compounds. Russ. J. Appl. Chem. 2012, 85, 629–638. 10.1134/S1070427212040167.

[ref42] Goldbach M.; Danieli E.; Perlo J.; Kaptein B.; Litvinov V. M.; Blümich B.; Casanova F.; Duchateau A. L. L. Preparation of Grignard reagents from magnesium metal under continuous flow conditions and on-line monitoring by NMR spectroscopy. Tetrahedron Lett. 2016, 57, 122–125. 10.1016/j.tetlet.2015.11.077.

[ref43] Kopach M. E.; Roberts D. J.; Johnson M. D.; McClary Groh J.; Adler J. J.; Schafer J. P.; Kobierski M. E.; Trankle W. G. The continuous flow Barbier reaction: an improved environmental alternative to the Grignard reaction?. Green Chem. 2012, 14, 1524–1536. 10.1039/c2gc35050e.

[ref44] Braden T. M.; Johnson M. D.; Kopach M. E.; McClary Groh J.; Spencer R. D.; Lewis J.; Heller M. R.; Schafer J. P.; Adler J. J. Development of a Commercial Flow Barbier Process for a Pharmaceutical Intermediate. Org. Process Res. Dev. 2017, 21, 317–326. 10.1021/acs.oprd.6b00373.

[ref45] Wong S.-W.; Changi S. M.; Shields R.; Bell W.; McGarvey B.; Johnson M. D.; Sun W.-M.; Braden T. M.; Kopach M. E.; Spencer R. D.; Flanagan G.; Murray M. Operation Strategy Development for Grignard Reaction in a Continuous Stirred Tank Reactor. Org. Process Res. Dev. 2016, 20, 540–550. 10.1021/acs.oprd.5b00268.

[ref46] Pinho S. P.; Macedo E. A. Experimental measurement and modelling of KBr solubility in water, methanol, ethanol, and its binary mixed solvents at different temperatures. J. Chem. Thermodyn. 2002, 34, 337–360. 10.1006/jcht.2001.0856.

[ref47] The facilitation of the Wittig reaction in methanol by addition of diglyme was a serendipitous discovery since it was originally added for use as an internal standard during GCMS.

[ref48] Langer J.; Geitner R.; Görls H. Syntheses and Structures of Potassium Complexes Containing Bis(diphenylphosphanyl)methanide Anions. Eur. J. Inorg. Chem. 2014, 2014, 1413–1420. 10.1002/ejic.201301584.

[ref49] Shao Y.; Liu Z.; Huang P.; Liu B. A unified model of Grignard reagent formation. Phys. Chem. Chem. Phys. 2018, 20, 11100–11108. 10.1039/C8CP01031E. 29620768

[ref50] Garst J. F.; Easton Lawrence K.; Batlaw R.; Boone J. R.; Ungváry F. Magnesium bromide in Grignard reagent formation. Inorg. Chim. Acta 1994, 222, 365–375. 10.1016/0020-1693(94)03928-3.

[ref51] Wingard L. A.; Guzmán P. E.; Sabatini J. J. A Chlorine Gas-Free Synthesis of Dichloroglyoxime. Org. Process Res. Dev. 2016, 20, 1686–1688. 10.1021/acs.oprd.6b00252.

[ref52] Fauvel A.; Deleuze H.; Landais Y. New Polymer-Supported Organosilicon Reagents. Eur. J. Org. Chem. 2005, 2005, 3900–3910. 10.1002/ejoc.200500252.

[ref53] Dilien H.; Marin L.; Botek E.; Champagne B.; Lemaur V.; Beljonne D.; Lazzaroni R.; Cleij T. J.; Maes W.; Lutsen L.; Vanderzande D.; Adriaensens P. J. Fingerprints for Structural Defects in Poly(thienylene vinylene) (PTV): A Joint Theoretical-Experimental NMR Study on Model Molecules. J. Phys. Chem. B 2011, 115, 12040–12050. 10.1021/jp206663v. 21894975

[ref54] Britten T. K.; McLaughlin M. G. Bronsted Acid Catalyzed Peterson Olefinations. J. Org. Chem. 2020, 85, 301–305. 10.1021/acs.joc.9b02489. 31775003

[ref55] Malet-Sanz L.; Susanne F. Continuous Flow Synthesis. A Pharma Perspective. J. Med. Chem. 2012, 55, 4062–4098. 10.1021/jm2006029. 22283413

[ref56] Bannon R.; Smyth M.; Moody T. S.; Wharry S.; Roth P. M. C.; Gauron G.; Baumann M. Continuous Flow Synthesis of β-Aminoketones as Masked Vinyl Ketone Equivalents. Chem. - Eur. J. 2025, 31, e202500014 10.1002/chem.202500014. 39906938

[ref57] Ager D. J. Organic Reactions; Paquette L. A., Ed.; John Wiley & Sons, 1990; Vol. 38, p 1.

[ref58] Williams D. B. G.; Lawton M. Drying of Organic Solvents: Quantitative Evaluation of the Efficiency of Several Desiccants. J. Org. Chem. 2010, 75, 8351–8354. 10.1021/jo101589h. 20945830

[ref59] Solutions were prepared in larger amounts but those volumes and mmol given correspond to the time for which the product stream was collected.

[ref60] Shi X.; ting Du T.; Zhang Z.; Liu X.; Yang Y.; Xue N.; Jiao X.; Chen X.; Xie P. (+)-Isocryptotanshinone derivatives and its simplified analogs as STAT3 signaling pathway inhibitors. Bioorg. Chem. 2022, 127, 106015 10.1016/j.bioorg.2022.106015. 35849894

[ref61] Chowdhury D.; Goswami S.; Krishna G. R.; Mukherjee A. Transfer semi-hydrogenation of terminal alkynes with a well-defined iron complex. Dalton Trans. 2024, 53, 3484–3489. 10.1039/D3DT03248E. 38312066

[ref62] Shiramizu M.; Toste F. D. Deoxygenation of biomass-derived feedstocks: Oxorhenium-catalyzed deoxydehydration of sugars and sugar alcohols. Angew. Chem., Int. Ed. 2012, 51, 8082–8086. 10.1002/anie.201203877. 22764085

[ref63] Yadav S.; Dutta I.; Saha S.; Das S.; Pati S. K.; Choudhury J.; Bera J. K. An Annelated Mesoionic Carbene (MIC) Based Ru(II) Catalyst for Chemo- And Stereoselective Semihydrogenation of Internal and Terminal Alkynes. Organometallics 2020, 39, 3212–3223. 10.1021/acs.organomet.0c00413.

[ref64] Liu Q.; Yang L.; Yao C.; Geng J.; Wu Y.; Hu X. Controlling the Lewis Acidity and Polymerizing Effectively Prevent Frustrated Lewis Pairs from Deactivation in the Hydrogenation of Terminal Alkynes. Org. Lett. 2021, 23, 3685–3690. 10.1021/acs.orglett.1c01073. 33877853

[ref65] Golfmann M.; Glagow L.; Giakoumidakis A.; Golz C.; Walker J. C. L. Organophotocatalytic 2 + 2 Cycloaddition of Electron-Deficient Styrenes**. Chem. - Eur. J. 2023, 29, e202202373 10.1002/chem.202202373. 36282627 PMC10100360

[ref66] Movahhed S.; Westphal J.; Dindaroğlu M.; Falk A.; Schmalz H.-G. Low-Pressure Cobalt-Catalyzed Enantioselective Hydrovinylation of Vinylarenes. Chem. - Eur. J. 2016, 22, 7381–7384. 10.1002/chem.201601283. 26998912

[ref67] Waser J.; Gaspar B.; Nambu H.; Carreira E. M. Hydrazines and azides via the metal-catalyzed hydrohydrazination and hydroazidation of olefins. J. Am. Chem. Soc. 2006, 128, 11693–11712. 10.1021/ja062355+. 16939295

[ref68] Gallardo-Rosas D.; Guevara-Vela J. M.; Rocha-Rinza T.; Toscano R. A.; López-Cortés J. G.; Ortega-Alfaro M. C. Structure and isomerization behavior relationships of new push–pull azo-pyrrole photoswitches. Org. Biomol. Chem. 2024, 22, 4123–4134. 10.1039/D4OB00417E. 38700442

