# Supplementary material for: Continuous-Flow Synthesis of Primary Vinylarenes via Inline Grignard Reagent Formation and Peterson Olefination
Source: ACS Omega. 2025 Apr 25;10(17):17887–97. doi: 10.1021/acsomega.5c00823 (PMC12060042; doi:10.1021/acsomega.5c00823)
Supplement: Supplementary file 1 — ao5c00823_si_001.pdf [file ao5c00823_si_001.pdf]

Continuous-Flow Synthesis of Primary Vinylarenes via Inline Grignard Reagent Formation and Peterson Olefination

Matthew J. Burrell, John R. Varcoe, Daniel K. Whelligan*

School of Chemistry and Chemical Engineering, University of Surrey, Guildford, GU2 7XH, U. K.

* d.whelligan@surrey.ac.uk

Supplementary Information

Supplementary Information 1
- General Experimental 2
- Wittig Reaction 2
- Grignard Reagent Formation and Peterson Olefination 4
  - Flow reactor setup 4
  - GCMS Data 5
- NMR Spectra 7
  - 2-Vinylthiophene (3) 7
  - 2-Vinylfuran (12) (product : THF : Me_3SiOH = 48 : 6 : 46 mol%) 9
  - 3-Methylstyrene (13).... 11
  - Methyl 4-vinylbenzoate (14) 13
  - 2-(Dimethylphenylsilyl)-1-(1-methyl-1H-imidazol-2-yl)ethan-1-ol (21) 15
  - 2-(Dimethylphenylsilyl)-1-(pyridine-3-yl)ethan-1-ol (22) 20
  - 1-Methyl-2-vinylimidazole (15) 25
  - 3-Vinylpyridine (16, present in THF, benzyl benzoate internal standard): 50 mg solution in THF + 2.86 mg internal standard in $CDCl_3$ 27
  - 2-(Dimethylphenylsilyl)-1-(thiophen-2-yl)ethan-1-ol (23).... 28
  - 1-(Pyridine-3-yl)-2-(trimethylsilyl)ethan-1-ol (24).... 33

References 37

General Experimental

All reactants were purchased from commercial sources and used without purification unless otherwise stated. Anhydrous THF was inhibitor-free and purchased from Sigma Aldrich in bottles with septa attached or was prepared by passage through an activated alumina column, using a Pure Solv™ Micro Solvent Purification System, and was stored over activated molecular sieves (3 Å, 8 to 12 mesh). Standard Schlenk line techniques were used for the preparation of solutions ready for plumbing to the flow reactor.

NMR spectra were obtained on a Bruker 500 MHz or 400 MHz spectrometer. ^{1}H-NMR spectra were referenced either to TMS at 0 ppm or to residual (partially) protic solvent: 7.26 ppm for $CHCl_3$. ^{13}C-NMR spectra were recorded at 126 MHz or 101 MHz. They were referenced to $CDCl_3$ at 77.0 ppm.

GCMS spectra were recorded on one of two instruments: *Method 1:* an Agilent Technologies 7890A GC system connected to an Agilent Technologies 5975C inert XL EI/CI mass selective detector (MSD) operating in electron impact (EI) mode and the conditions were as follows: inj. vol. 2 µL, inj. temp. 280 °C, column Agilent HP-5MS (30 m × 0.25 mm), carrier gas (H_2) flow 1 ml min^{-1}, oven temperature gradient 0 – 3 min, 50 °C; 3-23 min, 50 – 250 °C (10 °C/min), 23-25 min, 250 °C. *Method 2:* an Agilent Technologies 8890 GC system connected to an Agilent Technologies 5977B MSD operating in EI mode and the conditions were as follows: inj. vol. 1 µL, inj. temp. 280 °C, column Agilent HP-5MS (30 m × 0.25 mm), carrier gas (H_2) flow 2 ml min^{-1}, oven temperature gradient 0 – 3 min, 50 °C; 3-11 min, 50 – 130 °C (10 °C/min), 11-17 min, 130 - 250 °C (20 °C/min).

Agilent MassHunter Quantitative Analysis (version 10.1) was used to generate extracted ion count (EIC) chromatograms and integrate peaks to generate the GCMS peak area ratios given in Table 1 of the main text.

Wittig Reaction

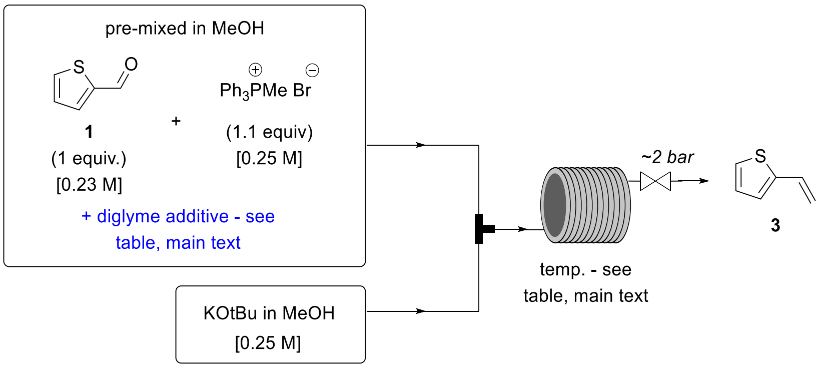

Scheme S1. Attempts at continuous flow Wittig reaction

Representative procedure (main text, Table 1, entry 4): A methanolic solution (0.75 ml)* of thiophene-2-carboxaldehyde (**1**) (0.23 M, 0.17 mmol, 1.00 equiv.)*, methyltriphenylphosphonium bromide (0.25 M, 0.19 mmol, 1.10 equiv.)* and diglyme (0.28 M, 0.21 mmol, 1.2 equiv.)* was pumped at 0.5 mL min^{-1} into a T-connector along with a solution of potassium *tert*-butoxide (0.25 M, 0.75 mL, 0.19 mmol, 1.1 equiv.)* at at 0.5 mL min^{-1} before the combined streams entered a 10 mL tubing reactor at 50 °C (residence time = 10 min). After passing through an adjustable back pressure regulator maintaining ~2 bar, the output reaction mixture (1.5 ml) was collected at steady state for analysis by GCMS Method 1 (results shown in Table 1 of the main text).

2-Vinylthiophene GCMS t_R = 6.9 min, *m/z* 110 (M^+, 100%), 109 ($[M-H]^+$, 42%), 95 (2%), 84 (22%), 66 (24%); peak *m/z* 110 used for EIC and integration; this data agrees with that given in the literature.[1]

* Solutions were prepared in larger amounts but those volumes and mmol given correspond to the time for which the product stream was collected.

Diglyme GCMS t_R = 8.0 min, *m/z* 110 (2%), 102 (1%), 89 ($MeO(CH_2)_2OCH_2^+$, 22%), 87 (2%), 59 (($MeO(CH_2)_2^+$, 100%), 58 (45%); peak *m/z* 89 used for EIC and integration; this data agrees with that given in the literature.[2]

Thiophene-2-carboxaldehyde GCMS t_R = 9.0 min, *m/z* 112 (M^+, 90%), 111 ($[M-H]^+$, 100%), 83 (11%), 81 (5%), 69 (3%), 58 (9%), 57 (9%); peak *m/z* 111 used for EIC and integration; this data agrees with that given in the literature.[3]

Methyltriphenylphosphonium bromide GCMS t_R = 21.9 min, *m/z* 277 (M^+, 0.3%), 215 (100%), 201 (64%), 183 (5%), 152 (6%), 139 (8%), 91 (5%), 77 (Ph^+, 21%), 51 (9%); this data partially agrees with that given in the literature (peaks 277, 201, 183, 152).[4]

Triphenylphosphine oxide GCMS t_R = 23.3 min, *m/z* 277 ($[M-H]^+$, 24%), 262 ($[M-O]^+$, 100%), 215 (44%), 201 (32%), 183 (97%), 152 (18%), 108 (31%), 77 (22%); this data agrees with that given in the literature.[5]

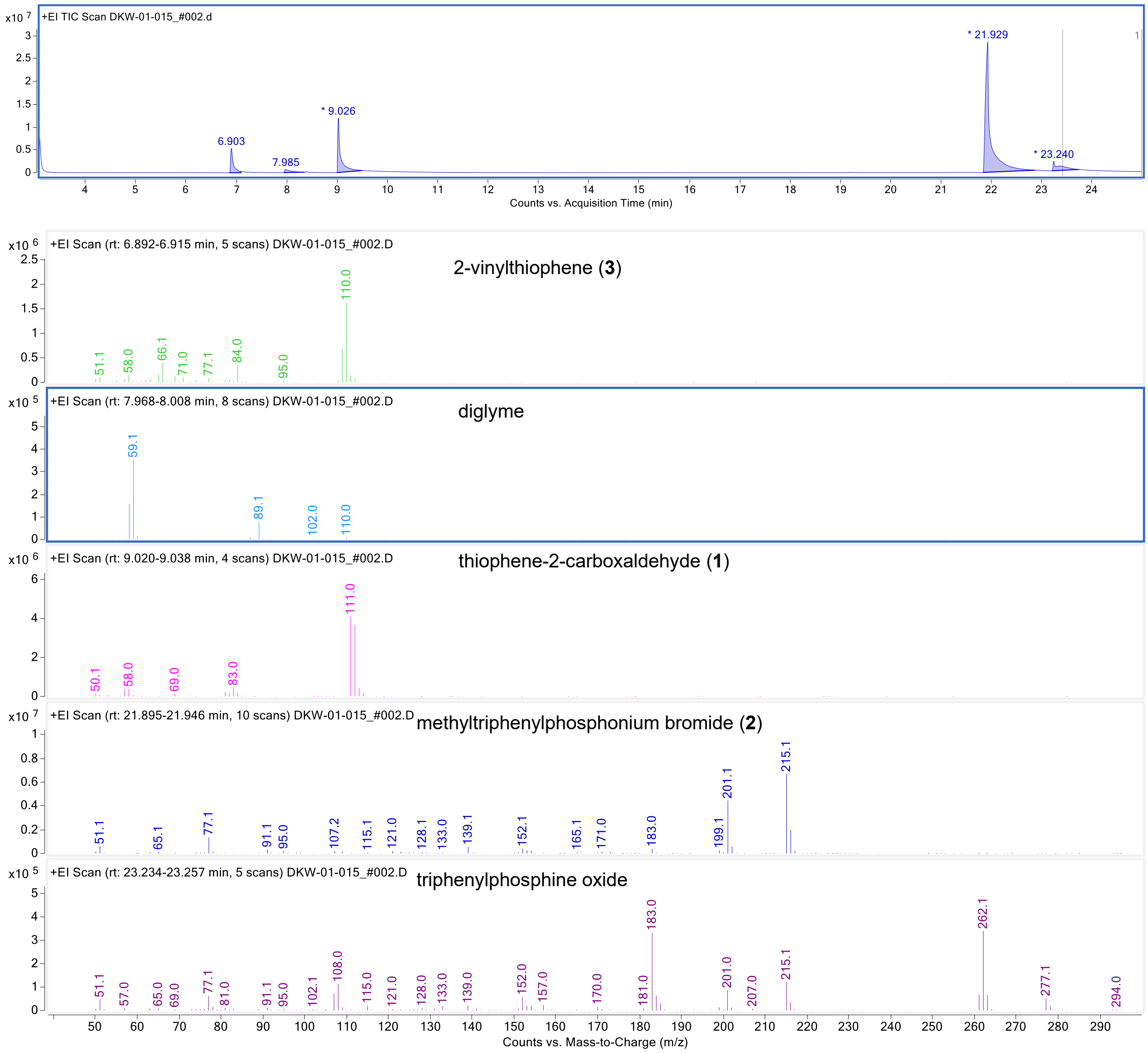

Figure S 1. Thiophene-2-carboxaldehyde – methyltriphenylphosphonium bromide Wittig reaction example GCMS chromatogram (top) and mass spectra (bottom five)

Grignard Reagent Formation and Peterson Olefination

Flow reactor setup

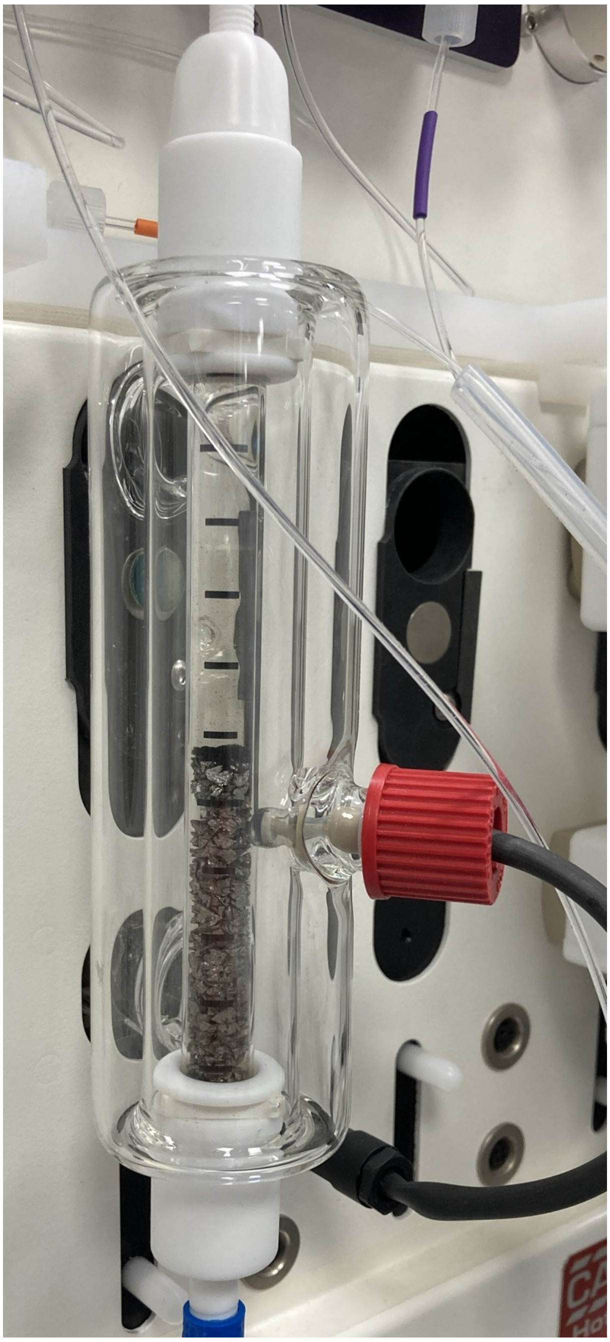

Figure S 2. Omnifit 150 × 6.6 mm glass column, partially filled with Mg turnings, in a Vapourtec air-heated glass manifold with temperature sensor

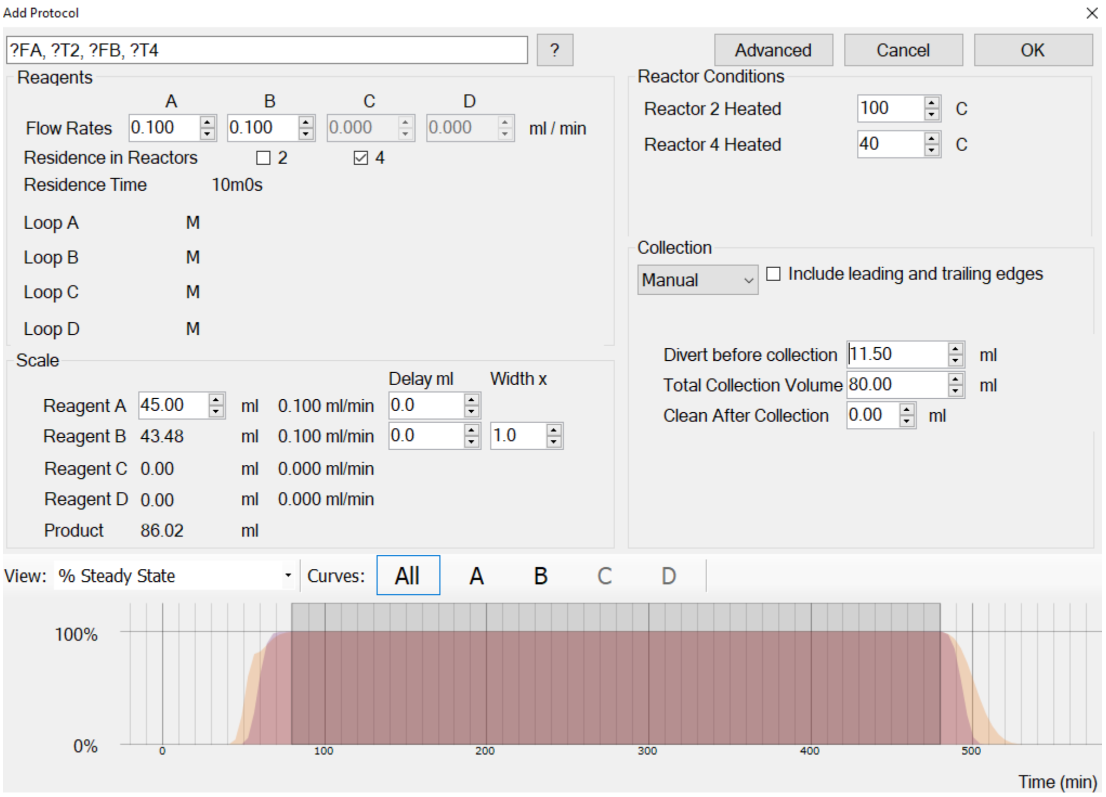

Figure S 3. Vapouretec Flow Commander screen used to take into account dispersion of reactant solutions through the tubing, predict when full contribution of reactant was achieved, and used to set collection time and amount.

GCMS Data

Experimental procedures and compound characterisation for the synthesis of 2-vinylthiophene are given in the main text. GCMS Method 2 data of starting material, product and side products, used during investigations, are given below.

Thiophene-2-carboxaldehyde (**1**) GCMS t_R = 7.9 min, *m/z* 112 (M^+, 56%), 110 ($[M-H]^+$, 100%), 58 (16%), 57 (5%). This data agrees with that given in the literature.[3]

2-Vinylthiophene (**3**) GCMS t_R = 5.8 min, *m/z* 110 (M^+, 100%), 109 ($[M-H]^+$, 45%), 84 (23%), 75 (1%). This data agrees with that given in the literature.[1]

Tentative structure: (E)-2,2'-(But-1-ene-1,3-diyl)dithiophene (**7**) (or isomer) GCMS t_R = 16.2 min, *m/z* 220 (M^+, 100%), 205 ($[M-Me]^+$, 82%), 172 (31%), 171 (52%), 147 (11%), 135 (13%), 121 (22%), 111 (13%), 109 (10%), 97 (32%). Compound present in literature but only electrospray ionisation mass spectrum reported.[6]

2-Methoxymethylthiophene (**9**) GCMS t_R = 8.9 min, *m/z* 128 (M^+, 38%), 113 (72%), 111 (25%), 97 (15%), 95 (20%), 85 (100%). This data agrees with that given in the literature except the literature does not report the peak at *m/z* 113.[7]

2-Acetylthiophene (**10**) GCMS t_R = 9.5 min, *m/z* 126 (M^+, 45%), 111 (100%). This data broadly agrees with that given in the literature.[8]

1,3-Di(thiophen-2-yl)prop-2-en-1-one (**11**) GCMS t_R = 16.2 min, *m/z* 220 (M^+, 13%), 191 (10%), 187 (3%), 147 (4%), 137 (4%), 111 (100%), 97 (35%), 65 (9%). This data broadly agrees with that given in the literature.[9]

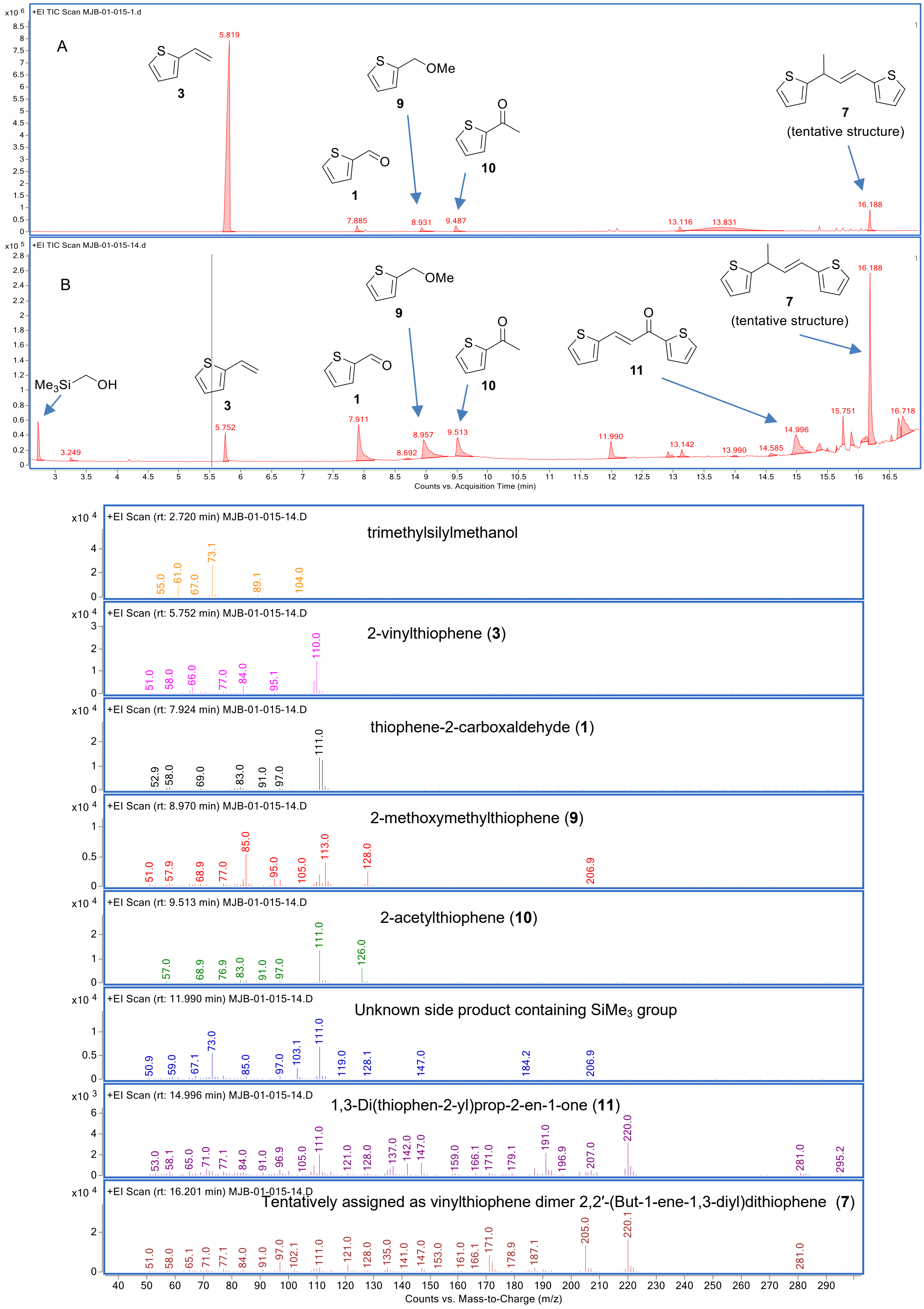

Figure S 4. GCMS Chromatograms and MS spectra resulting from trimethylsilylmethyl chloride conversion to Grignard reagent and reaction with thiophene-2-carboxaldehyde. A: typical reaction; B: reaction with < 1 equiv. Mg

NMR Spectra

2-Vinylthiophene (3)

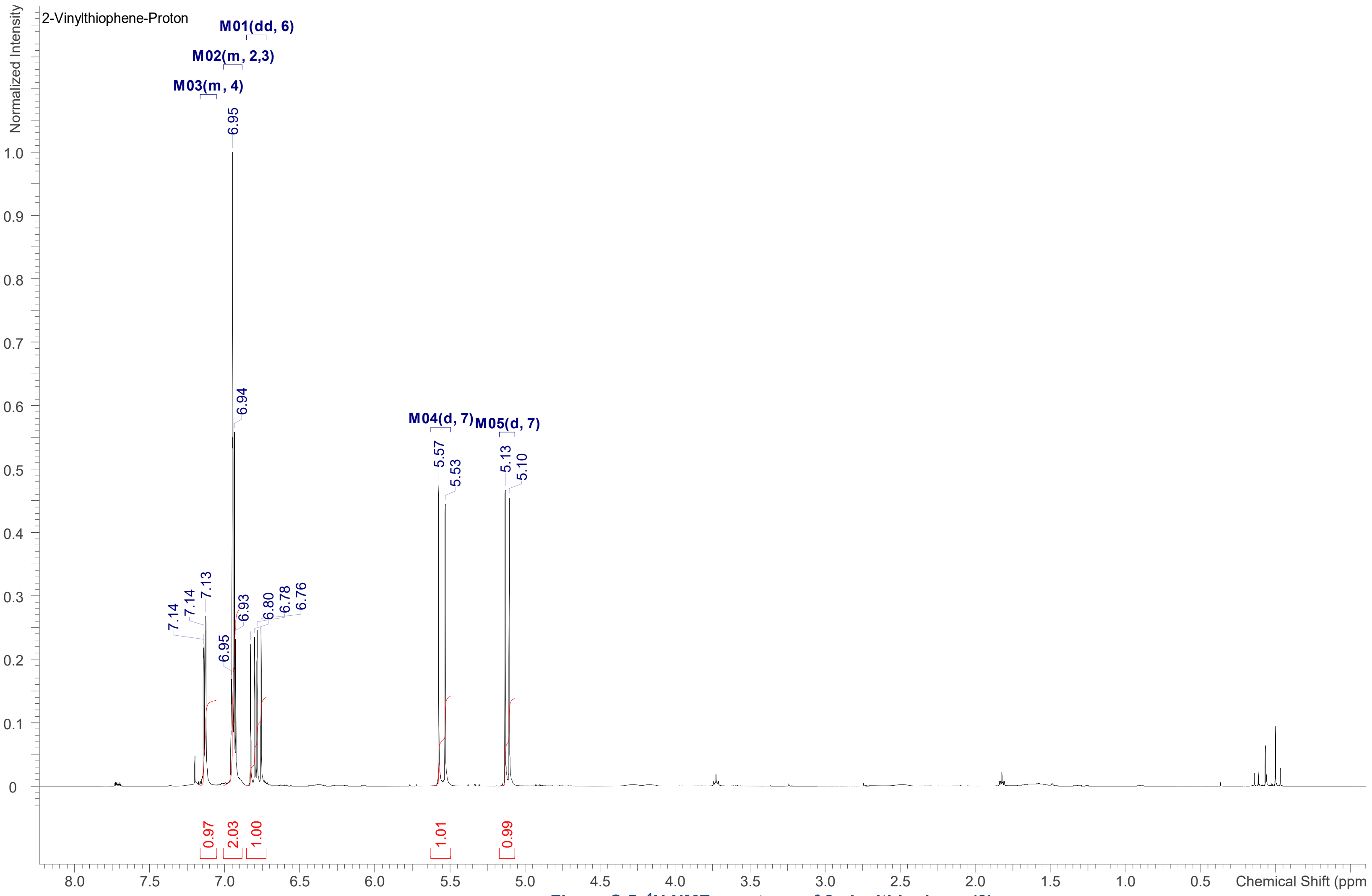

Figure S 5. ^{1}H NMR spectrum of 2-vinylthiophene (3)

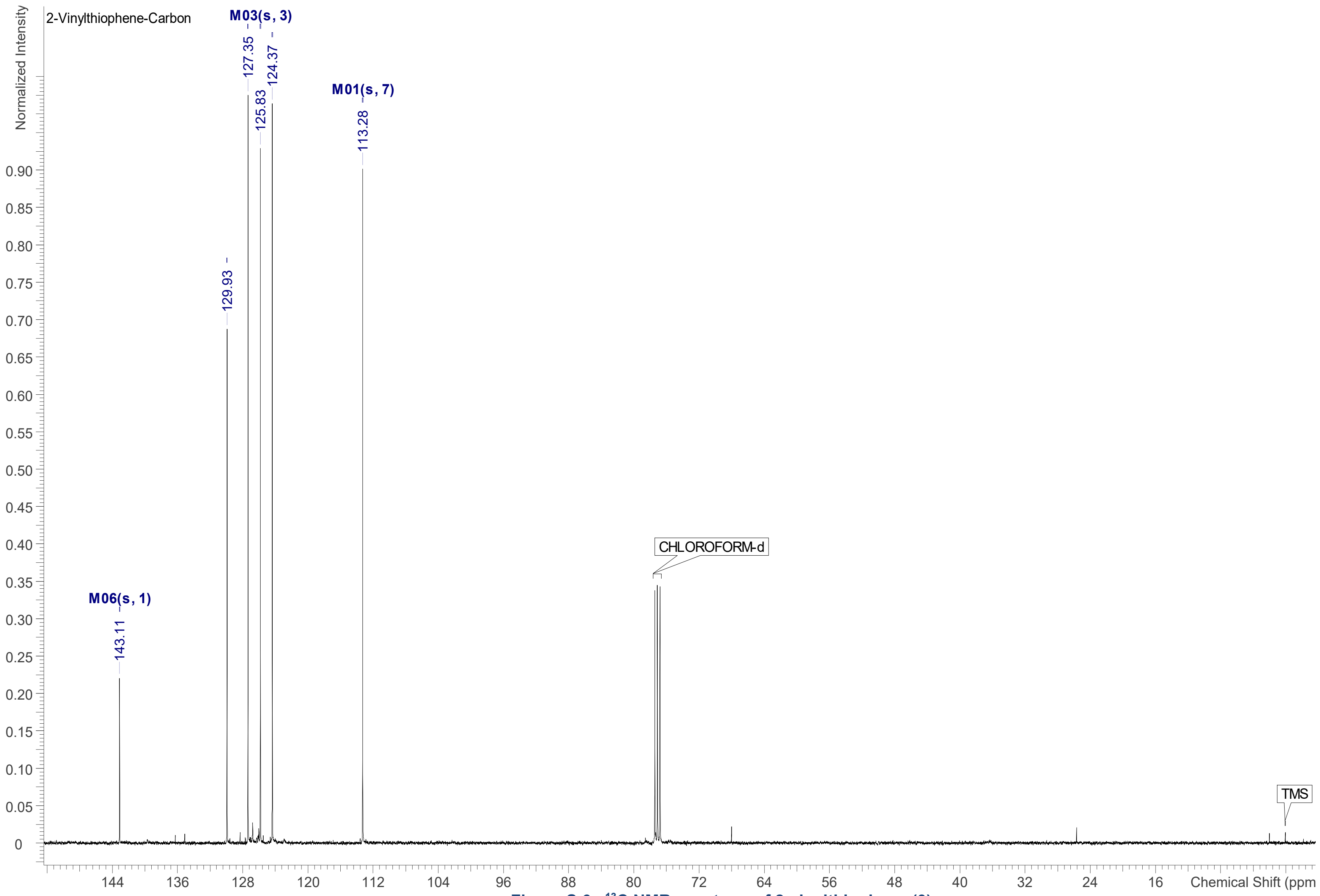

Figure S 6. ^{13}**C NMR spectrum of 2-vinylthiophene (3)**

2-Vinylfuran (12) (product : THF : Me_3SiOH = 48 : 6 : 46 mol%)

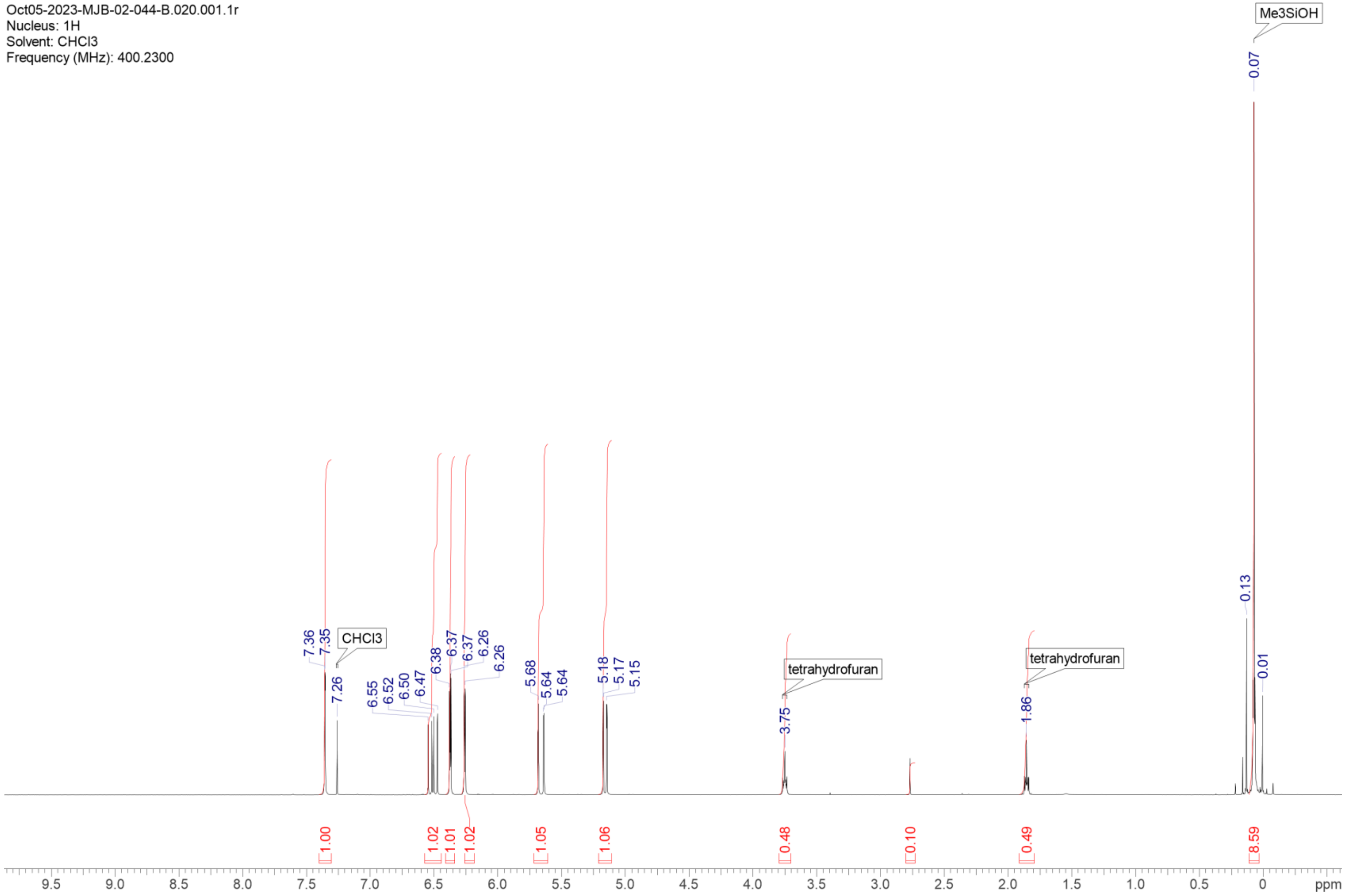

Figure S 7. ^{1}H NMR spectrum of 2-vinylfuran (12) (product : THF : Me_3SiOH = 48 : 6 : 46 mol%)

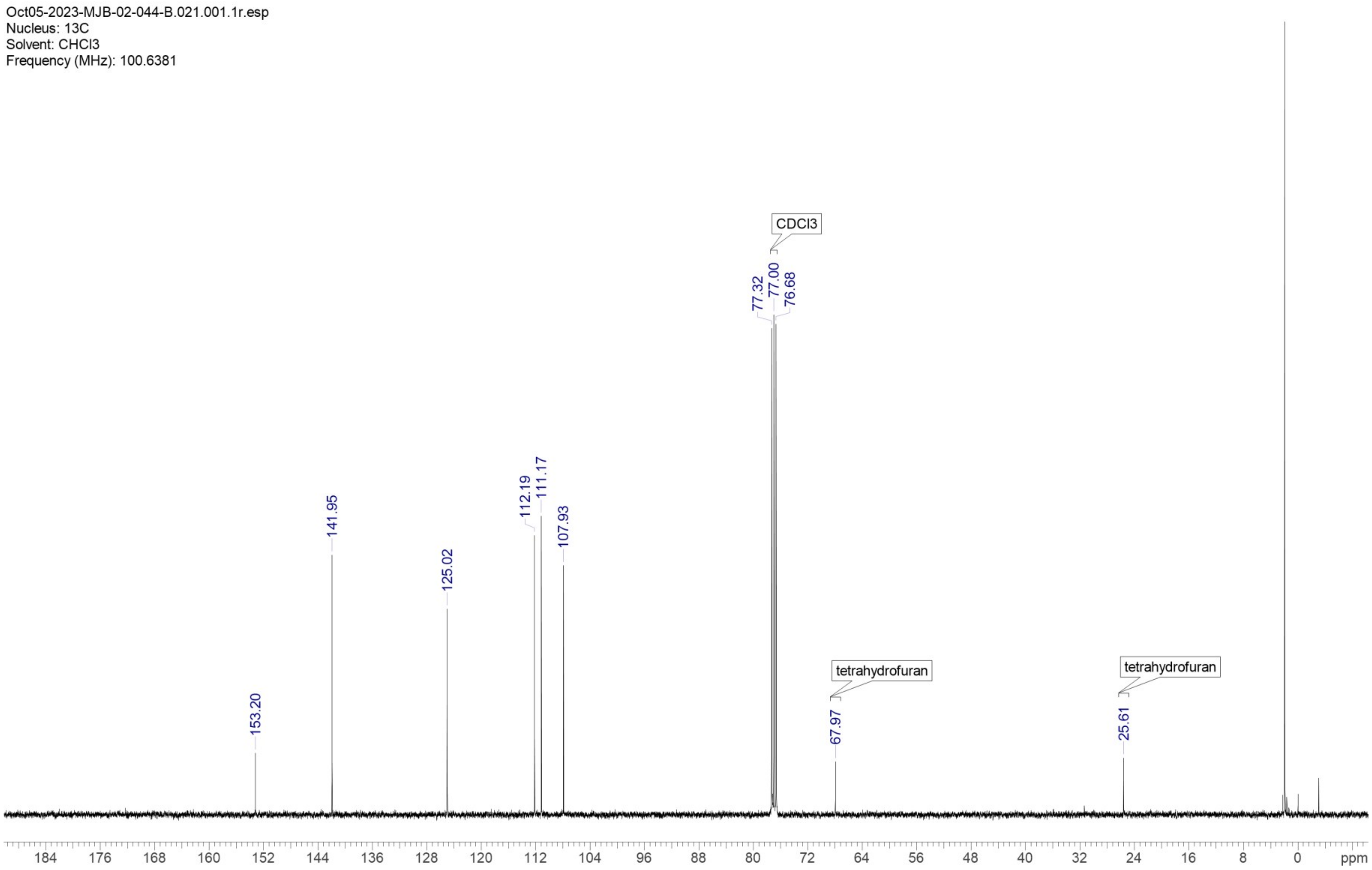

Figure S 8. ^{13}C NMR spectrum of 2-vinylfuran (12) (product : THF : Me_3SiOH = 48 : 6 : 46 mol%)

3-Methylstyrene (13)

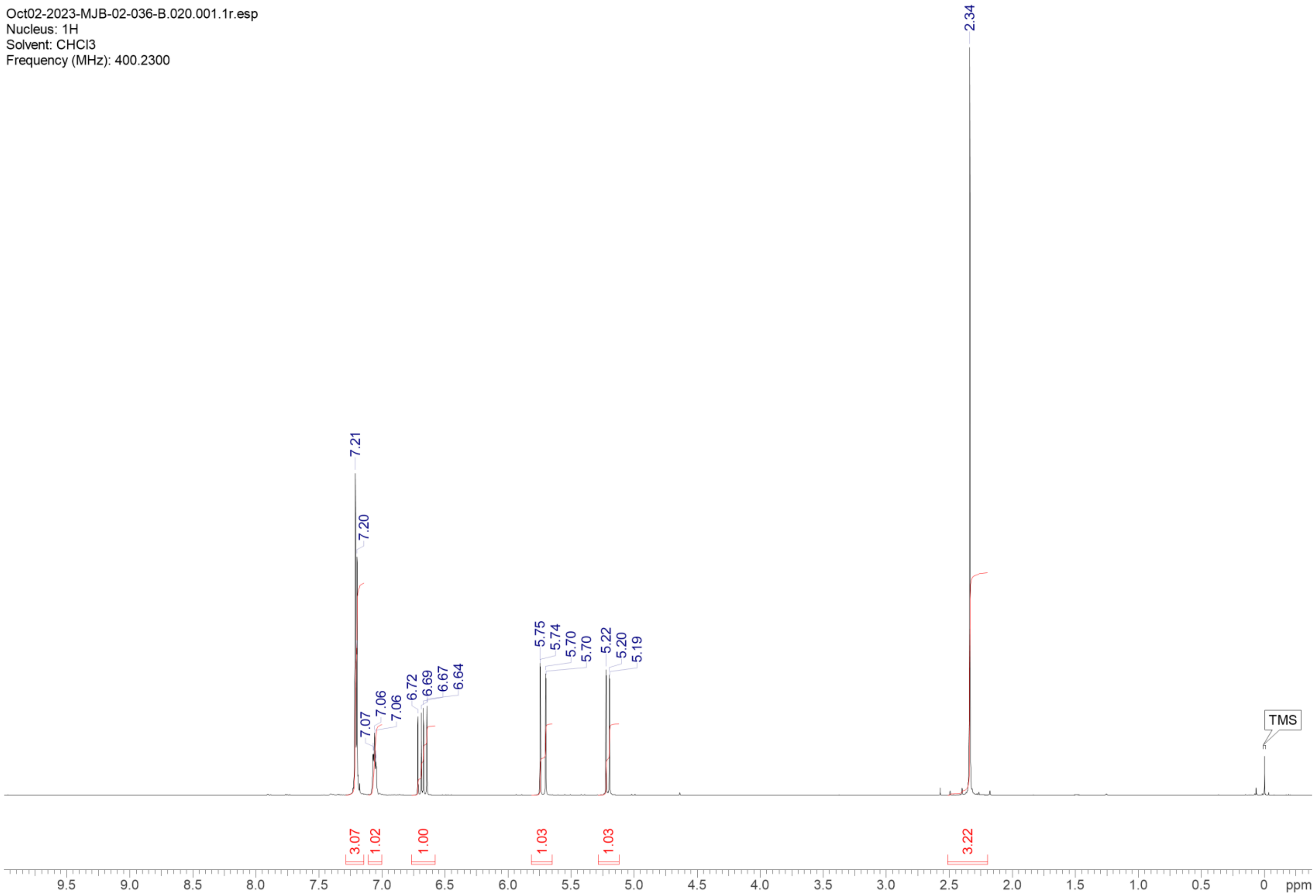

Figure S 9. ^{1}H NMR spectrum of 3-methylstyrene (13)

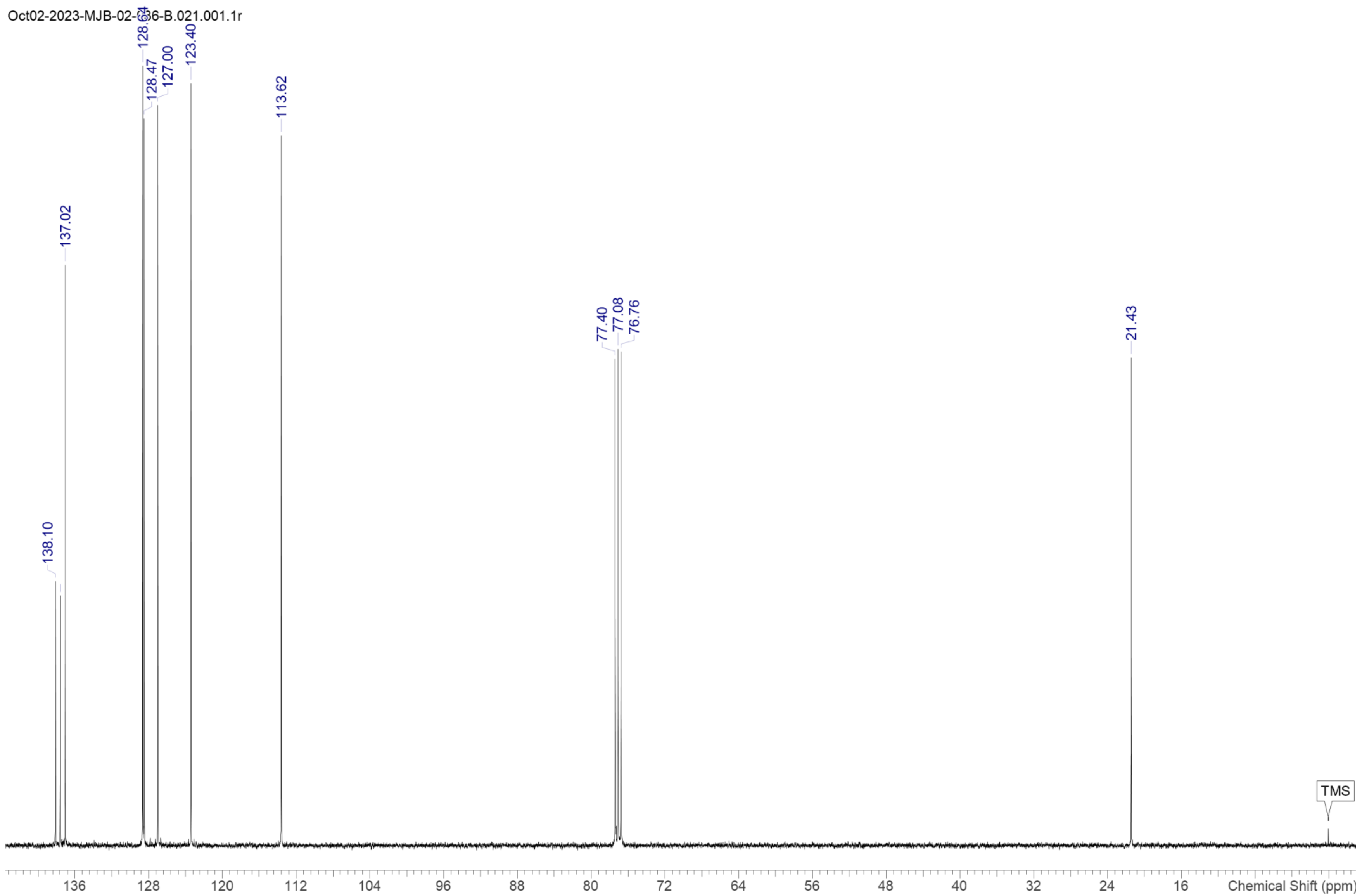

Figure S 10. ^{13}C NMR spectrum of 3-methylstyrene (13)

Matthew J. Burrell, John R. Varcoe, Daniel K. Whelligan

Methyl 4-vinylbenzoate (14)

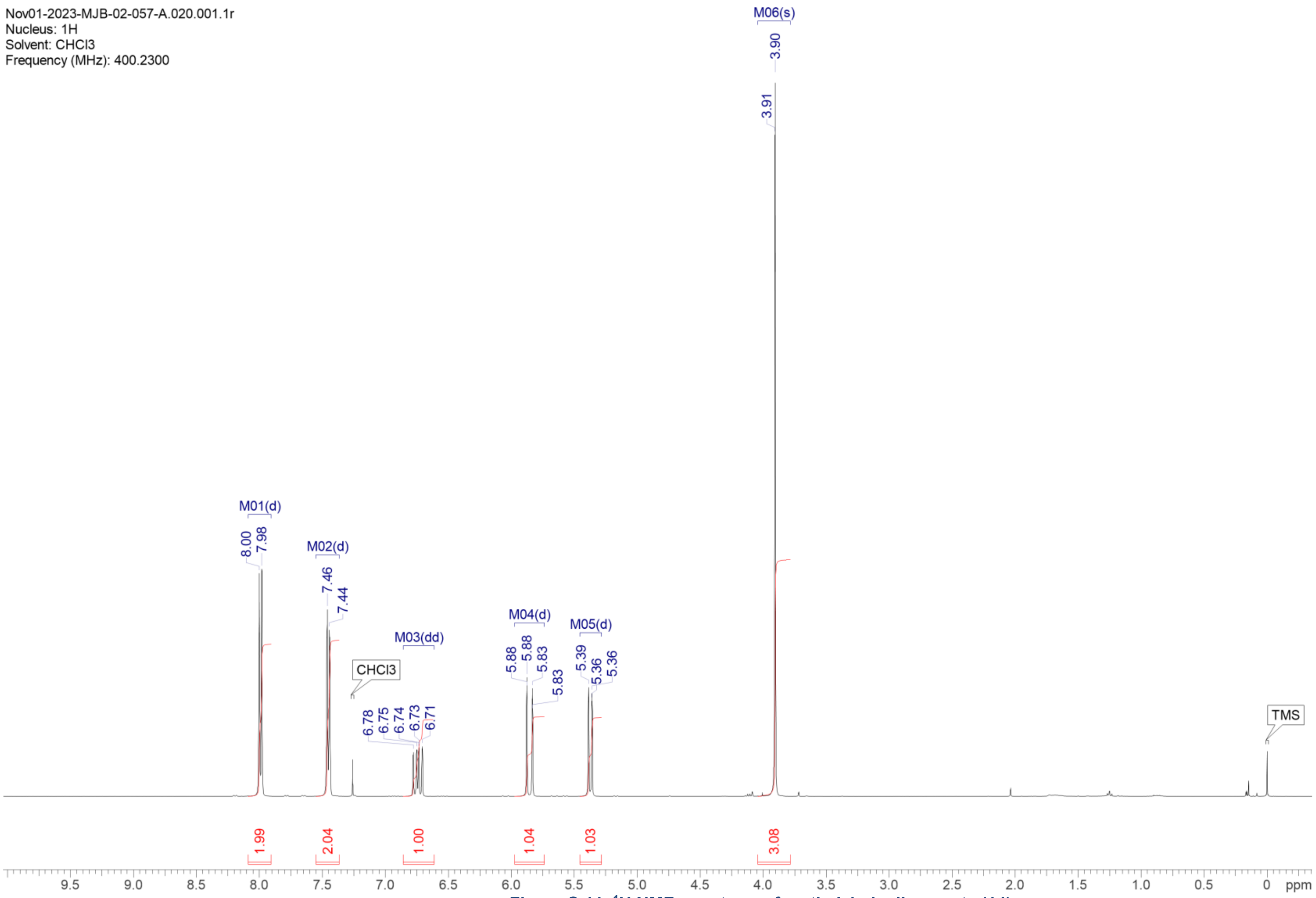

Figure S 11. ^{1}H NMR spectrum of methyl 4-vinylbenzoate (14)

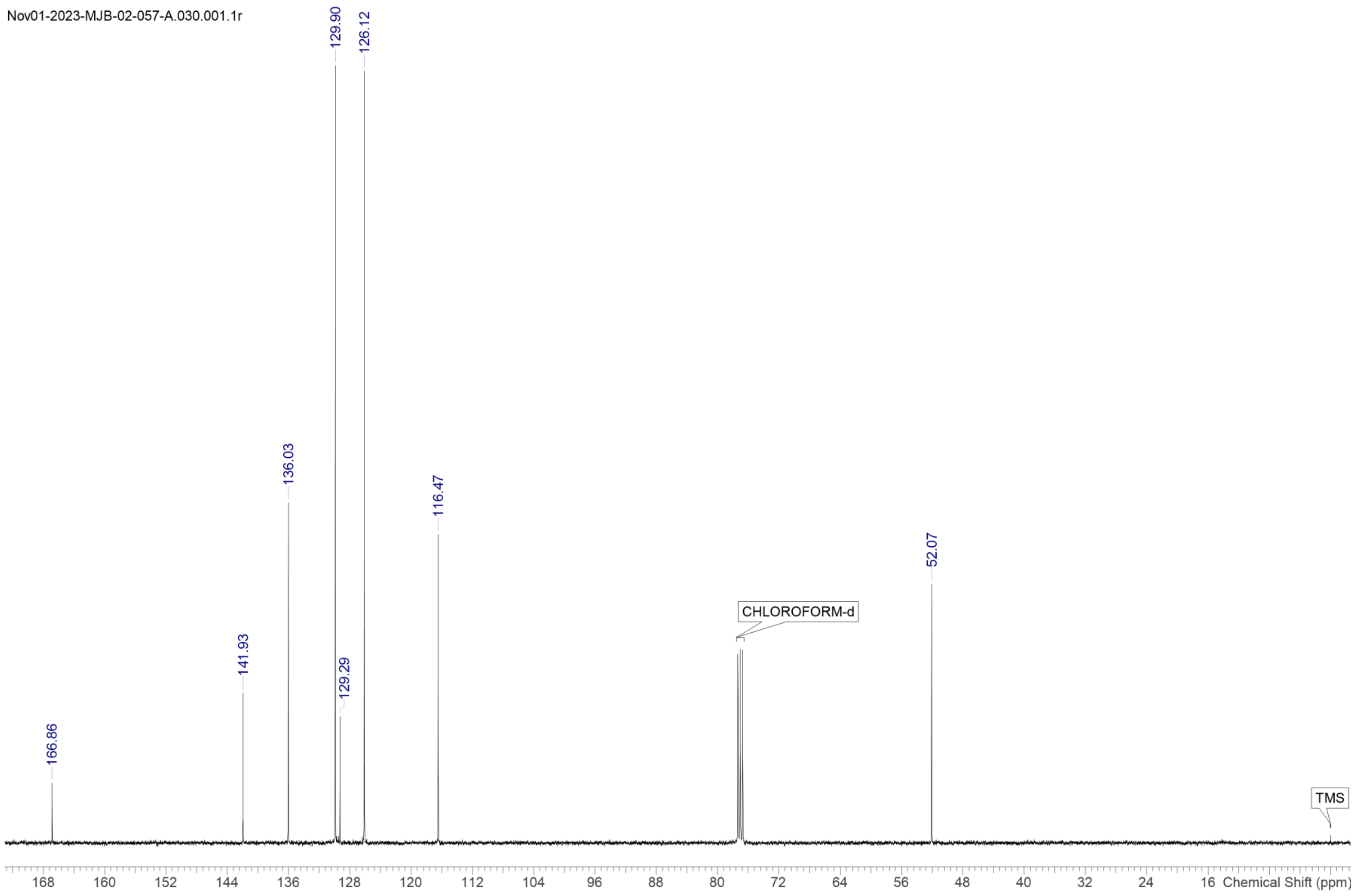

Figure S 12. ^{13}C NMR spectrum of methyl 4-vinylbenzoate (14)

2-(Dimethylphenylsilyl)-1-(1-methyl-1H-imidazol-2-yl)ethan-1-ol (21)

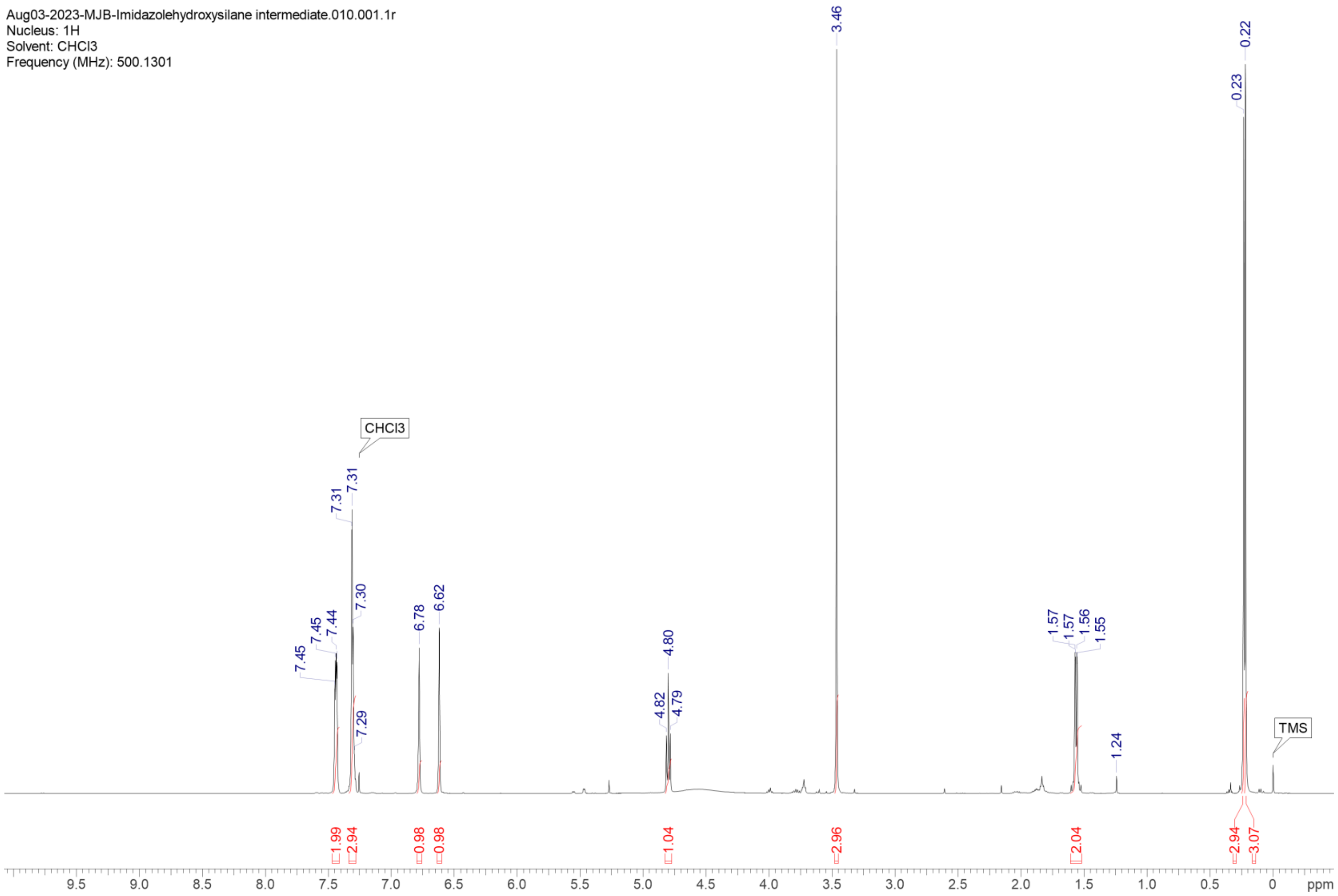

Figure S 13. ^{1}H NMR spectrum of 2-(dimethylphenylsilyl)-1-(1-methyl-1H-imidazol-2-yl)ethan-1-ol (21)

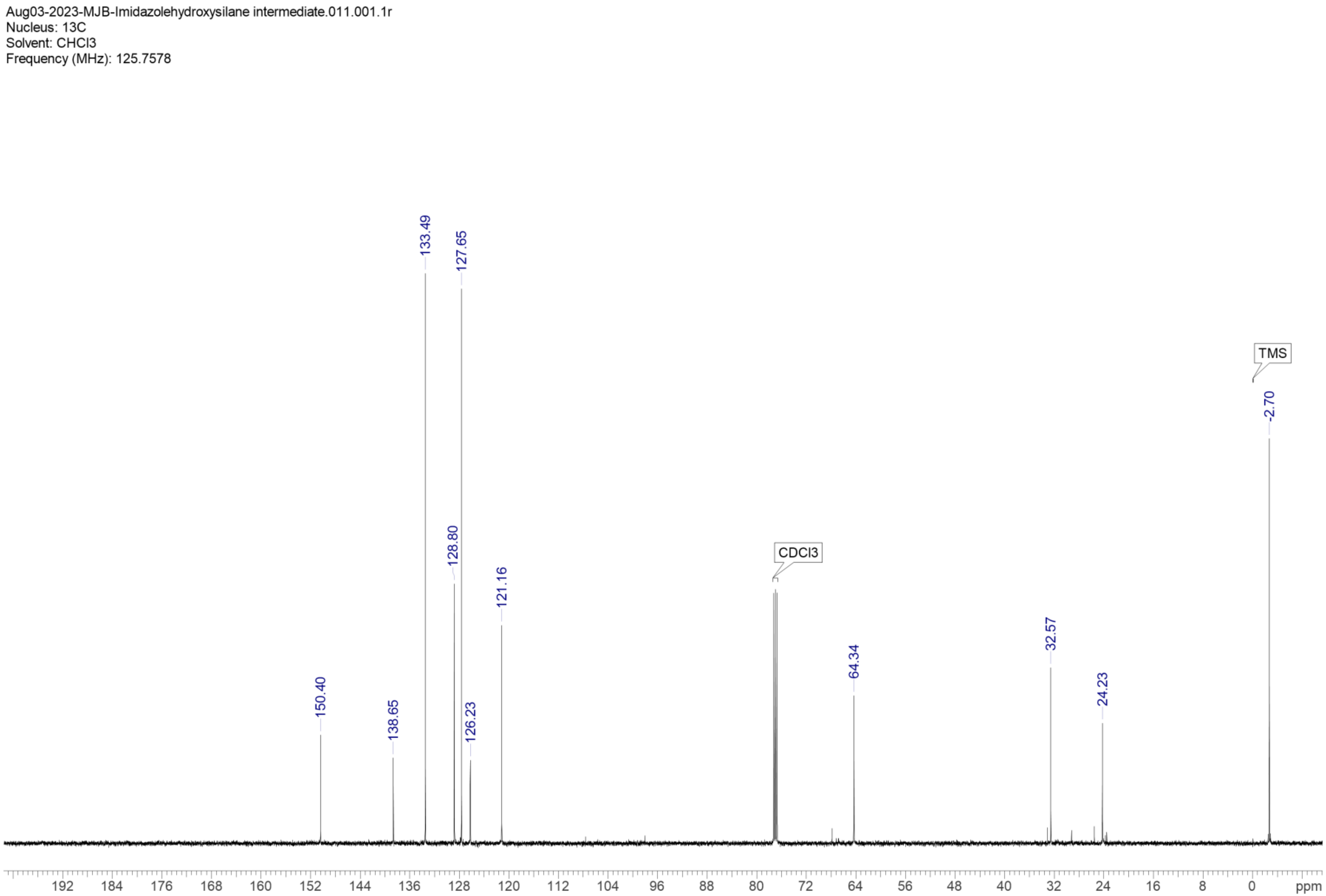

Figure S 14. ^{13}C NMR spectrum of 2-(dimethylphenylsilyl)-1-(1-methyl-1H-imidazol-2-yl)ethan-1-ol (21)

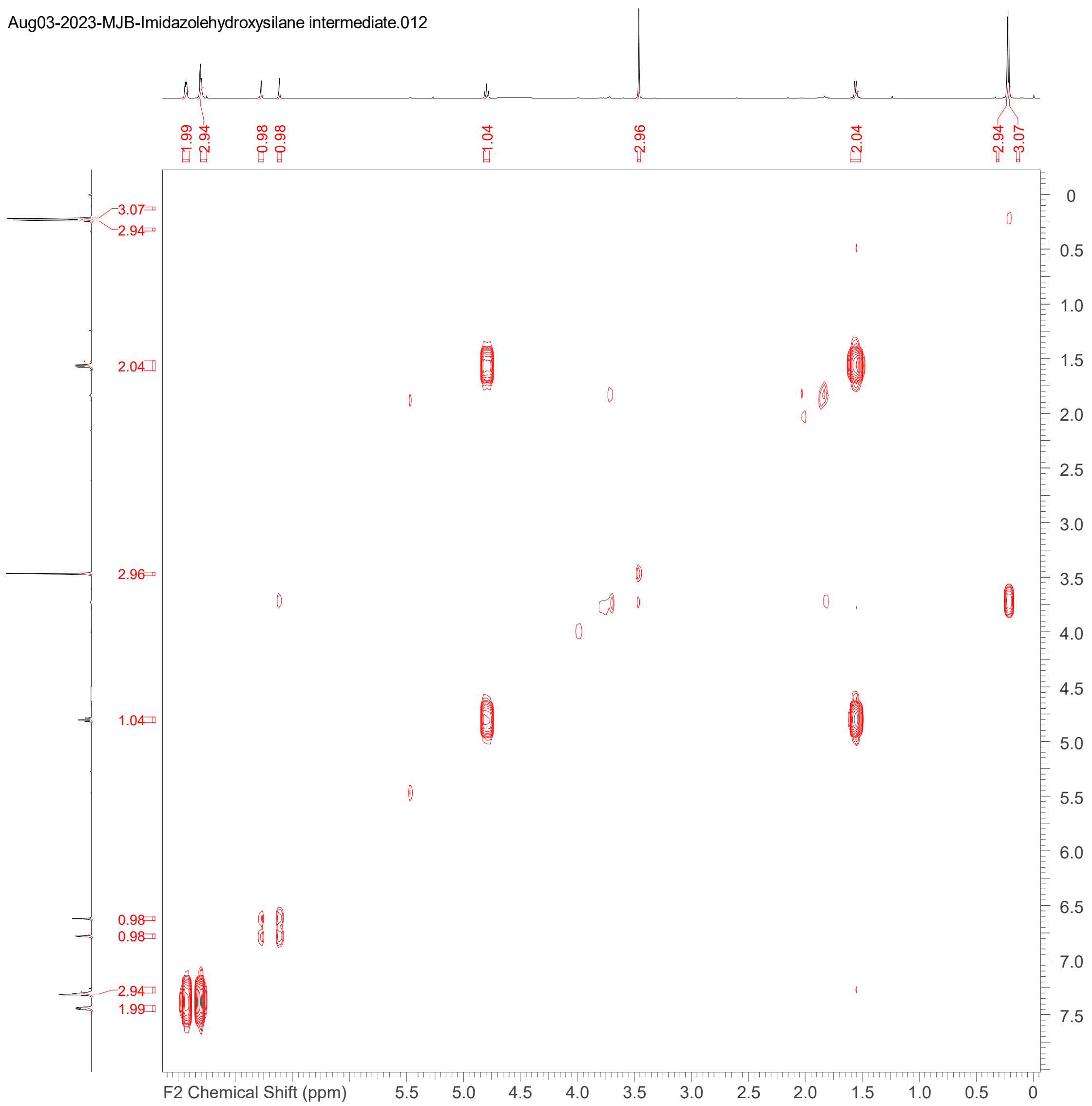

Figure S 15. 1H COSY spectrum of 2-(dimethylphenylsilyl)-1-(1-methyl-1H-imidazol-2-yl)ethan-1-ol (21)

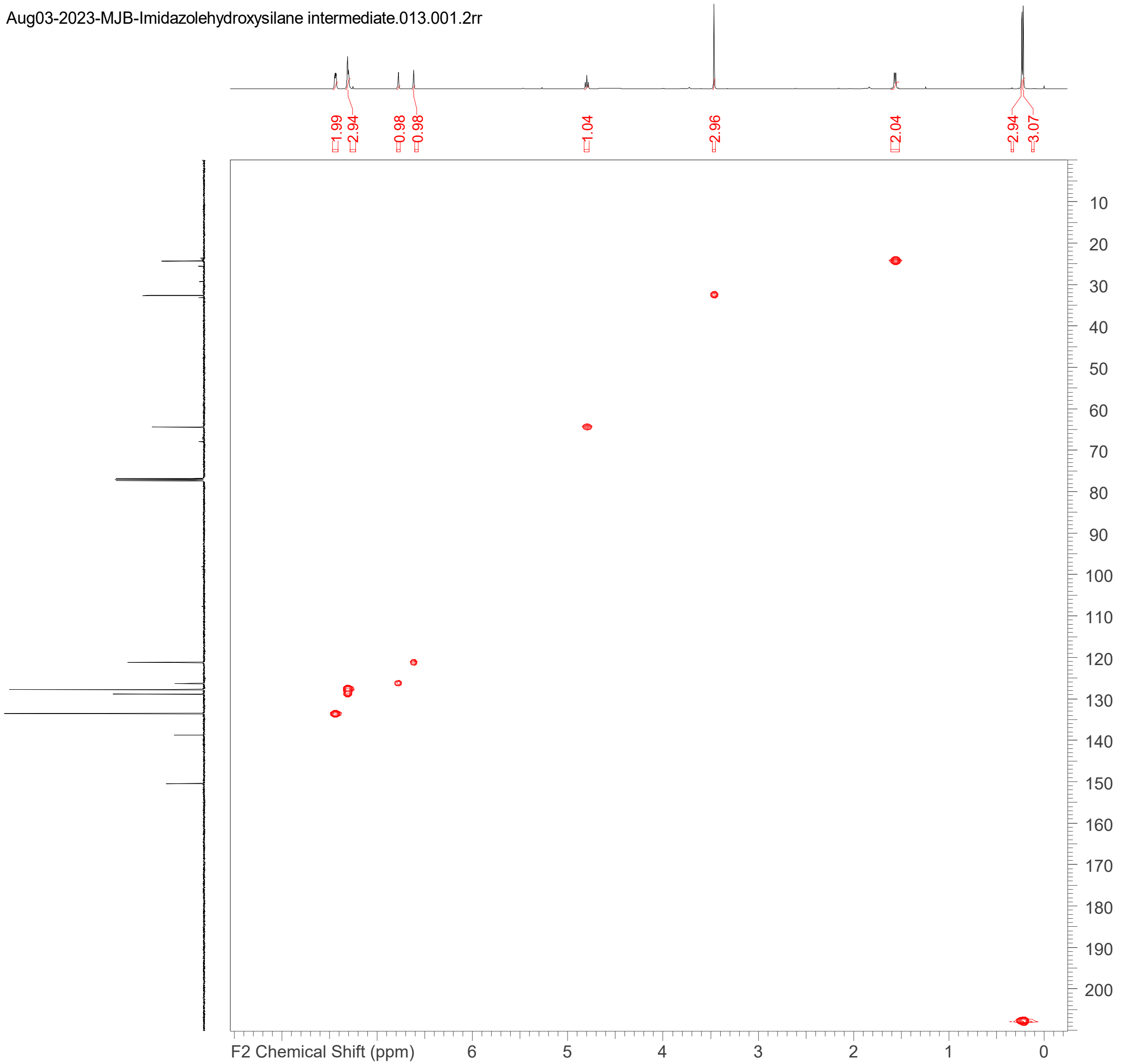

Figure S 16. HSQC spectrum of 2-(dimethylphenylsilyl)-1-(1-methyl-1H-imidazol-2-yl)ethan-1-ol (21) – note spectrum has 'wrapped around' peaks below zero to above 200 ppm

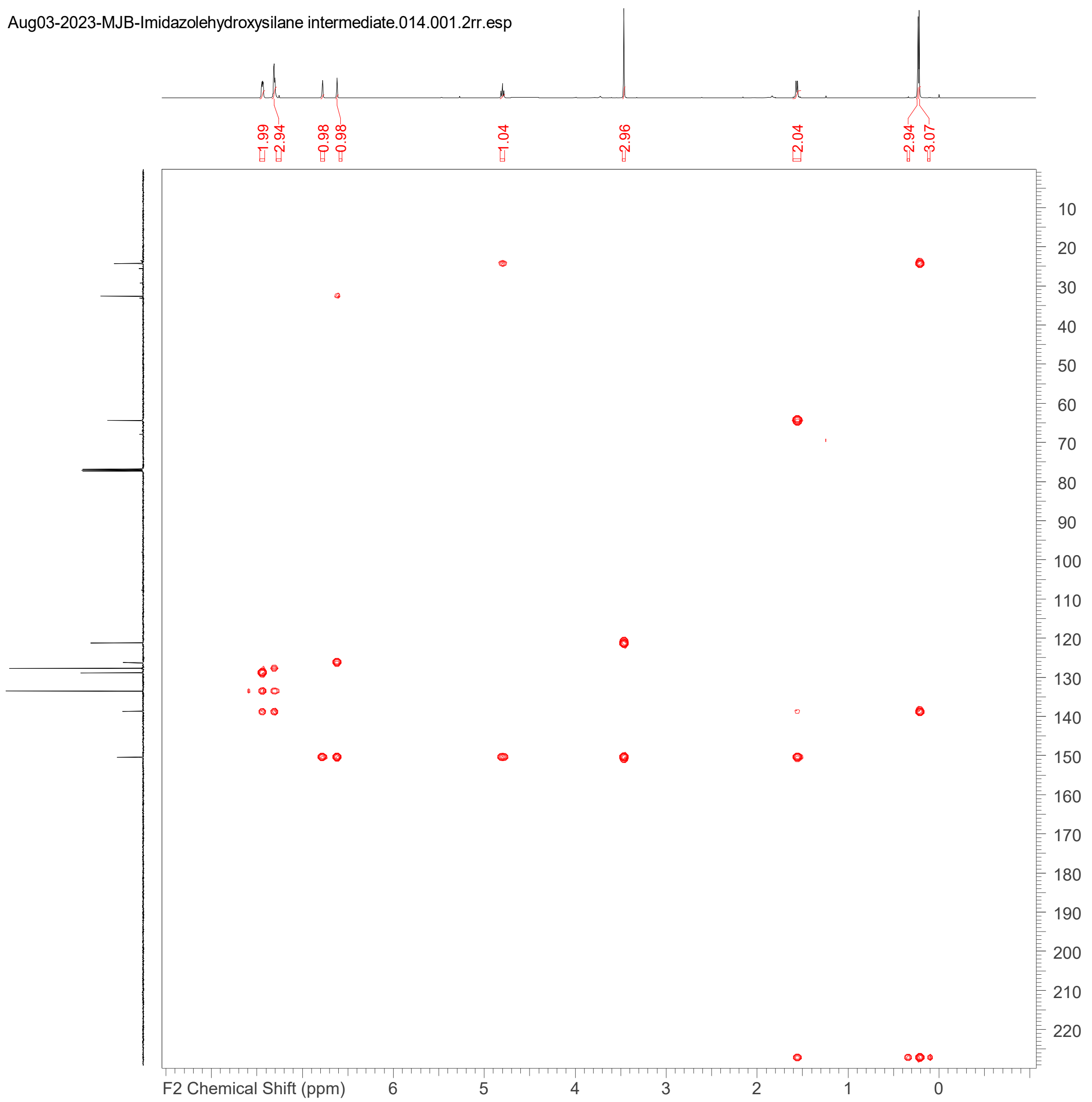

Figure S 17. HMBC spectrum of 2-(dimethylphenylsilyl)-1-(1-methyl-1H-imidazol-2-yl)ethan-1-ol (21) – note spectrum has 'wrapped around' peaks below zero to above 200 ppm

2-(Dimethylphenylsilyl)-1-(pyridine-3-yl)ethan-1-ol (22)

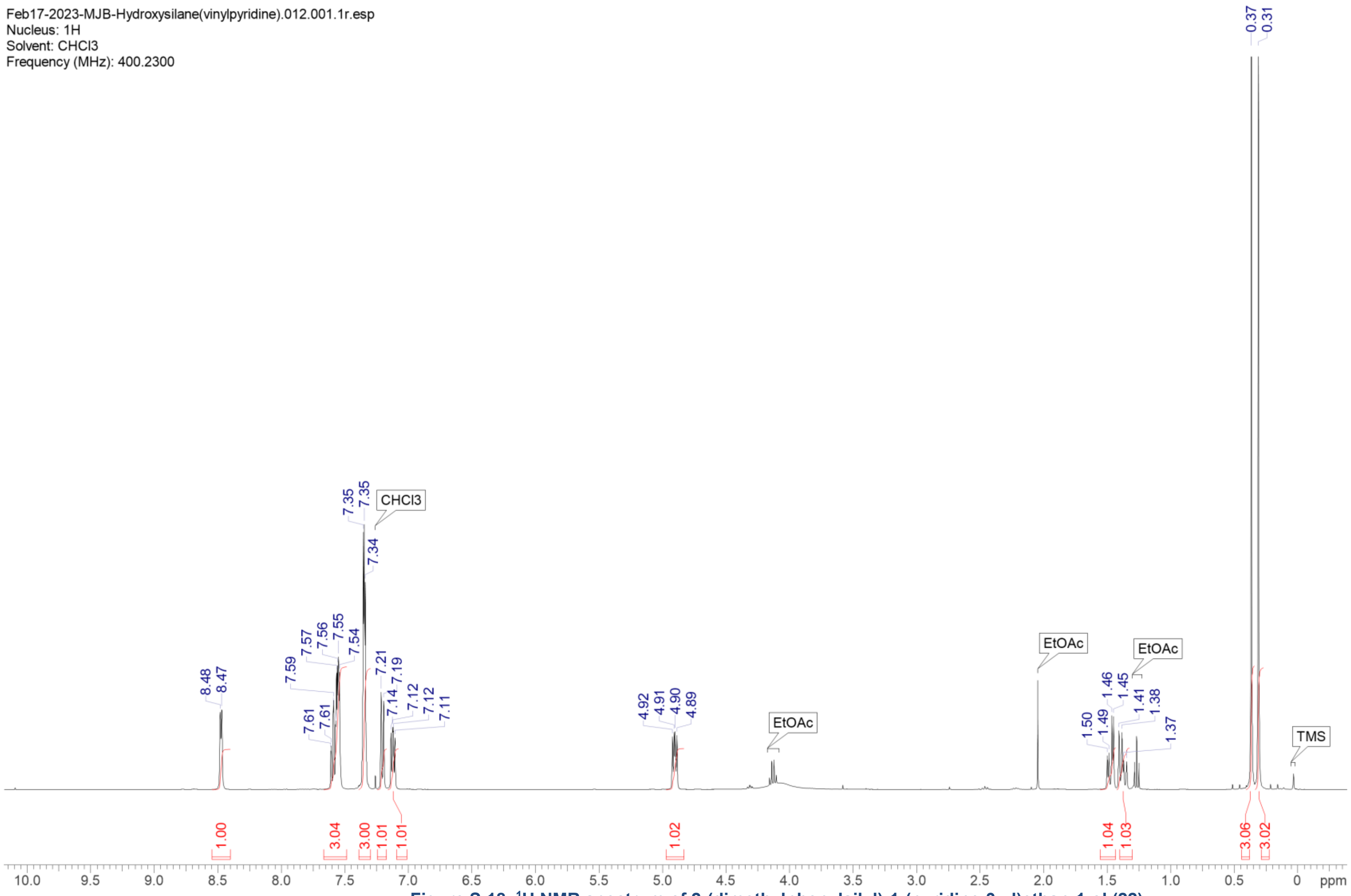

Figure S 18. **^{1}H NMR spectrum of 2-(dimethylphenylsilyl)-1-(pyridine-3-yl)ethan-1-ol (22)**

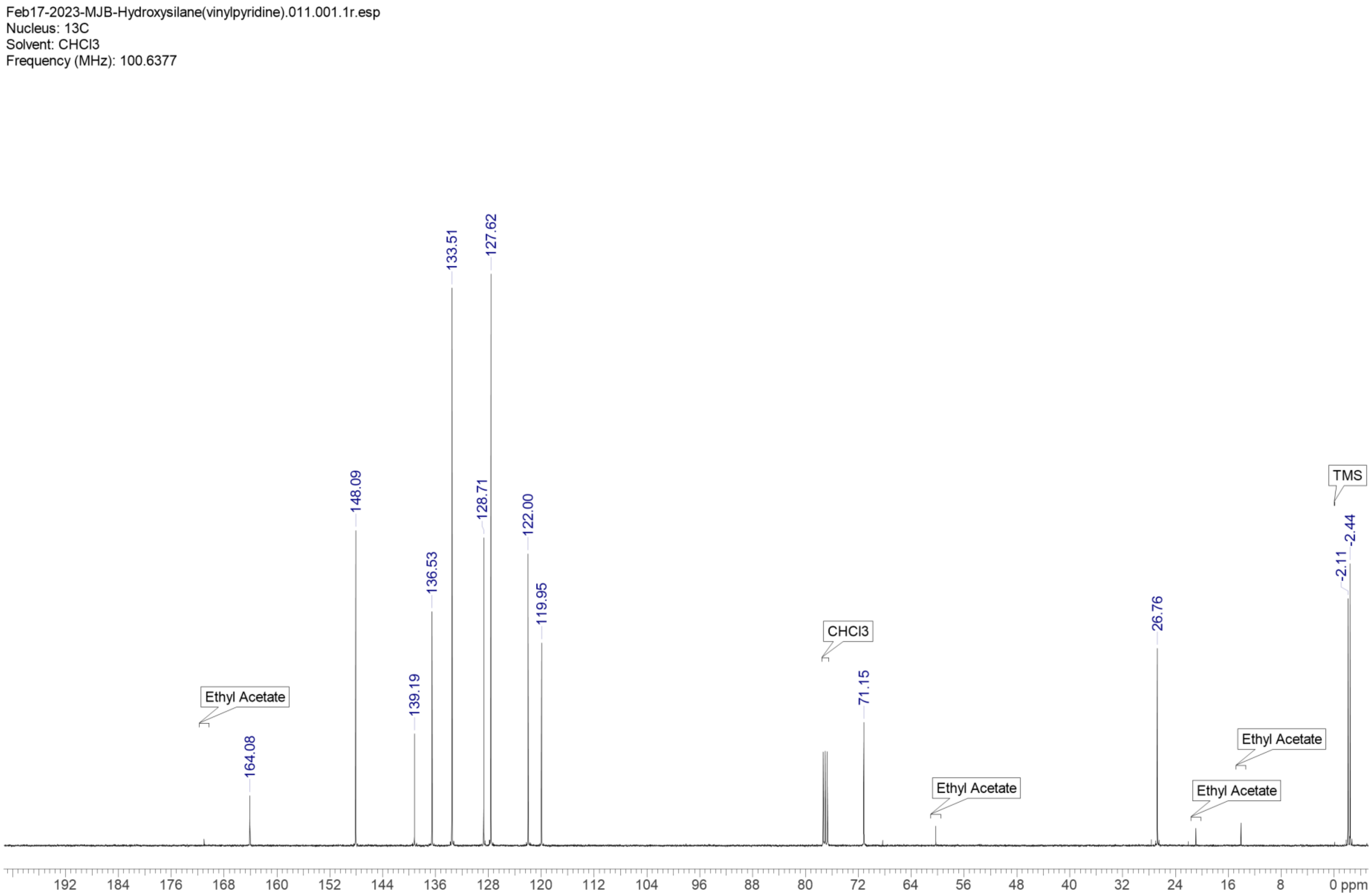

Figure S 19. ^{13}C NMR spectrum of 2-(dimethylphenylsilyl)-1-(pyridine-3-yl)ethan-1-ol (22)

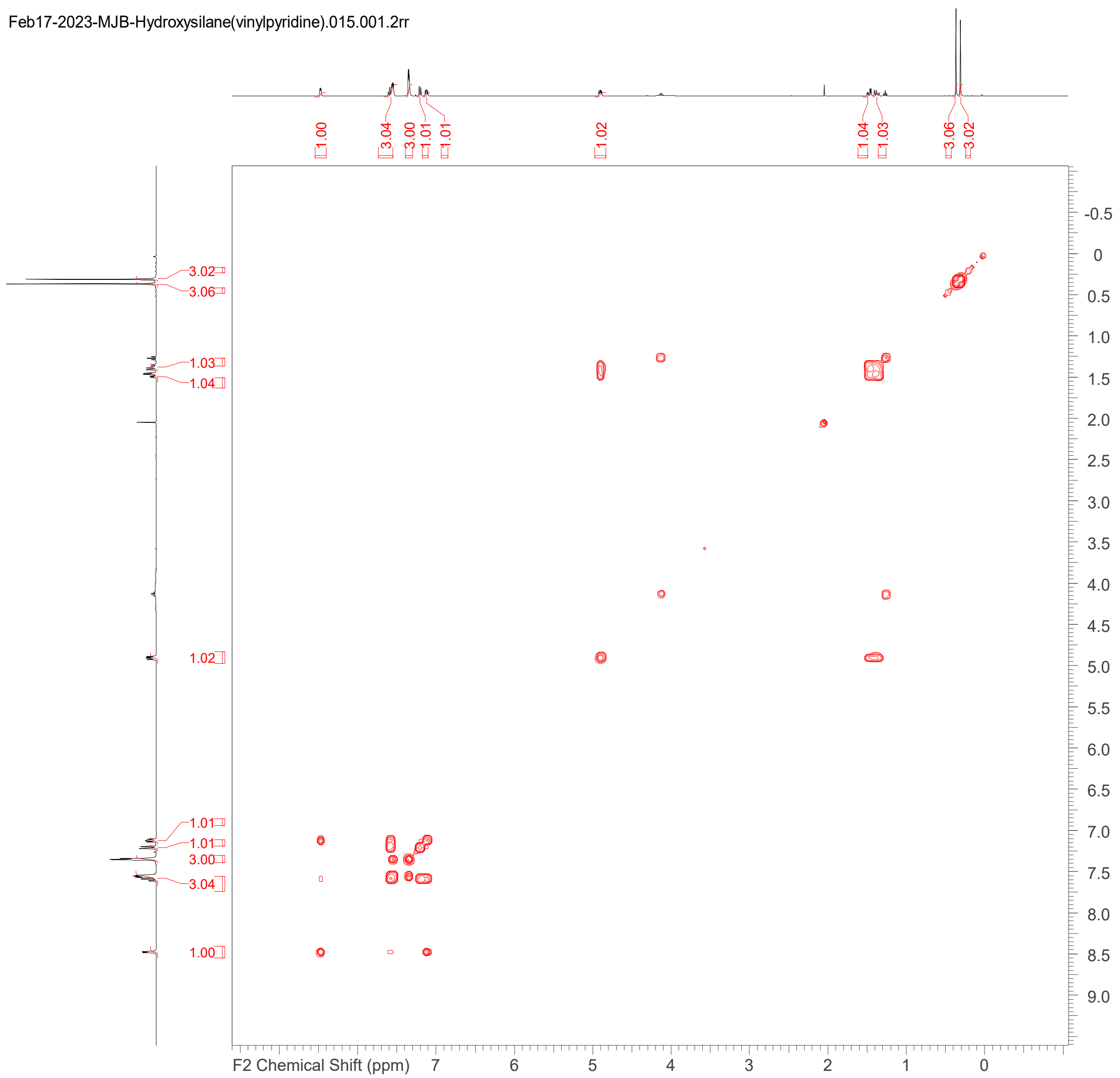

Figure S 20. ^{1}H COSY spectrum of 2-(dimethylphenylsilyl)-1-(pyridine-3-yl)ethan-1-ol (22)

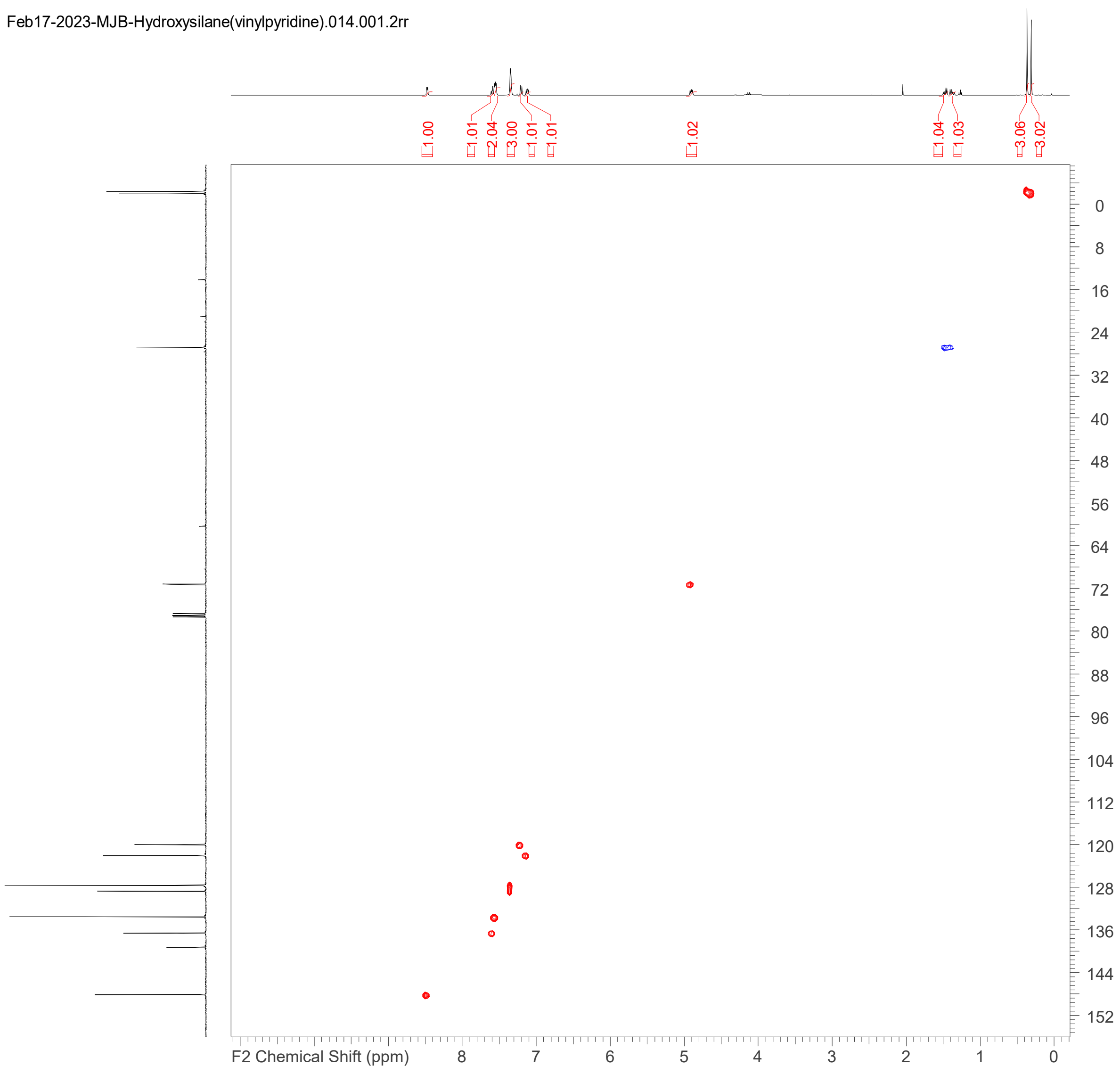

Figure S 21. HSQC spectrum of 2-(dimethylphenylsilyl)-1-(pyridine-3-yl)ethan-1-ol (22)

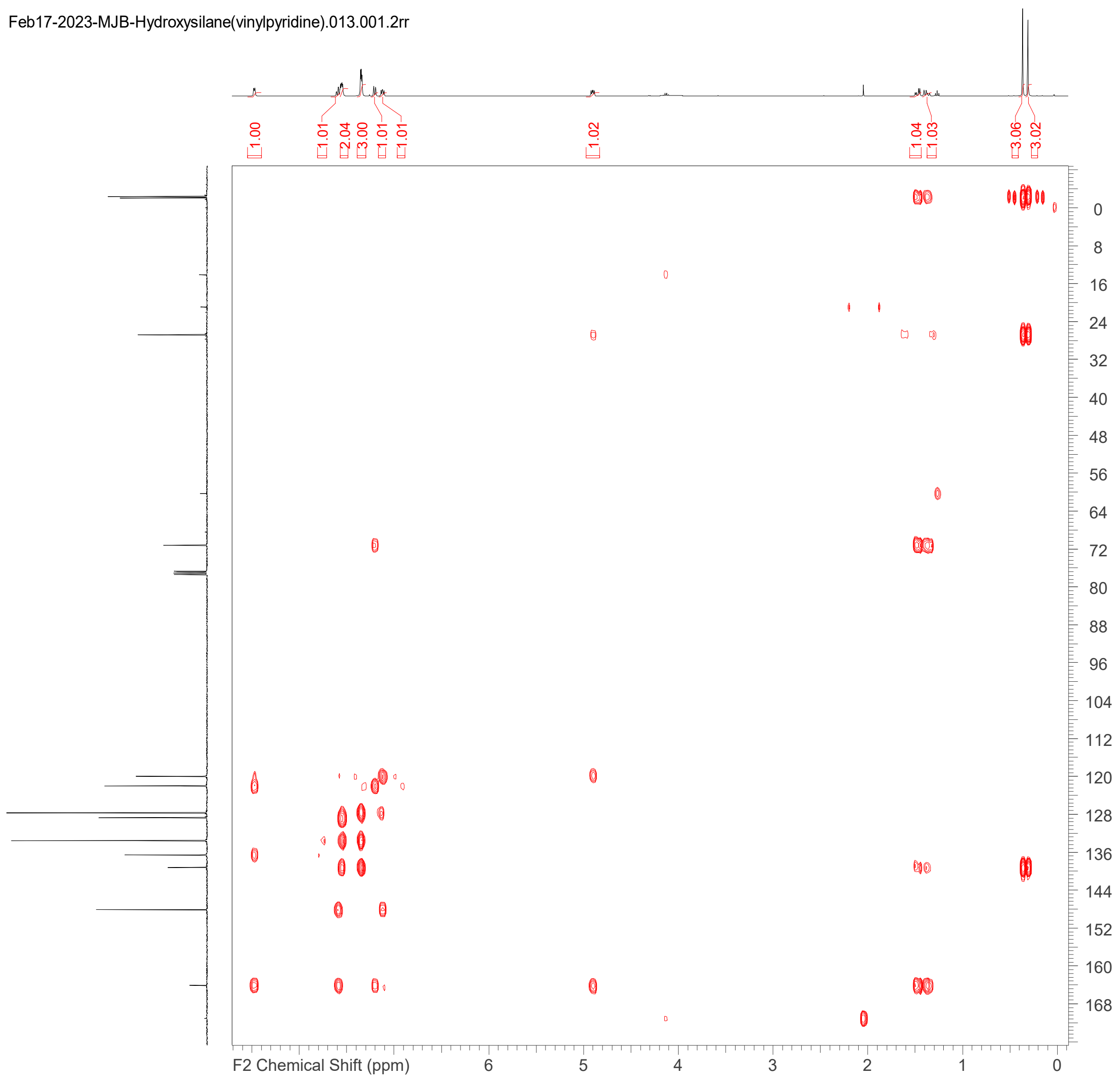

Figure S 22. HMBC spectrum of 2-(dimethylphenylsilyl)-1-(pyridine-3-yl)ethan-1-ol (22)

1-Methyl-2-vinylimidazole (15)

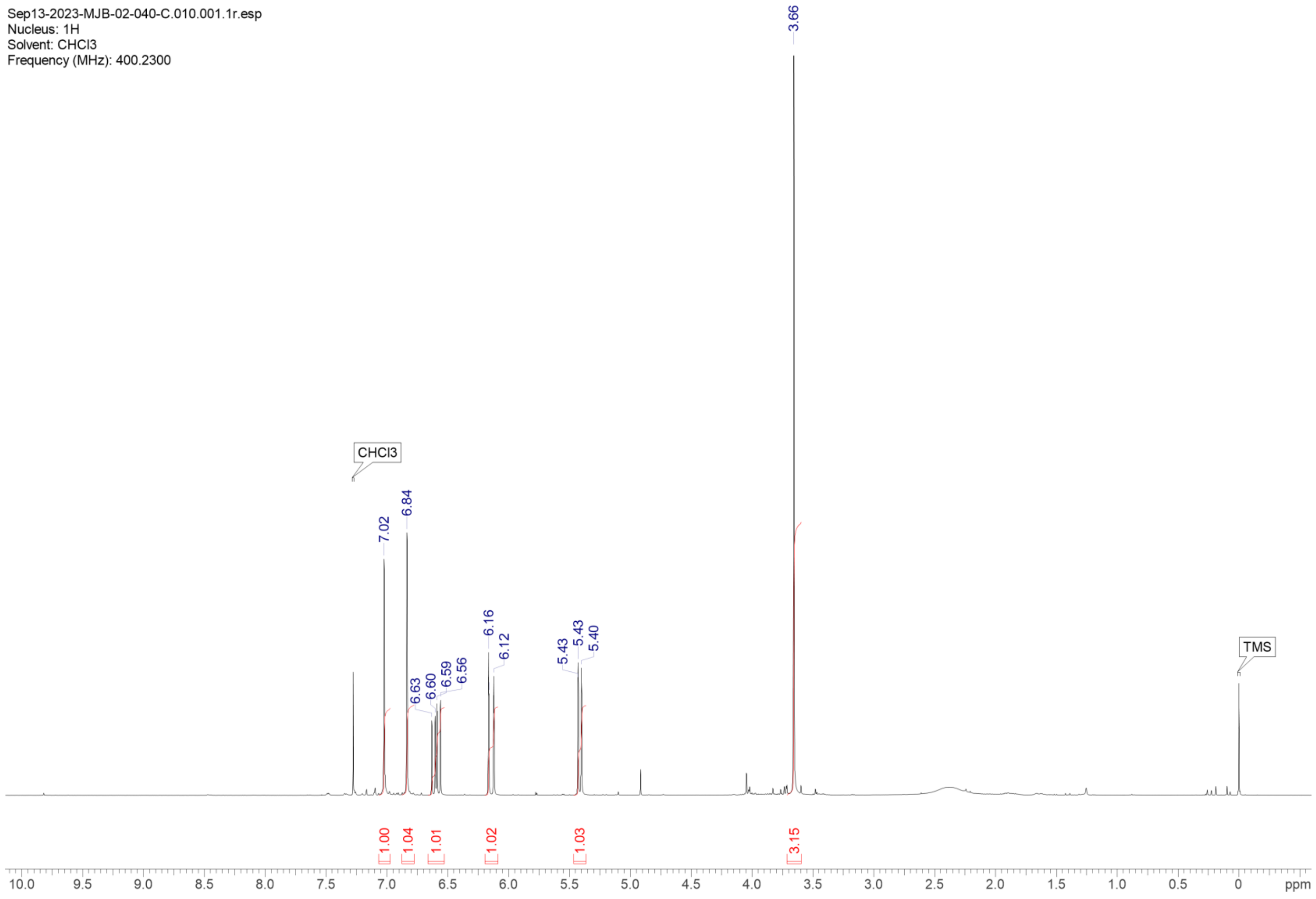

Figure S 23. ^{1}H NMR spectrum of 1-methyl-2-vinylimidazole (15)

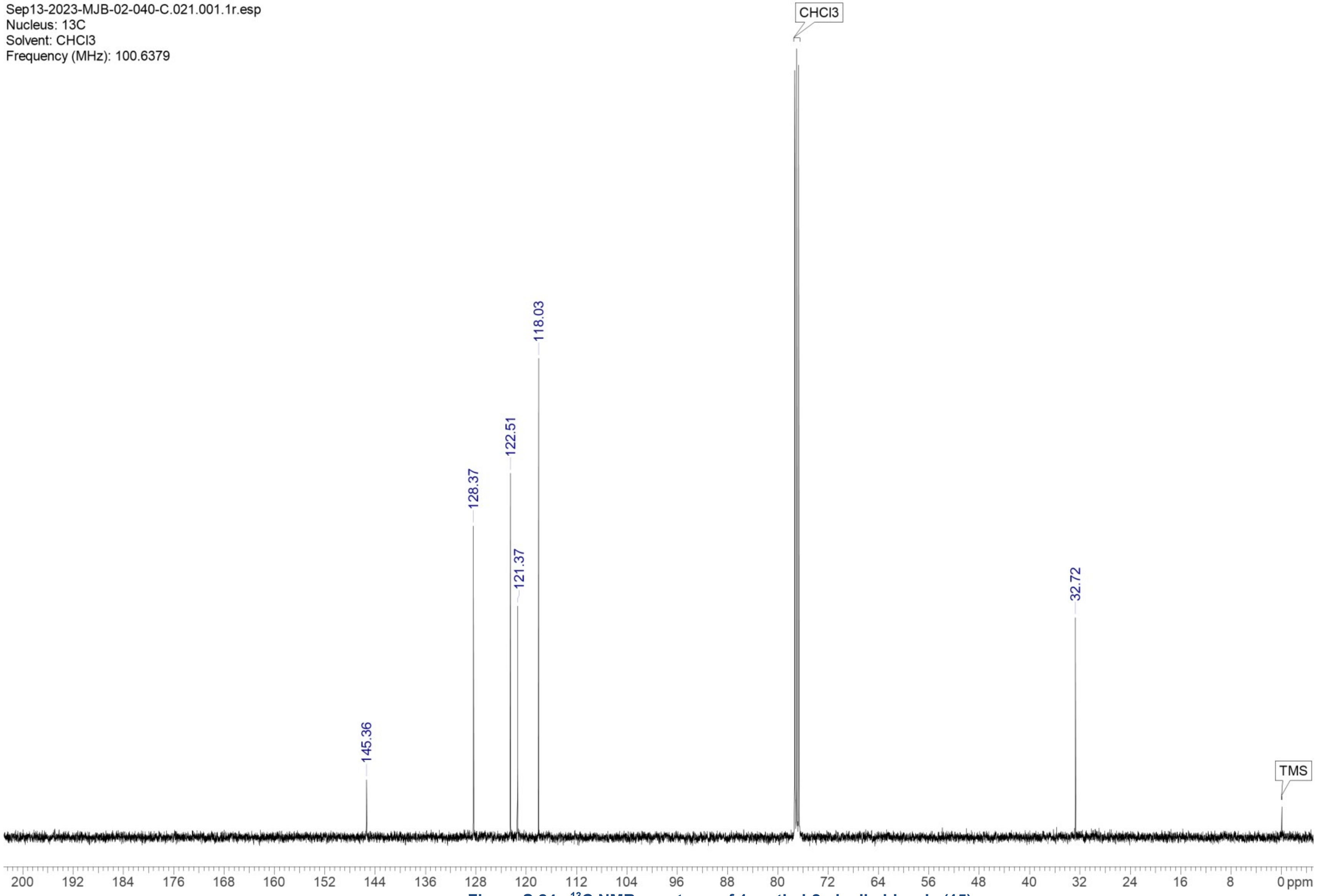

Figure S 24. ^{13}C **NMR spectrum of 1-methyl-2-vinylimidazole (15)**

3-Vinylpyridine (16, present in THF, benzyl benzoate internal standard): 50 mg solution in THF + 2.86 mg internal standard in $CDCl_3$

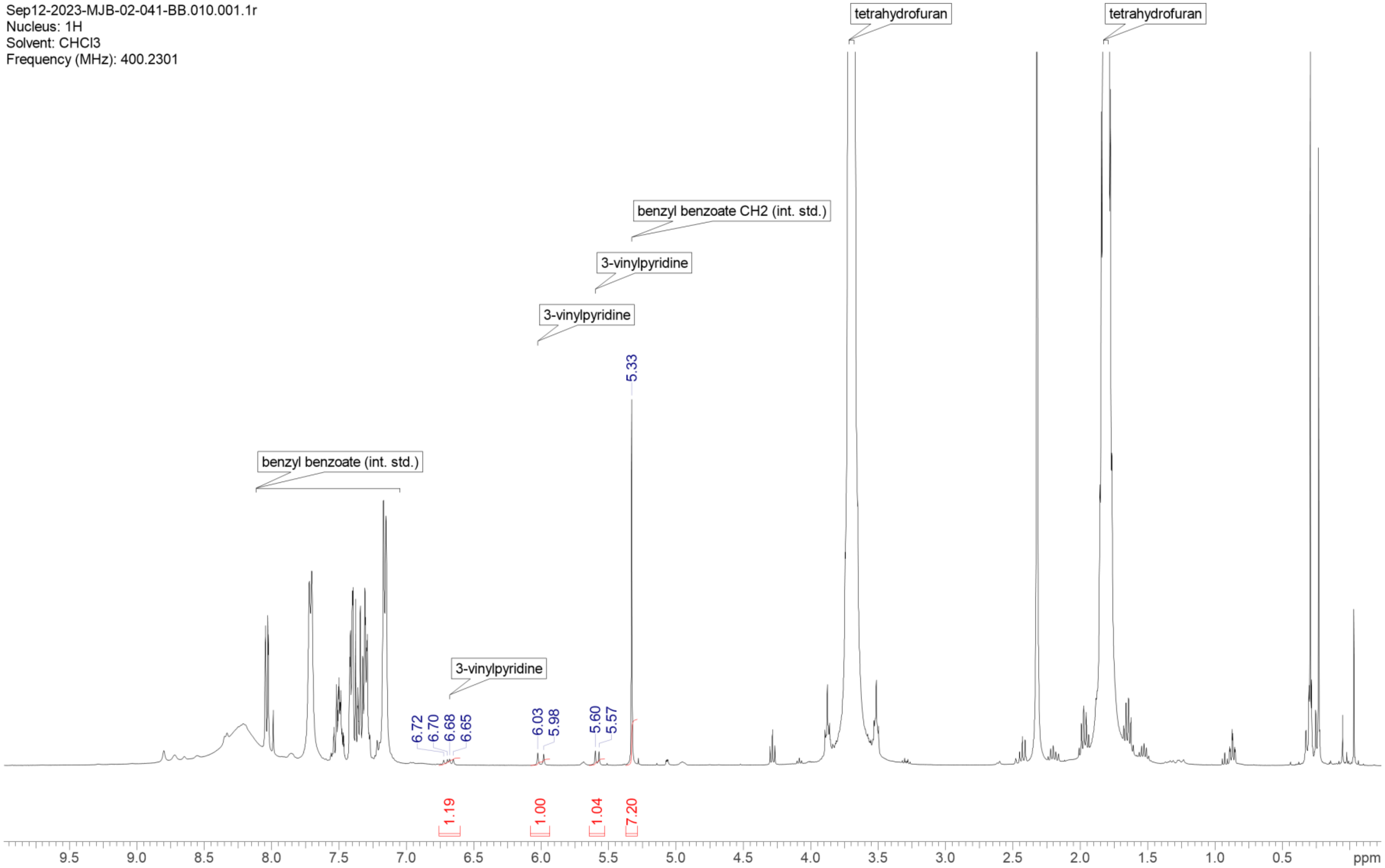

Figure S 25. ^{1}H NMR spectrum of 3-vinylpyridine (16, present in THF, benzyl benzoate internal standard): 50 mg solution in THF + 2.86 mg internal standard in $CDCl_3$

2-(Dimethylphenylsilyl)-1-(thiophen-2-yl)ethan-1-ol (23)

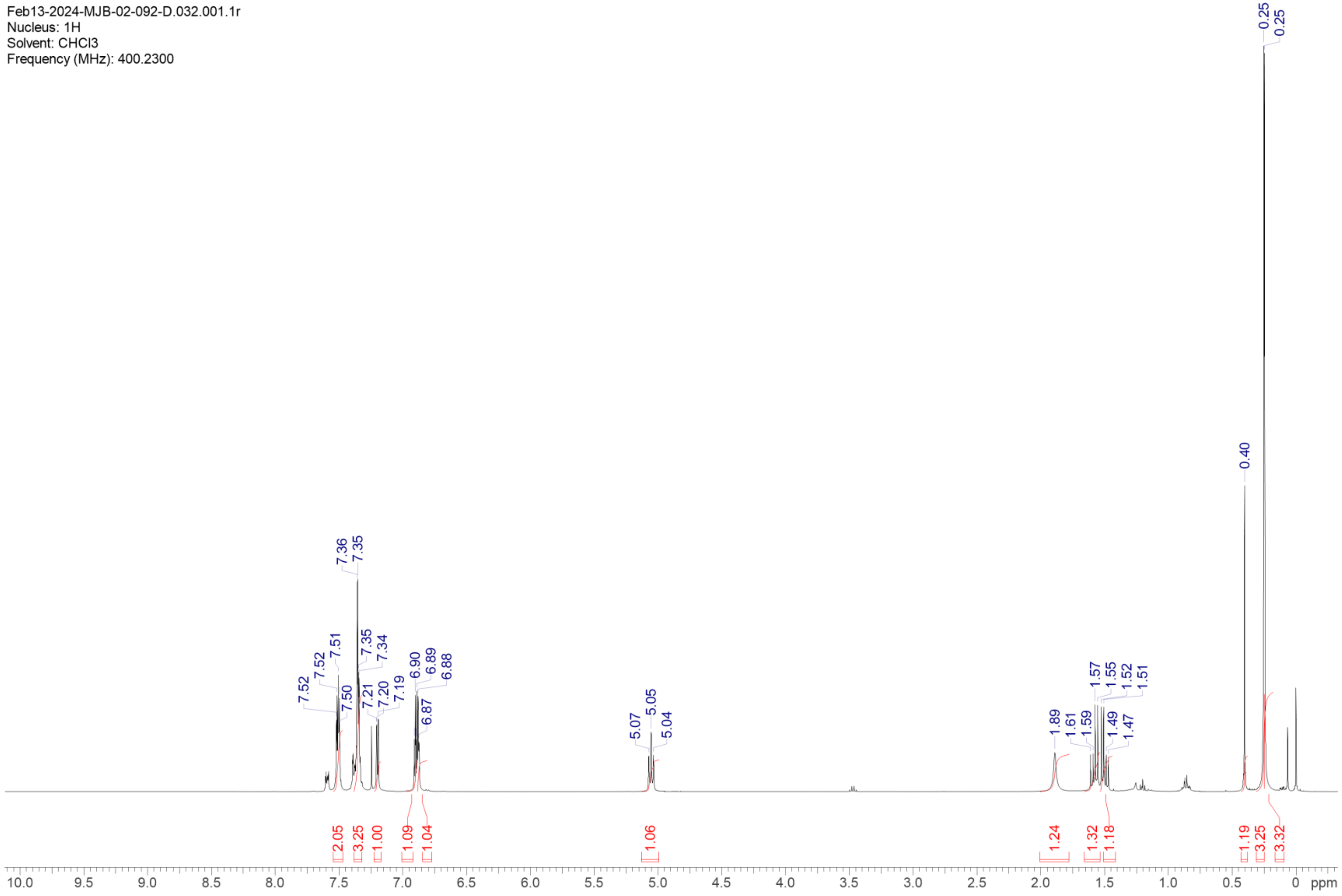

Figure S 26. ^{1}H NMR spectrum of 2-(dimethylphenylsilyl)-1-(thiophen-2-yl)ethan-1-ol (23)

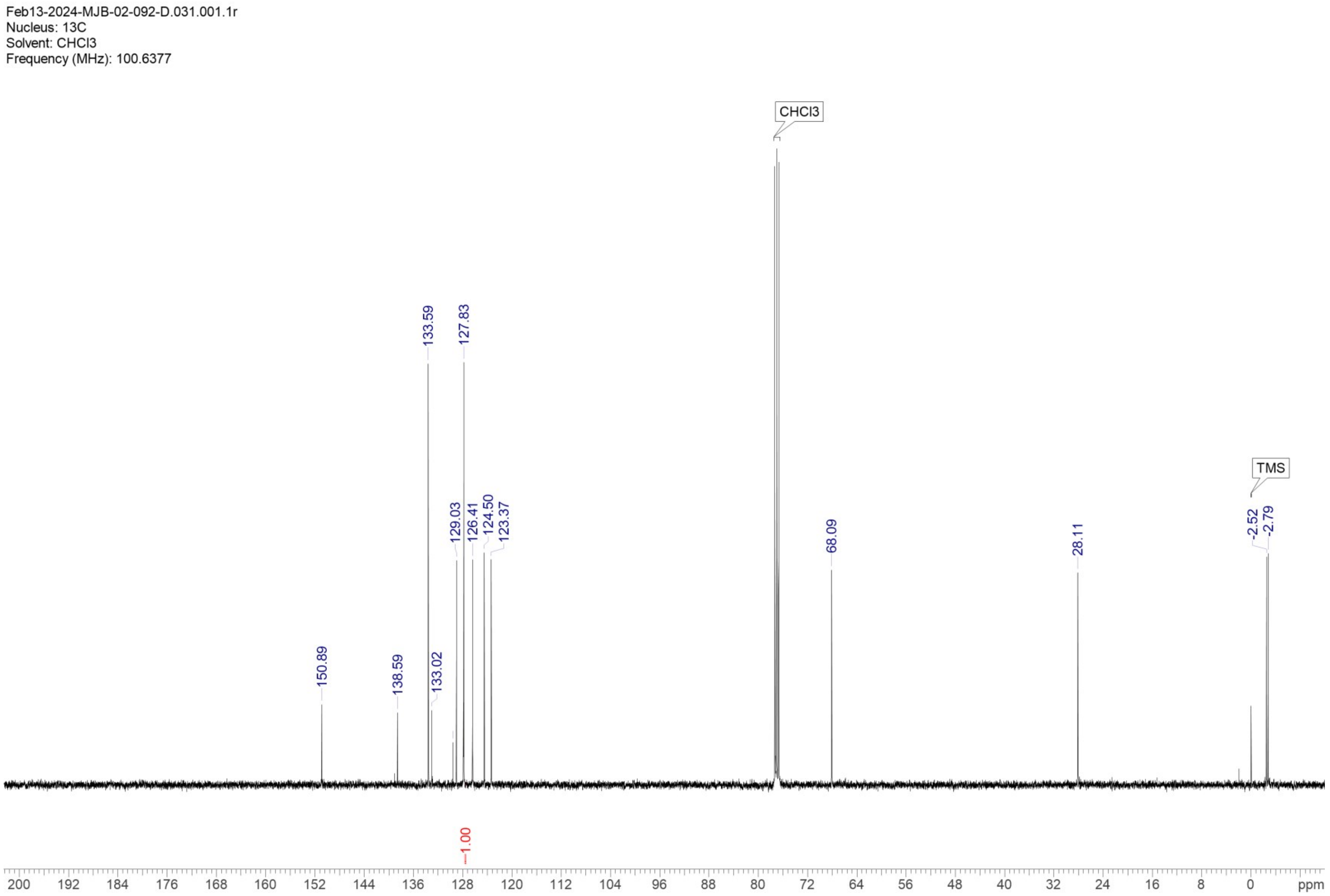

Figure S 27. ^{13}C **NMR spectrum of 2-(dimethylphenylsilyl)-1-(thiophen-2-yl)ethan-1-ol (23)**

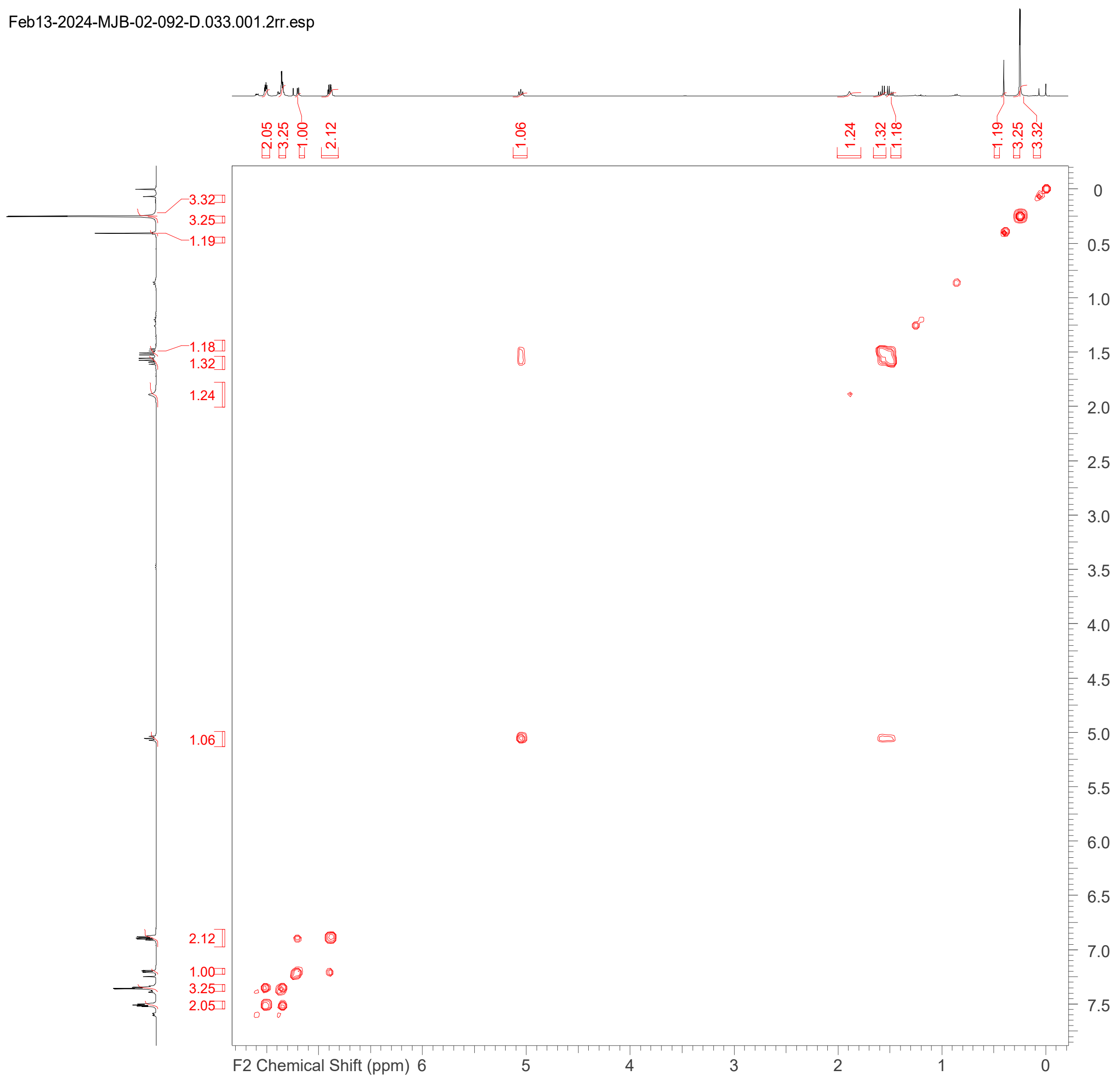

Figure S 28. ^{1}H COSY spectrum of 2-(dimethylphenylsilyl)-1-(thiophen-2-yl)ethan-1-ol (23)

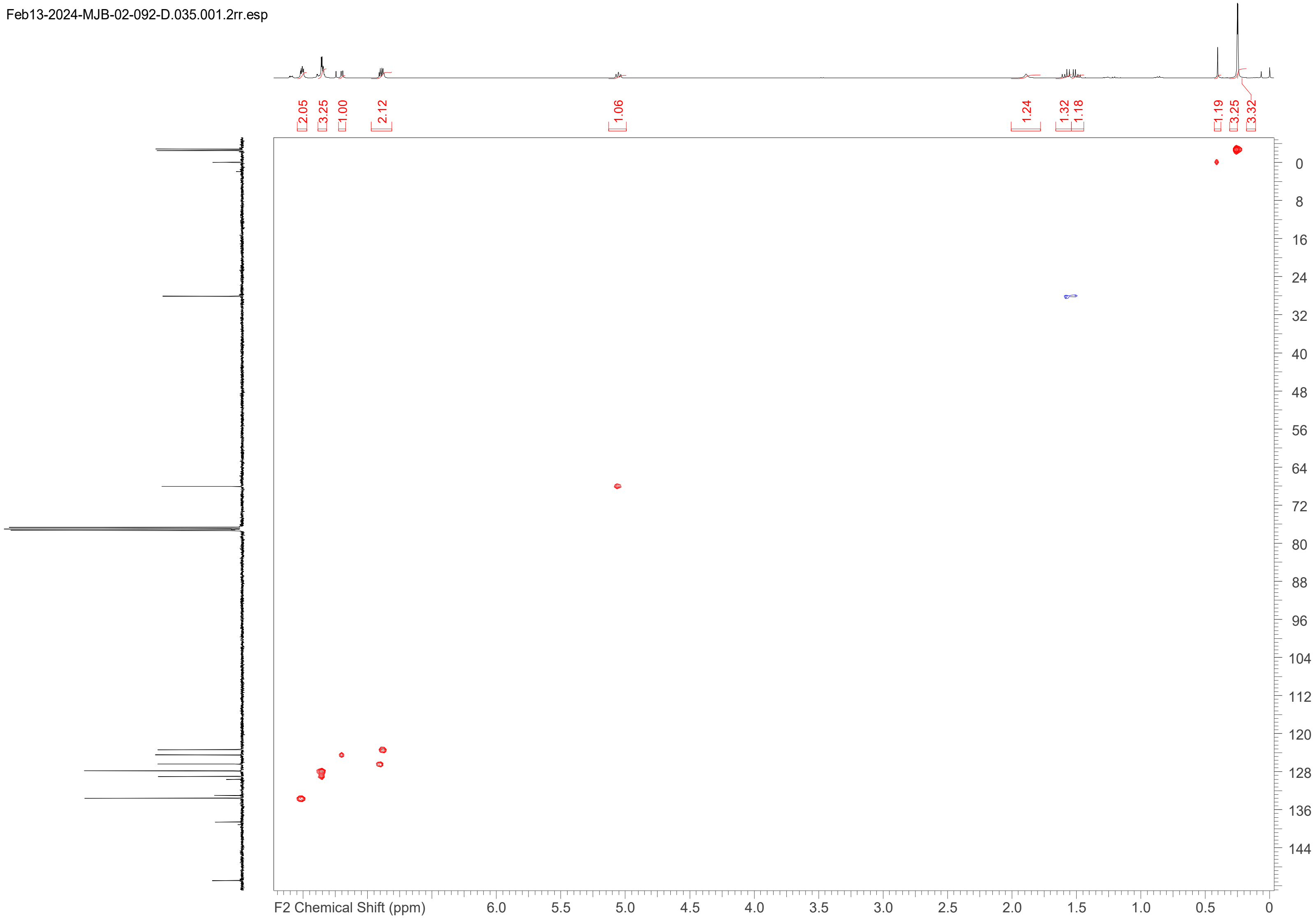

Figure S 29. HSQC spectrum of 2-(dimethylphenylsilyl)-1-(thiophen-2-yl)ethan-1-ol (23)

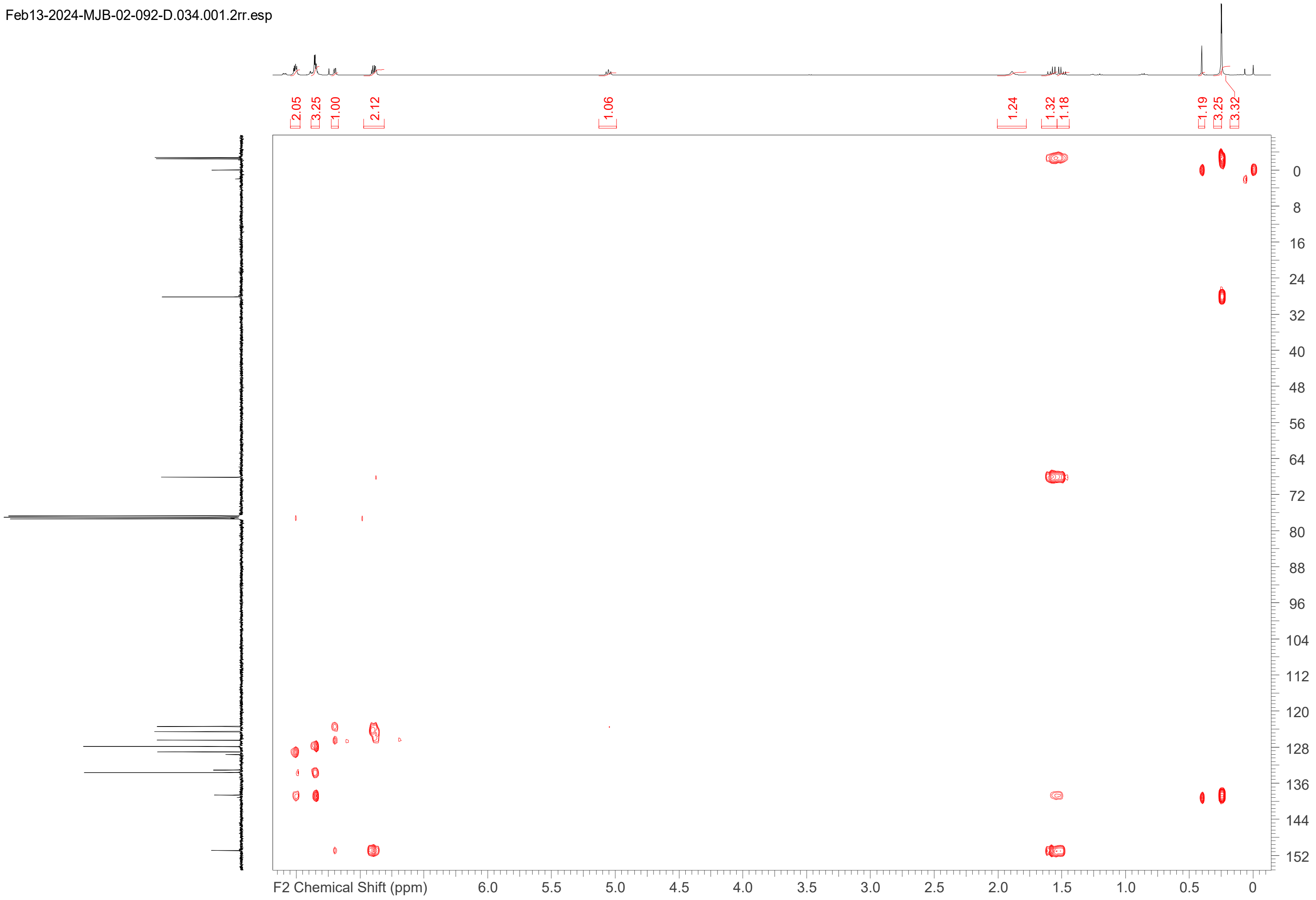

Figure S 30. HMBC spectrum of 2-(dimethylphenylsilyl)-1-(thiophen-2-yl)ethan-1-ol (23)

Matthew J. Burrell, John R. Varcoe, Daniel K. Whelligan

1-(Pyridine-3-yl)-2-(trimethylsilyl)ethan-1-ol (24)

Jan19-2024-MJB-02-087.020.001.1r
Nucleus: 1H
Solvent: CHCl3
Frequency (MHz): 400.2300

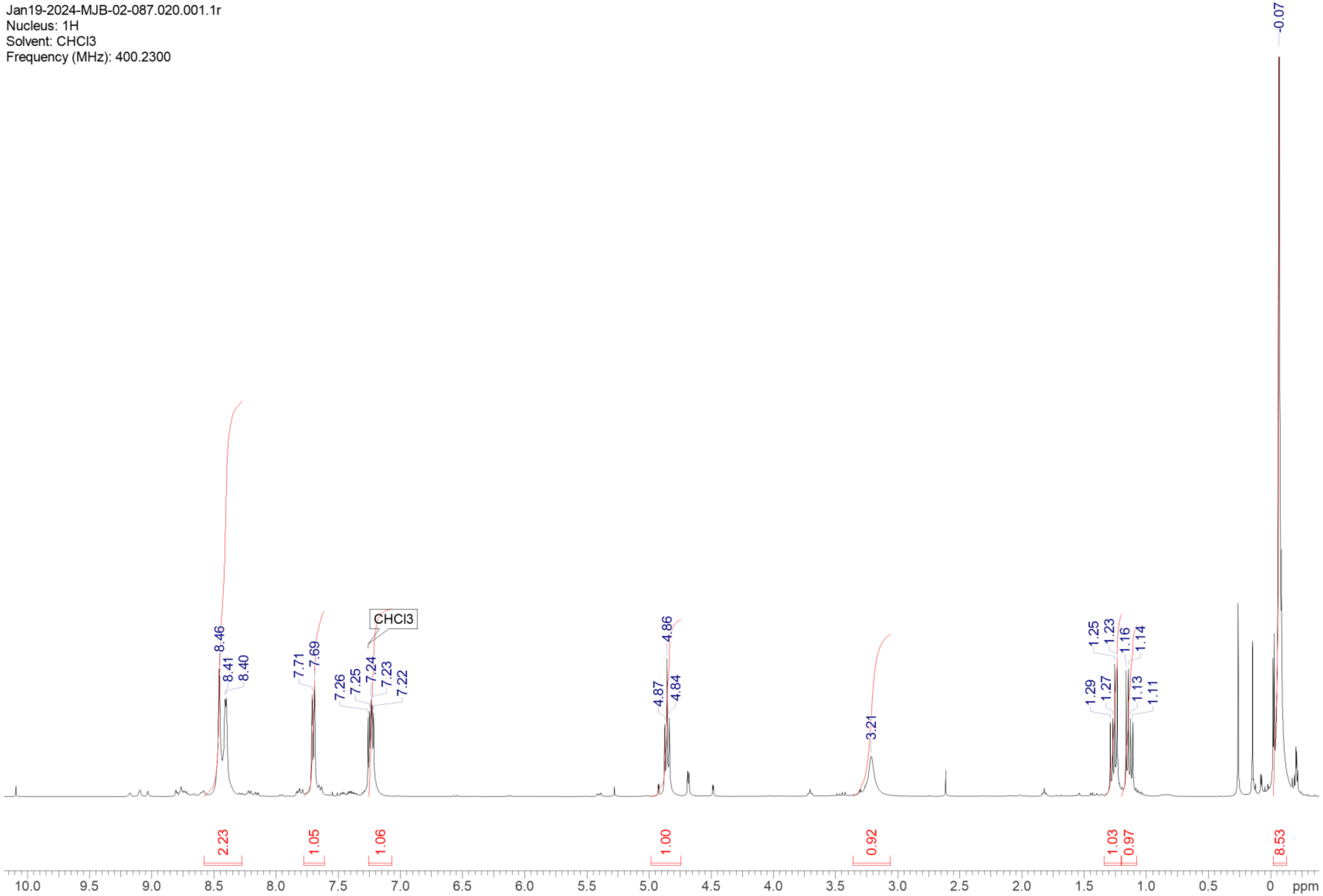

Figure S 31. ^{1}H NMR spectrum of 1-(pyridine-3-yl)-2-(trimethylsilyl)ethan-1-ol (24)

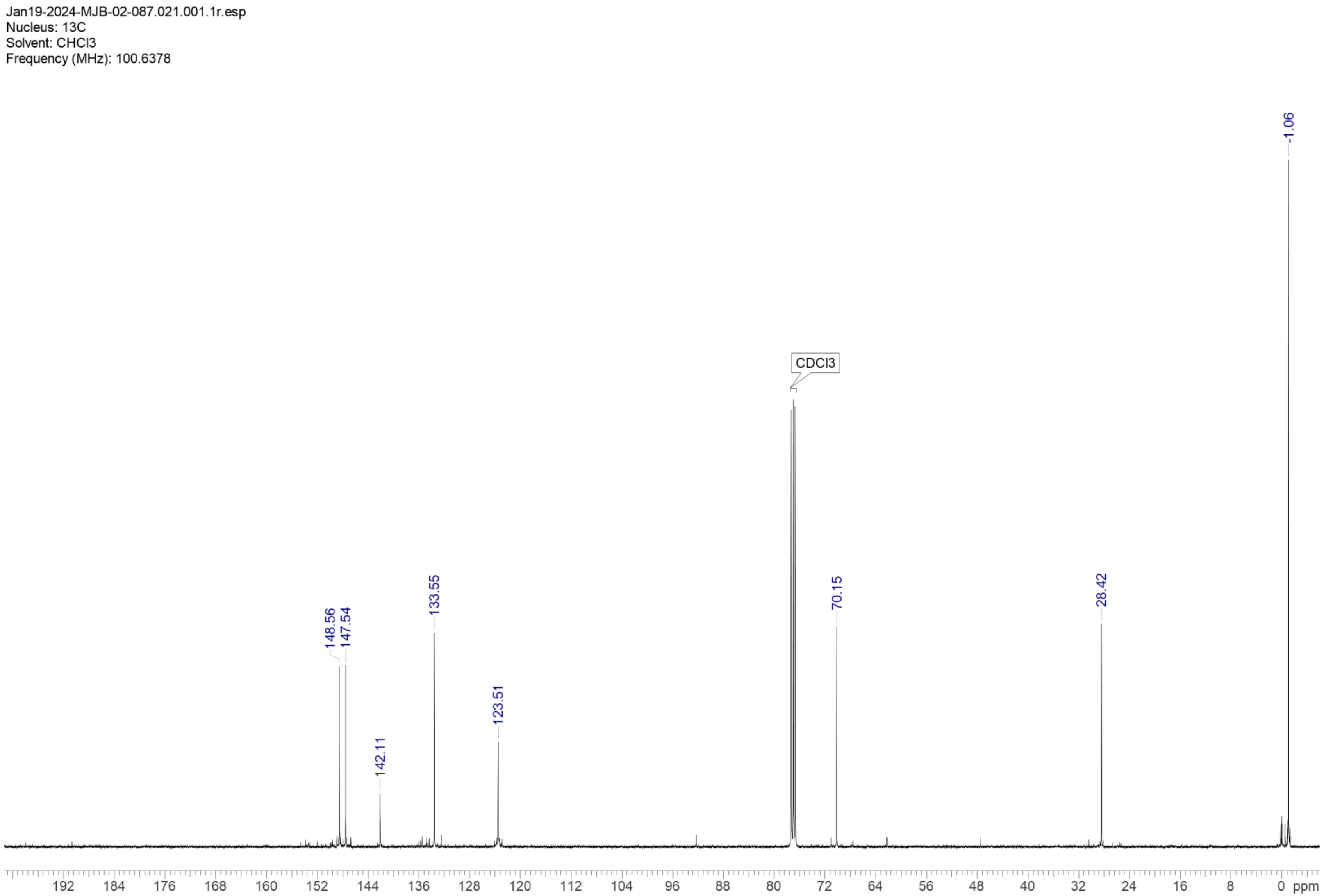

Figure S 32. ^{13}C **NMR spectrum of 1-(pyridine-3-yl)-2-(trimethylsilyl)ethan-1-ol (24)**

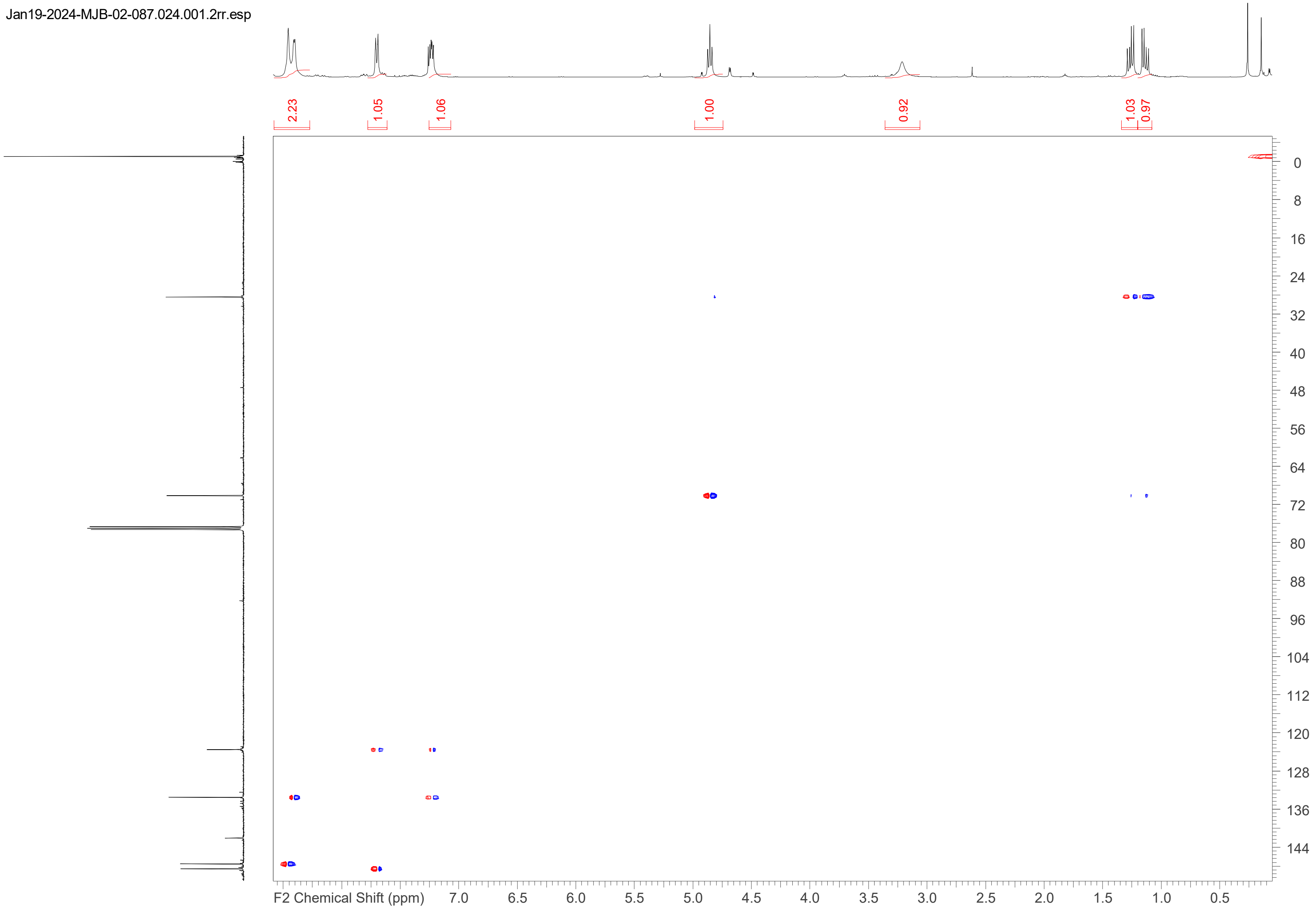

Figure S 33. HSQC spectrum of 1-(pyridine-3-yl)-2-(trimethylsilyl)ethan-1-ol (24)

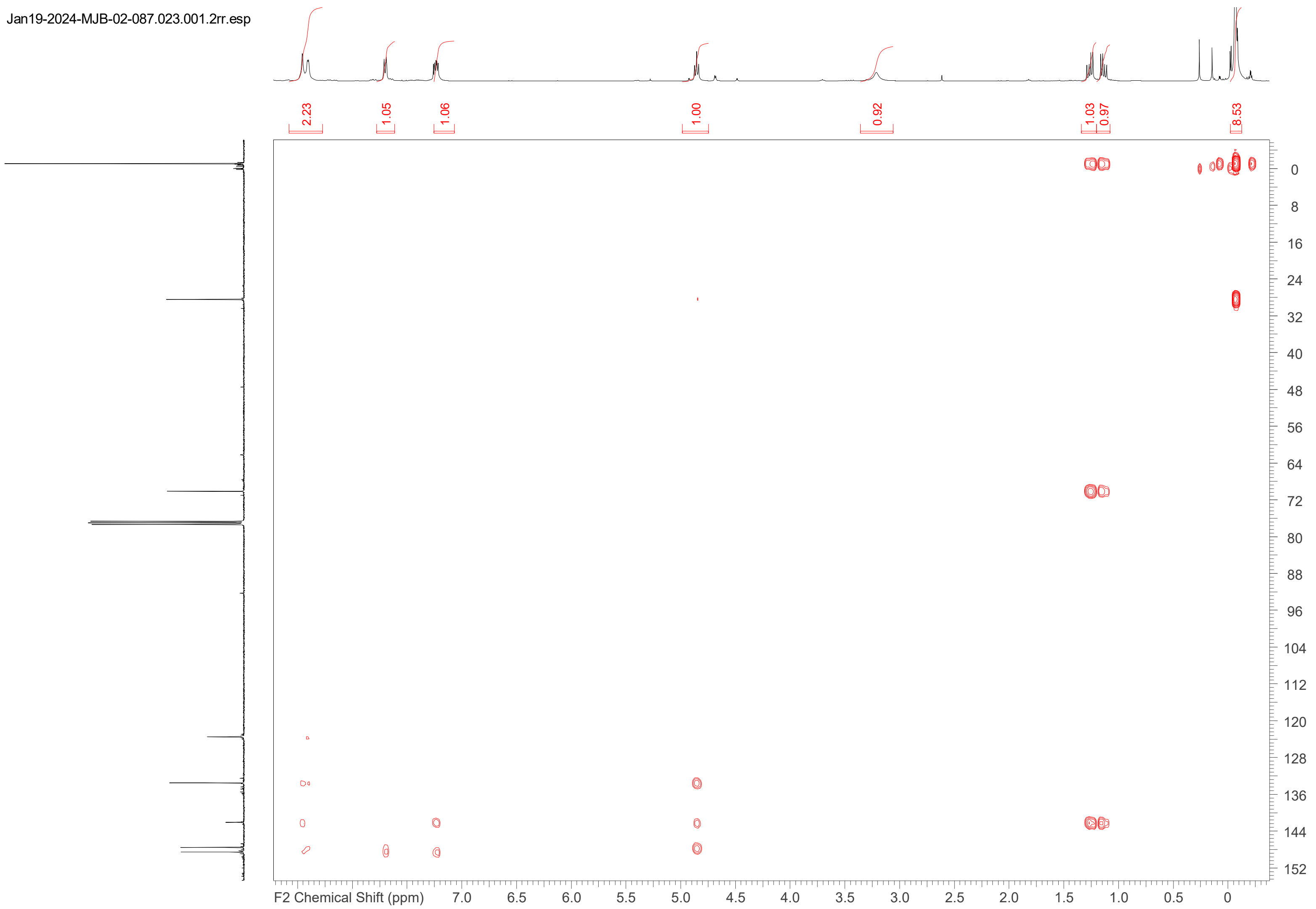

Figure S 34. HMBC spectrum of 1-(pyridine-3-yl)-2-(trimethylsilyl)ethan-1-ol (24)

References

(1) Chowdhury, D.; Goswami, S.; Krishna, G. R.; Mukherjee, A. *Dalton Trans.* **2024**, *53*, 3484.
(2) Grützke, M.; Weber, W.; Winter, M.; Nowak, S. *RSC Advances* **2016**, *6*, 57253.
(3) Wagh, R. B.; Nagarkar, J. M. *Tetrahedron Lett.* **2018**, *59*, 3443.
(4) Miller, J. M.; Ni, J. *J. Mass Spectrom.* **1996**, *31*, 16.
(5) Alan Aitken, R.; M. Armstrong, J.; J. Drysdale, M.; C. Ross, F.; M. Ryan, B. *J. Chem. Soc., Perkin Trans. 1* **1999**, 593.
(6) Luo, A. Y.; Bao, Y.; Cheng, X.-F.; Wang, X.-S. *Synthesis* **2017**, *49*, 3962.
(7) Abele, E.; Abele, R.; Gaukhman, A.; Lukevics, E. *Chem. Heterocycl. Compd.* **1998**, *34*, 40.
(8) Yu, A.-N.; Tan, Z.-W.; Wang, F.-S. *Food Chem.* **2012**, *132*, 1316.
(9) Ko, H.-H.; Tsao, L.-T.; Yu, K.-L.; Liu, C.-T.; Wang, J.-P.; Lin, C.-N. *Bioorg. Med. Chem.* **2003**, *11*, 105.
